# Supplementary material for: It Takes Two to Tango, Part II: Synthesis of A-Ring Functionalised Quinones Containing Two Redox-Active Centres with Antitumour Activities
Source: Molecules. 2023 Feb 27;28(5):2222. doi: 10.3390/molecules28052222 (PMC10005332; doi:10.3390/molecules28052222)
Supplement: Supplementary file 1 [file molecules-28-02222-s001.zip › 2. SI Triazoles.pdf]

molecules

Supplementary Material

It Takes Two to Tango, Part II: Synthesis of A-Ring Functionalised Quinones Containing Two Redox Active Centres with Antitumour Activities

Joyce C. Oliveira [1], Renato L. de Carvalho [1], Hugo G. S. Sampaio [1], João Honorato [2], Javier A. Ellena [2], Felipe T. Martins [3], João V. M. Pereira [4], Pedro M. S. Costa [4], Claudia Pessoa [4], Rafaela S. Ferreira [5], Maria H. Araújo [1], Claus Jacob [6] and Eufrânio N. da Silva Júnior [1,*]

[1] Institute of Exact Sciences, Department of Chemistry, Universidade Federal de Minas Gerais, UFMG, Belo Horizonte 31270-901, Brazil
[2] São Carlos Institute of Physics, Physics and Interdisciplinary Sciences Department, Universidade de São Paulo, USP, São Carlos 13560-970, Brazil
[3] Chemistry Institute, Universidade Federal de Goiás, UFG, Goiânia 74690-900, Brazil
[4] Department of Physiology and Pharmacology, Universidade Federal de Ceará, UFC, Fortaleza 60430-270, Brazil
[5] Biological Sciences Institute, Biochemistry and Immunology Department, Universidade Federal de Minas Gerais, UFMG, Belo Horizonte 31270-901, Brazil
[6] Division of Bioorganic Chemistry, School of Pharmacy, University of Saarland, 66123 Saarbruecken, Germany
* Correspondence: eufranio@ufmg.br

Citation: de Oliveira, J.C.; de Carvalho, R.L.; Sampaio, H.G.S.; Honorato, J.; Ellena, J.A.; Martins, F.T.; Pereira, J.V.M.; Costa, P.M.S.; Pessoa, C.; Ferreira, R.S.; et al. It Takes Two to Tango, PART II: Synthesis of A-ring Functionalised Quinones Containing Two Redox Active Centres with ANTITUMOUR activities. *Molecules* **2023**, *27*, x. https://doi.org/10.3390/molecules28052222

Academic Editor: Enrique Domínguez-Álvarez

Received: 23 December 2022
Revised: 26 January 2023
Accepted: 1 February 2023
Published: 27 February 2023

Copyright: © 2023 by the authors. Submitted for possible open access publication under the terms and conditions of the Creative Commons Attribution (CC BY) license (https://creativecommons.org/licenses/by/4.0/).

Table of Contents

General Remarks 2

Synthesis of Azide Precursors (4, 7 and 10) 2

Synthesis of A-ring Modified Quinoidal Substrates 5

General Procedure for Synthesis of Amino-alkynes (18a-e) 7

General Procedure for the Triazole Synthesis via a Copper-Catalysed 1,3-Dipolar Cycloaddition (19a-21e) 11

Characterization Data of Products 19a-21e 11

X-Ray Analysis 19

Antitumour Assays 21

References 24

^{1}H and ^{13}C NMR spectra 25

General Remarks

The solvents were dried using molecular sieves in inert atmosphere storage. Lawsone, *nor*-lapachol **(3),** lapachol **(8)**, juglone **(13)** and quinizarin (**16**) were used as purchased, without further purification. 5-Amino-1,4-naphthoquinone (**11**) was synthesised according to a procedure already discussed in the literature [46]. The reaction concentration is expressed in molar (M), this concentration was calculated by the ratio of the amount of the main reactant (limiting agent) in mmol and the volume of solvent applied, in mL. Presented yields refer to isolated compounds, estimated to be >95% pure as determined by ^{1}H-NMR. TLC: Merck, TLC Silica gel 60 F_{254}, detection at 254 nm. Infrared spectra were recorded on a Bruker ATR FT-IR Alpha device and IR Prestige-21 Shimadzu using KBr plates. Mass-spectra: EI-MS: Jeol AccuTOF at 70 eV; ESI-MS: Bruker maXis and MicrOTOF. High resolution mass spectrometry (HRMS): Bruker maXis, Bruker MicrOTOF and Jeol AccuTOF. Melting points: Büchi 540 capillary melting point apparatus, values are uncorrected. NMR spectra were recorded on Avance III HD 400, Avance III 400, and Avance NEO 600 instruments, if not otherwise specified, chemical shifts (δ) are provided in ppm. ^{13}C-NMR shifts are classified as: C_q (non-hydrogenated carbon), CH, CH_2, CH_3, indicating the nature of the carbon assigned, according to what observed by DEPT or ATP analysis. All structure names were given under IUPAC rules by CS ChemDraw Ultra program. Single crystals were recrystallised from a mixture of acetonitrile and petroleum ether using a system of vapor diffusion. The crystals were analysed on a XtaLAB Synergy Rigaku four-circle diffractometer. Using Olex2 [47], the structures were solved with the XT [48] structure solution program using Intrinsic Phasing and refined with the XL [49] refinement package using Least Squares minimisation.

Synthesis of Azide Precursors (4, 7 and 10)

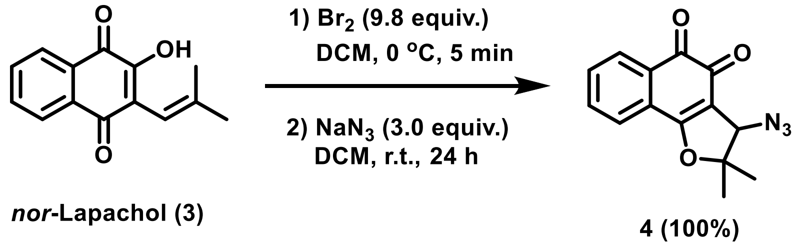

3-azido-2,2-dimethyl-2,3-dihydronaphtho[1,2-*b*]furan-4,5-dione (4): In a 100 mL rounded-bottom flask, nor-lapachol (**3**, 456 mg, 2.0 mmol) and DCM (30 mL) were added. The mixture was cooled down to 0 °C, followed by the careful addition of bromine (1.0 mL, 3.12 g, 19.5 mmol). The reaction was kept under continuous stirring at 0 °C for 5 minutes. The excess of bromine, along with the solvent, was removed under reduced pressure, resulting in an orange solid. This mixture was directly used without further purification in the next step, by the addition of DCM (10 mL) and sodium azide (390 mg, 6.0 mmol). The reaction was kept under continuous stirring at room temperature for 24 h. The final crude was suspended in 15 mL of distilled water, extracted with ethyl acetate (3 x 15 mL) and dried over Na_2SO_4. Column chromatography (*n*-hexane/AcOEt 8:2) on silica gel led to the desired azide **4** (538 mg, 100%) as an orange solid. **^{1}H NMR** (400 MHz, $CDCl_3$) δ = 8.15-8.13 (m, 1H), 7.72-7.65 (m, 3H), 4.78 (s, 1H), 1.68 (s, 3H), 1.56 (s, 3H). **^{13}C NMR** (100 MHz, $CDCl_3$) δ = 180.6 (C_q), 175.5 (C_q), 170.4 (C_q), 134.8 (CH), 133.1 (CH), 131.4 (C_q), 129.9 (CH), 127.0 (C_q), 125.4 (CH), 113.8 (C_q), 95.8 (C_q), 67.7 (CH), 27.4 (CH_3), 22.2 (CH_3). **IR** (KBr): $\tilde{\nu}$ = 3417, 2965, 2935, 2110, 1697, 1654, 1618, 1571, 1406, 1266, 1217 cm^{-1}. **m.p.** (°C) = 200-202.
The analytical data are in accordance with those reported in the literature [43].

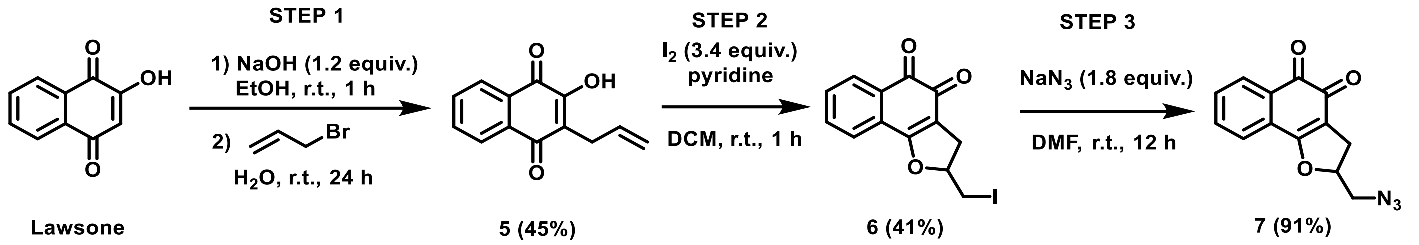

STEP 1

2-allyl-3-hydroxynaphthalene-1,4-dione (5): Sodium hydroxide (1.4 g, 35.0 mmol) was dissolved in ethanol (50 mL) in a 250 mL rounded bottom flask. Lawsone (5.0 g, 29.0 mmol) was added to the mixture, and the final solution was stirred for 1 h at room temperature. The red precipitate (sodium lawsonate) was filtered off, washed with diethyl ether, and dried in a 70 °C oven. The achieved sodium lawsonate (3.0 g, 15 mmol) and allyl bromide (30.0 mL) were added to a 250 mL rounded-bottom flask, and the mixture was stirred at room temperature for 1 h. Distilled water (70 mL) was added, and the final mixture was stirred for further 24 h at room temperature. The solution was diluted with additional 30 mL of water and extracted with ethyl acetate (3 x 15 mL). The organic phase was dried over Na_2SO_4 and concentrated under reduced pressure. Column chromatography (*n*-hexane/AcOEt 8:2) on silica gel led to the desired product **5** (1.44 g, 45%) as a yellow solid. **^{1}H NMR** (400 MHz, $CDCl_3$) δ = 8.11 (dd, *J* = 7.6, 0.4 Hz, 1H), 8.07 (dd, *J* = 7.6, 0.6 Hz, 1H), 7.75 (td, *J* = 7.5, 1.0 Hz, 1H), 7.67 (td, *J* = 7.4, 1.0 Hz, 1H), 7.41 (s, 1H), 5.95-5.85 (m, 1H), 5.17 (dd, *J* = 17.1, 1.4 Hz, 1H), 5.04 (dd, *J* = 10.0, 1.0 Hz, 1H), 3.37 (br s, 1H), 3.35 (br s, 1H). **^{13}C NMR** (100 MHz, $CDCl_3$) δ =184.3 (C_q), 181.6 (C_q), 153.3 (C_q), 135.1 (CH), 133.9 (CH), 133.1 (CH), 132.9 (C_q), 129.5 (C_q), 127.0 (CH), 126.3 (CH), 122.0 (C_q), 116.6 (CH_2), 27.6 (CH_2). **IR** (KBr): $\tilde{\nu}$ = 3355, 1644, 1589, 1371, 1351, 1272, 1230, 729 cm^{-1}. **m.p.** (°C) = 112-113.

The analytical data are in accordance with those reported in the literature [50].

STEP 2

2-(iodomethyl)-2,3-dihydronaphtho[1,2-*b*]furan-4,5-dione (6): Compound **5** (1.0 g, 5.9 mmol) was dissolved in DCM (100 mL) in a 250 mL rounded bottom flask. A solution of iodine (7.3 g, 20.0 mmol) in DCM (30 mL) and pyridine (4 mL) was added to the mixture, and the final solution was stirred for 1 h at room temperature, followed by the addition of 100 mL of cold water. The organic phase was separated, washed with a Na_2CO_3 10% solution (3 x 50 mL) and water (3 x 50 mL), and dried over Na_2SO_4. Column chromatography (*n*-hexane/AcOEt 8:2) on silica gel led to the desired product **6** (823 mg, 41%) as a red solid. **^{1}H NMR** (400 MHz, $CDCl_3$) δ = 8.06 (d, *J* = 7.4 Hz, 1H), 7.68-7.63 (m, 2H), 7.60-7.56 (m, 1H), 5.18-5.11 (m, 1H), 3.51 (s, 1H), 3.49 (d, *J* = 0.8 Hz, 1H), 3.30 (dd, *J* = 16.0, 10.0 Hz, 1H), 2.92 (dd, *J* = 16.0, 6.8 Hz, 1H). **^{13}C NMR** (100 MHz, $CDCl_3$) δ = 180.9 (C_q), 175.4 (C_q), 169.3 (C_q), 134.8 (CH), 132.3 (CH), 130.7 (C_q), 129.7 (CH), 127.3 (C_q), 124.7 (CH), 115.0 (C_q), 85.4 (CH), 33.2 (CH_2), 7.4 (CH_2). **IR** (ATR): $\tilde{\nu}$ = 3354, 2366, 1686, 1644, 1609, 1582, 1569, 1408, 1348, 1281, 1240, 1222, 1148, 882, 666 cm^{-1}. **m.p.** (°C) = 145-147.

The analytical data are in accordance with those reported in the literature [51].

STEP 3

2-(azidomethyl)-2,3-dihydronaphtho[1,2-*b*]furan-4,5-dione (7): Compound **6** (610 mg, 1.8 mmol) and sodium azide (216 mg, 3.3 mmol) were dissolved in DMF (10 mL) in a 50 mL rounded bottom flask. The mixture was stirred for 12 h at room temperature, followed by extraction with DCM (3 x 15 mL). The organic phase was washed with distilled water (15 mL) and dried over Na_2SO_4. Column chromatography (*n*-hexane/AcOEt 8:2) on silica gel led to the desired azide **7** (418 mg, 91%) as an orange solid. **^{1}H NMR** (400 MHz, $CDCl_3$) δ = 8.06 (d, *J* = 7.6 Hz, 1H), 7.69-7.64 (m, 2H), 7.62-7.58 (m, 1H), 5.34-5.28 (m, 1H), 3.70-3.60 (m, 2H), 3.26 (dd, *J* = 15.6, 10.4 Hz, 1H), 2.93 (dd, *J* = 15.6, 7.2 Hz, 1H). **^{13}C NMR** (100 MHz,

$CDCl_3$) δ = 180.8 (C_q), 175.4 (C_q), 169.4 (C_q), 134.8 (CH), 132.3 (CH), 130.6 (C_q), 129.7 (CH), 127.1 (C_q), 124.7 (CH), 115.2 (C_q), 85.8 (CH), 54.2 (CH_2), 29.5 (CH_2). **IR** (KBr): $\tilde{\nu}$ = 3369, 2974, 2105, 1690, 1660, 1588, 1408, 1242, 1216 cm^{-1}. **m.p.** (°C) = 172-174.
The analytical data are in accordance with those reported in the literature [44].

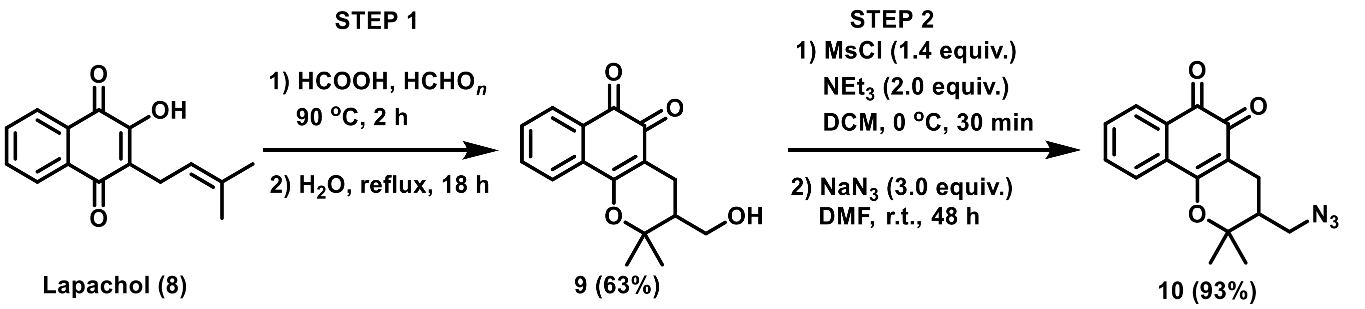

STEP 1
3-(hydroxymethyl)-2,2-dimethyl-3,4-dihydro-2*H*-benzo[*h*]chromene-5,6-dione (9): Formic acid (5.0 mL) was placed in a 50 mL rounded-bottom flask and heated until reaching 90 °C. Paraformaldehyde (264 mg, 8.9 mmol) and lapachol (**8**, 1.1 g, 4.4 mmol) were added, and the mixture was stirred at 90 °C for 2 h. Distilled water (10 mL) was added to the solution and the reaction was kept under reflux for additional 12 h. The solution was cooled to room temperature and neutralised with Na_2CO_3 (7.4 g) carefully added. The mixture was extracted with ethyl acetate (3 x 150 mL) and the organic phase was dried over Na_2SO_4. Column chromatography (*n*-hexane/AcOEt 8:2) on silica gel led to the desired product **9** (755 mg, 63%) as an orange solid. **^{1}H NMR** (400 MHz, $CDCl_3$) δ = 8.00 (d, *J* = 7.6 Hz, 1H), 7.78 (d, *J* = 8.0 Hz, 1H), 7.62 (t, *J* = 7.6 Hz, 1H), 7.48 (t, *J* = 7.2 Hz, 1H), 3.86 (dd, *J* = 11.2, 5.2 Hz, 1H), 3.63 (dd, *J* = 10.8, 7.2 Hz, 1H), 2.78 (dd, *J* = 17.6, 5.6 Hz, 1H), 2.51 (br s, 1H), 2.31 (dd, *J* = 17.6, 10.0 Hz, 1H), 2.07-2.00 (m, 1H), 1.60 (s, 3H), 1.34 (s, 3H). **^{13}C NMR** (400 MHz, $CDCl_3$) δ = 179.9 (C_q), 178.6 (C_q), 162.0 (C_q), 135.0 (CH), 132.4 (C_q), 130.9 (CH), 130.2 (C_q), 128.7 (CH), 124.3 (CH), 112.7 (C_q), 81.9 (C_q), 63.0 (CH_2), 42.6 (CH), 27.8 (CH_3), 22.2 (CH_3), 19.8 (CH_2). **IR** (KBr): $\tilde{\nu}$ = 3519, 3464, 2981, 2933, 1695, 1648, 1602, 1571, 1398, 1126 cm^{-1}. **m.p.** (°C) = 145-148.
The analytical data are in accordance with those reported in the literature [32].

STEP 2
3-(azidomethyl)-2,2-dimethyl-3,4-dihydro-2*H*-benzo[*h*]chromene-5,6-dione (10): Compound **9** (272 mg, 1.0 mmol) was dissolved in DCM (10 mL) at 0 °C in a 25 mL rounded-bottom flask. Triethylamine (280 μL, 2.0 mmol) and methanesulfonyl chloride (120 μL, 1.4 mmol) were added to the solution, which was stirred for 30 minutes at 0 °C. The solvent was removed under reduced pressure and redissolved in DMF (10 mL). Sodium azide (200 mg, 3.1 mmol) was added, and the final mixture was stirred for additional 48 h at room temperature, followed by extraction with ethyl acetate (3 x 150 mL). The organic phase was dried over Na_2SO_4. Column chromatography (*n*-hexane/AcOEt 2:1) on silica gel led to the desired azide **10** (276 mg, 93%) as a dark orange solid. **^{1}H NMR** (400 MHz, $CDCl_3$) δ = 8.04 (d, *J* = 7.6 Hz, 1H), 7.77 (d, *J* = 7.6 Hz, 1H), 7.64 (dt, *J* = 7.6, 1.2 Hz, 1H), 7.50 (dt, *J* = 7.6, 0.8 Hz, 1H), 3.56 (dd, *J* = 14.4, 5.2 Hz, 1H), 3.24 (dd, *J* = 12.4, 8.0, 1H), 2.80 (dd, *J* = 18.0, 5.6 Hz, 1H), 2.32 (dd, *J* = 18.0, 9.6 Hz, 1H), 2.08-2.01 (m, 1H), 1.58 (s, 3H), 1.35 (s, 3H). **^{13}C NMR** (100 MHz, $CDCl_3$) δ = 179.6 (C_q), 178.5 (C_q), 161.5 (C_q), 135.0 (CH), 132.1 (C_q), 131.0 (CH), 130.2 (C_q), 128.8 (CH), 124.2 (CH), 112.0 (C_q), 80.9 (C_q), 52.3 (CH_2), 40.1 (CH), 27.4 (CH_3), 22.1 (CH_3), 20.8 (CH_2). **IR** (KBr): $\tilde{\nu}$ = 3431, 2928, 2097, 1693, 1606, 1589, 1392, 1261, 1231, 1130 cm^{-1}. **m.p.** (°C) = 103-106.
The analytical data are in accordance with those reported in the literature [32].

Synthesis of A-ring Modified Quinoidal Substrates

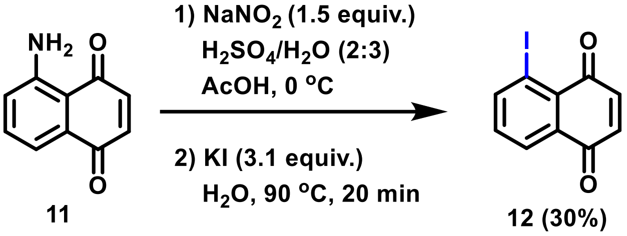

5-Iodo-1,4-naphthoquinone (12): 5-Amino-1,4-naphthoquinone (**11**, 1.0 g, 5.77 mmol) and glacial acetic acid (31.4 mL) were placed in a 250 mL rounded-bottom flask under continuous stirring at room temperature. A mixture of sulfuric acid/water 2:1 (24 mL) was carefully added, and the final mixture was transferred to a 250 mL beaker, with extra care for a complete removal of residual solid. A solution of sodium nitrite (600 mg, 8.65 mmol) in 1.0 mL of water was added to the reaction under continuous stirring at 0 °C. The obtained solution was then quickly verted onto a solution of potassium iodide (3.0 g, 17.9 mmol) in distilled water (80 mL) in a 1.0 L beaker. The reaction was kept under stirring at 90 °C for 20 minutes. After completion of the reaction, the final mixture was kept at -22 °C in a fridge for 18 hours, from which a precipitate was formed and, subsequentially, filtered off. A column chromatography (silica gel, toluene) led to the obtention of 5-iodo-1,4-naphthoquinone (**12**, 500 mg, 30%) as a red solid. **^{1}H NMR** (400 MHz, $CDCl_3$) δ = 8.36 (d, J = 7.8 Hz, 1H), 8.15 (d, J = 7.8 Hz, 1H), 7.35 (t, J = 7.8 Hz, 1H), 7.02 (d, J = 10.3 Hz, 1H), 6.94 (d, J = 10.3 Hz, 1H). **^{13}C NMR** (100 MHz, $CDCl_3$) δ = 183.7 (C_q), 183.3 (C_q), 148.3 (CH), 139.9 (CH), 137.2 (CH), 134.4 (C_q), 133.8 (CH), 130.8 (C_q), 127.7 (CH), 92.9 (C_q). **IR** (ATR): $\tilde{\nu}$ = 1665, 1613, 1567, 1319, 782, 563 cm^{-1}. **m.p.** (°C) = 171-172; **HRMS** (ESI): Calcd. for $C_{10}H_6IO_2$ $[M+H]^+$ 284.9407, found 284.9412.
The analytical data are in accordance with those reported in the literature [36].

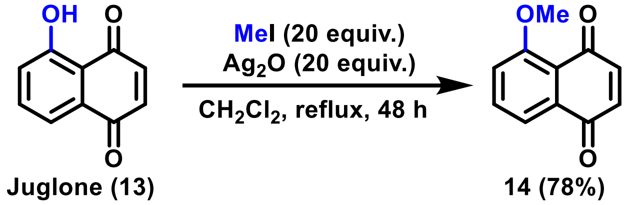

5-Methoxy-1,4-naphthoquinone (14): Juglone (**13**, 174 mg, 1.0 mmol), iodomethane (125 µL, 285 mg, 2.0 mmol) and Ag_2O (463 mg, 2.0 mmol) were dissolved in dichloromethane (30 mL) in a 125 mL rounded-bottom flask. The solution was kept under reflux for 48 h. The final solution was filtered through a pad of celite and washed with dichloromethane. The solution was then concentrated under reduced pressure and purified through column chromatography (n-hexane/AcOEt 8:2) to provide 5-methoxy-1,4-naphthoquinone (**14**, 147 mg, 78%) as a yellow solid. **^{1}H NMR** (400 MHz, $CDCl_3$) δ = 7.72-7.65 (m, 2H), 7.30 (dd, J = 8.1, 1.3 Hz, 1H), 6.87-6.82 (m, 2H), 3.99 (s, 3H). **^{13}C NMR** (100 MHz, $CDCl_3$) δ = 185.4 (C_q), 184.5 (C_q), 159.8 (C_q), 141.0 (CH), 136.4 (CH), 135.2 (CH), 134.2 (C_q), 119.9 (C_q), 119.3 (CH), 118.1 (CH), 56.7 (CH_3). **IR** (ATR): $\tilde{\nu}$ = 1651, 1613, 1581, 1469, 1442, 1376, 1296 cm^{-1}. **m.p.** (°C) = 180-182; **HRMS** (ESI): Calcd. for $C_{11}H_9O_3$ $[M+H]^+$ 189.0546, found 189.0546.
The analytical data are in accordance with those reported in the literature [52].

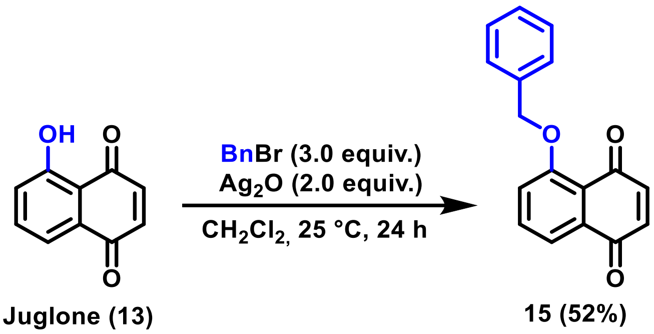

5-Benzyloxy-1,4-naphthoquinone (15): Juglone (**13**, 174 mg, 1.0 mmol), benzyl bromide (513 mg, 3.0 mmol) and Ag_2O (463 mg, 2.0 mmol) were dissolved in dichloromethane (30 mL) in a 125 mL rounded-bottom flask. The solution was kept under stirring at 25 °C for 24 h. The final solution was filtered through a pad of celite and washed with dichloromethane. The solution was then concentrated under reduced pressure and purified through column chromatography (*n*-hexane/AcOEt 8:2) to provide 5-methoxy-1,4-naphthoquinone (**15**, 137 mg, 52%) as a red solid. **^{1}H NMR** (400 MHz, $CDCl_3$) δ = 7.71 (dd, J = 7.6, 0.8 Hz, 1H), 7.62 (t, J = 8.4 Hz, 1H), 7.57 (d, J = 7.2 Hz, 2H), 7.40 (t, J = 7.2 Hz, 2H), 7.33-7.31 (m, 2H), 6.86, (s, 2H), 5.27 (s, 2H). **^{13}C NMR** (100 MHz, $CDCl_3$) δ = 185.3 (C_q), 184.2 (C_q), 158.6 (C_q), 140.9 (CH), 136.3 (CH), 136.1 (C_q), 134.9 (CH), 134.2 (C_q), 128.8 (CH), 128.0 (CH), 126.7 (CH), 120.3 (C_q), 119.7 (CH), 119.5 (CH), 70.9 (CH_2). **IR** (KBr): $\tilde{\nu}$ = 1747, 1660, 1614, 1582, 1497, 1454, 1254, 1023, 733, 697 cm^{-1}. **m.p.** (°C) = 30-32; **HRMS** (ESI): Calcd. for $C_{17}H_{12}O_3Na$ [M+Na]$^+$ 287.0679, found 287.0677.
The analytical data are in accordance with those reported in the literature [53-54].

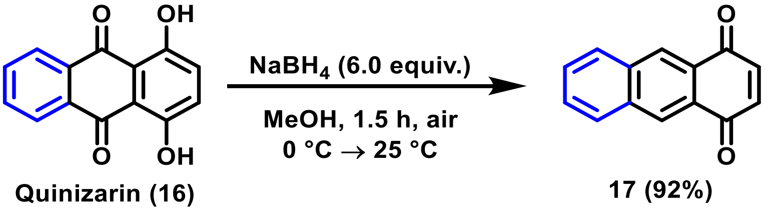

1,4-Antraquinone (17): Quinizarin (**16**, 989 mg, 4.12 mmol) was dissolved in methanol (19 mL) at 0 °C. Sodium borohydride (945 mg, 25.0 mmol) was added carefully. The reaction was stirred for 90 min at 0 °C. An aq. solution of hydrochloric acid (6m, 18 mL) was added, and the precipitate was filtered off and washed with water to afford 1,4-antraquinone **17** as a brown solid (791 mg, 92%). **^{1}H NMR** (400 MHz, $CDCl_3$) δ = 8.55 (s, 2H), 8.01 (s, 2H), 7.66 (s, 2H), 7.03 (s, 2H). **^{13}C NMR** (100 MHz, $CDCl_3$) δ = 184.8 (C_q), 140.2 (CH), 134.9 (C_q), 130.4 (CH), 129.8 (CH), 129.0 (CH), 128.5 (C_q). **IR** (ATR): $\tilde{\nu}$ = 3052, 1665, 1614, 1596, 1448, 1293 cm^{-1}. **m.p.** (°C) = 212-216. **HRMS** (ESI): Calcd. for $C_{14}H_9O_2$ [M+H]$^+$ 209.0597, found 209.0604.
The analytical data are in accordance with those reported in the literature [55].

General Procedure for Synthesis of Amino-alkynes (18a-e)

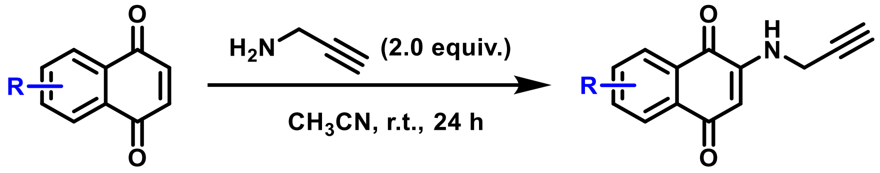

18a-e

The corresponding quinone (1.0 mmol) was dissolved in acetonitrile (3.0 mL, 0.3 m) at room temperature in a 10 mL rounded-bottom flask. *N*-propargylamine (128 μL, 110.2 mg, 2.0 mmol) was added to the mixture and it was kept under continuous stirring over 24 h at room temperature. The respective amino-alkyne was obtained by column chromatography (*n*-hexane/EtOAc 8:2). The correct position of the propargylamine substituent was determined over bidimensional NMR spectra analysis.

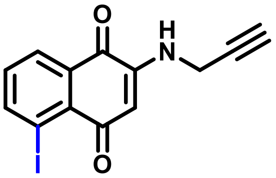

5-iodo-2-(prop-2-yn-1-ylamino)naphthalene-1,4-dione (18a): The General Procedure for Synthesis of Amino-alkynes was followed using 5-iodo-1,4-naphthoquinone (**12**, 284 mg, 1.0 mmol) and *N*-propargylamine (128 μL, 110.2 mg, 2.0 mmol). Purification by column chromatography on silica gel (*n*-hexane/EtOAc 8:2) yielded 8-iodo-2-(prop-2-yn-1-ylamino)naphthalene-1,4-dione (**18a**, 243 mg, 72%) as an orange solid. ^{1}**H NMR** (400 MHz, DMSO-d_6) δ = 8.31 (dd, J = 8.0, 1.2 Hz, 1H), 8.05 (dd, J = 7.6, 0.8 Hz, 1H), 7.97 (t, J = 6.0 Hz, 1H), 7.47 (t, J = 7.6 Hz, 1H), 5.79 (s, 1H), 4.05 (dd, J = 6.0, 2.4 Hz, 2H), 3.27 (t, J = 2.4 Hz, 1H). ^{13}**C NMR** (100 MHz, DMSO-d_6) δ = 180.2 (C_q), 180.1 (C_q), 148.9 (C_q), 146.6 (CH), 136.0 (C_q), 135.2 (CH), 130.0 (C_q), 126.8 (CH), 100.9 (CH), 94.6 (C_q), 79.3 (C_q), 75.1 (CH), 31.7 (CH_2). **IR** (KBr): $\tilde{\nu}$ = 3371, 3280, 1673, 1600, 1494, 1250, 660 cm^{-1}. **m.p.** (°C) = 44-49.

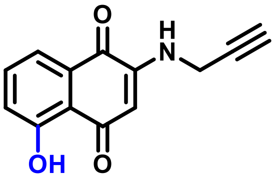

5-hydroxy-2-(prop-2-yn-1-ylamino)naphthalene-1,4-dione (18b): The General Procedure for Synthesis of Amino-alkynes was followed using Juglone (**13**, 174 mg, 1.0 mmol) and *N*-propargylamine (128 μL, 110.2 mg, 2.0 mmol). Purification by column chromatography on silica gel (*n*-hexane/EtOAc 8:2) yielded 8-hydroxy-2-(prop-2-yn-1-ylamino)naphthalene-1,4-dione (**18b**, 80 mg, 35%) as a red solid. ^{1}**H NMR** (400 MHz, DMSO-d_6) δ = 13.19 (s, 1H), 8.20 (br s, 1H), 7.61 (t, J = 7.6 Hz, 1H), 7.52 (d, J = 7.6 Hz, 1H), 7.29 (d, J = 8.4 Hz, 1H), 5.74 (s, 1H), 4.08 (d, J = 3.6 Hz, 2H), 3.29 (s, 1H). ^{13}**C NMR** (100 MHz, DMSO-d_6) δ = 188.2 (C_q), 180.5 (C_q), 160.1 (C_q), 148.9 (C_q), 134.4 (CH), 130.4 (C_q), 125.1 (CH), 118.5 (CH), 114.1 (C_q), 100.0 (CH), 78.4 (C_q), 74.8 (CH), 31.2 (CH_2). **IR** (KBr): $\tilde{\nu}$ = 3348, 3296, 2917, 2358, 2340, 1600, 1616, 1471, 1249, 1225 cm^{-1}. **m.p. (°C)** = 45-51.

The analytical data are in accordance with those reported in the literature [45]. *The structure of the product was also confirmed by X-ray diffraction* (CCDC number = 2226471) *as shown below.*

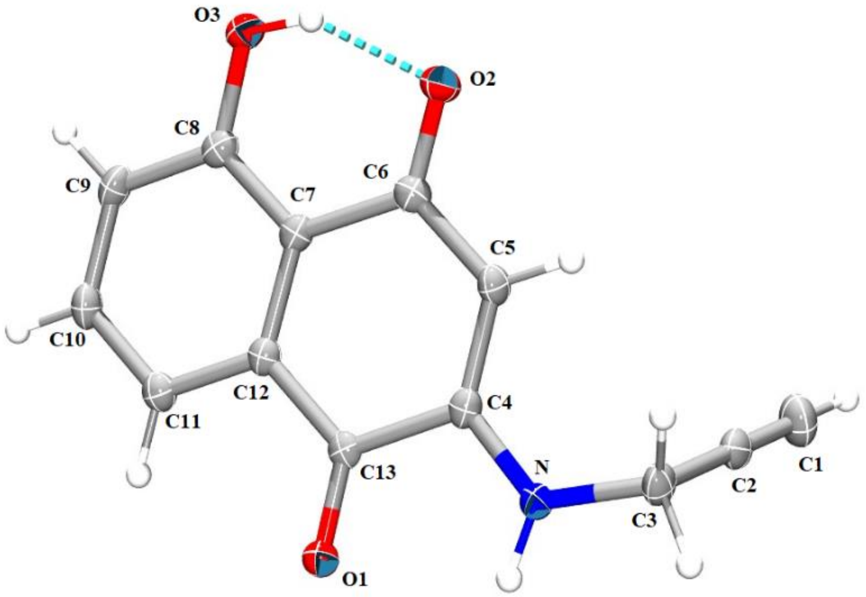

Figure S1. The crystallographically independent molecule in the crystal structure of compound **18b** (ellipsoids for non-hydrogen atoms are at 50% probability level while hydrogens are drawn as arbitrary radius spheres). Distances in Angstroms: O3-H3 0.84(2); H3···O2 1.81(2); O2···O3 2.557(2); angle in degrees: O3-H3···O2 146.4(11).

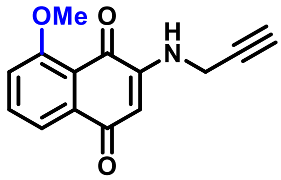

8-methoxy-2-(prop-2-yn-1-ylamino)naphthalene-1,4-dione (18c): The General Procedure for Synthesis of Amino-alkynes was followed using 5-methoxy-1,4-naphthoquinone (**14**, 188 mg, 1.0 mmol) and *N*-propargylamine (128 μL, 110.2 mg, 2.0 mmol). Purification by column chromatography on silica gel (*n*-hexane/EtOAc 8:2) yielded 8-methoxy-2-(prop-2-yn-1-ylamino)naphthalene-1,4-dione (**18c**, 125 mg, 52%) as a red solid. ^{1}**H NMR** (400 MHz, DMSO-d_6) δ = 7.77 (t, J = 8.0 Hz, 1H), 7.66 (t, J = 6.0 Hz, 1H), 7.59 (dd, J = 7.2, 0.4 Hz, 1H), 7.42 (d, J = 8.0 Hz, 1H), 5.70 (s, 1H), 4.04 (dd, J = 6.0, 2.0 Hz, 2H), 3.93 (s, 3H), 3.26 (t, J = 2.3 Hz, 1H). ^{13}**C NMR** (100 MHz, DMSO-d_6) δ = 181.2 (C_q), 179.3 (C_q), 159.7 (C_q), 148.9 (C_q), 136.1 (CH), 135.1 (C_q), 117.9 (CH), 117.8 (C_q), 117.0 (CH), 99.7 (CH), 79.1 (C_q), 74.5 (CH), 56.4 (CH_3), 31.2 (CH_2). **IR** (KBr): $\tilde{\nu}$ = 3339, 3203, 2941, 1674, 1608, 1577, 1261, 1217 cm^{-1}. **m.p.** (°C) = 52-57.

The analytical data are in accordance with those reported in the literature [45]. *The structure of the product was also confirmed by X-ray diffraction* (CCDC number = 2226469) *as shown below*.

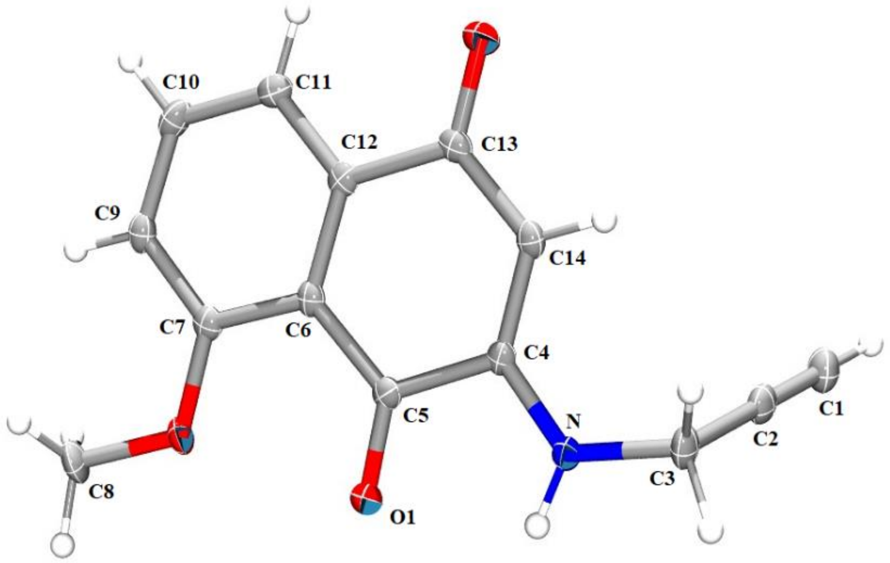

Figure S2. The crystallographically independent molecule in the crystal structure of compound **18c** (ellipsoids for non-hydrogen atoms are at 50% probability level while hydrogens are drawn as arbitrary radius spheres).

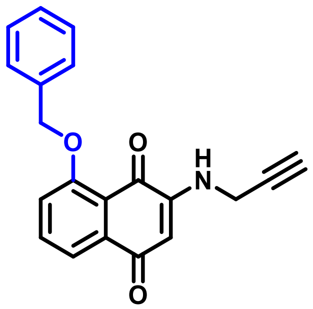

8-benzyloxy-2-(prop-2-yn-1-ylamino)naphthalene-1,4-dione (18d): The General Procedure for Synthesis of Amino-alkynes was followed using 5-benzyloxy-1,4-naphthoquinone (**15**, 264 mg, 1.0 mmol) and *N*-propargylamine (128 µL, 110.2 mg, 2.0 mmol). Purification by column chromatography on silica gel (*n*-hexane/EtOAc 8:2) yielded 8-benzyloxy-2-(prop-2-yn-1-ylamino)naphthalene-1,4-dione (**18d**, 155 mg, 49%) as a brown solid. **^{1}H NMR** (400 MHz, DMSO-d_6) δ = 7.82 (t, J = 6.0 Hz, 1H), 7.76 (t, J = 8.0 Hz, 1H), 7.61-7.59 (m, 3H), 7.50 (d, J = 8.4 Hz, 1H), 7.41 (t, J = 7.2 Hz, 2H), 7.33 (t, J = 7.6 Hz, 1H), 5.69 (s, 1H), 5.30 (s, 2H), 4.01 (dd, J = 5.6, 2.0 Hz, 2H), 3.23 (t, J = 2.0 Hz, 1H). **^{13}C NMR** (100 MHz, DMSO-d_6) δ = 181.3 (C_q), 179.5 (C_q), 158.6 (C_q), 149.0 (C_q), 136.7 (CH), 136.1 (C_q), 135.2 (C_q), 128.4 (CH), 127.7 (CH), 127.0 (CH), 118.2 (CH), 118.2 (CH), 118.2 (C_q), 99.8 (CH), 79.1 (C_q), 74.5 (CH), 70.1 (CH_2), 31.2 (CH_2). **IR** (KBr): $\tilde{\nu}$ = 3359, 3289, 2359, 1674, 1601, 1577, 1494, 1252, cm^{-1}. **m.p.** (°C) = 50-55.

The analytical data are in accordance with those reported in the literature [45]. *The structure of the product was also confirmed by X-ray diffraction* (CCDC number = 2226470) *as shown below*.

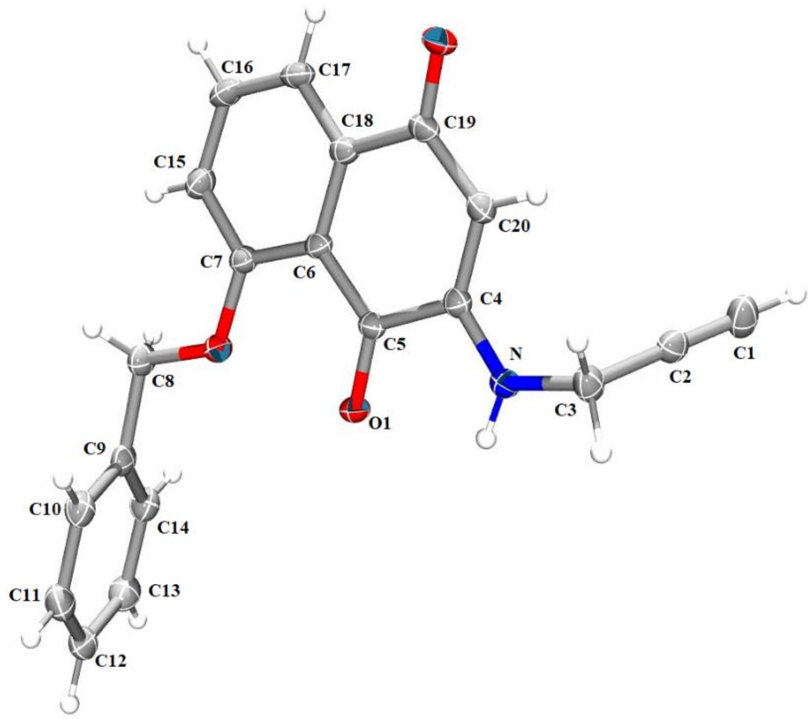

Figure S3. The crystallographically independent molecule in the crystal structure of compound **18d** (ellipsoids for non-hydrogen atoms are at 50% probability level while hydrogens are drawn as arbitrary radius spheres).

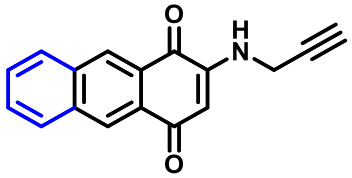

2-(prop-2-yn-1-ylamino)anthracene-1,4-dione (18e): The General Procedure for Synthesis of Amino-alkynes was followed using 1,4-antraquinone (**17**, 208 mg, 1.0 mmol) and *N*-propargylamine (128 μL, 110.2 mg, 2.0 mmol). Purification by column chromatography on silica gel (*n*-hexane/EtOAc 8:2) 2-(prop-2-yn-1-ylamino)anthracene-1,4-dione (**18e**, 167 mg, 64%) as a brown solid. **^{1}H NMR** (400 MHz, DMSO-d_6) δ = 8.62 (s, 1H), 8.50 (s, 1H), 8.21 (dd, J = 13.6, 7.6 Hz, 2H), 7.88 (t, J = 5.6 Hz, 1H), 7.75-7.68 (m, 2H), 5.89 (s, 1H), 4.06 (d, J = 4.0 Hz, 2H), 3.25 (br s, 1H). **^{13}C NMR** (100 MHz, DMSO-d_6) δ = 181.5 (C_q), 181.0 (C_q), 149.0 (C_q), 135.1 (C_q), 133.7 (C_q), 130.3 (CH), 130.0 (CH), 129.9 (CH), 129.3 (C_q), 129.1 (CH), 128.7 (CH), 127.5 (C_q), 126.9 (CH), 103.4 (CH), 79.1 (C_q), 74.7 (CH), 31.3 (CH_2). **IR** (KBr): $\tilde{\nu}$ = 3356, 3217, 2922, 2359, 1668, 1598, 1509, 1320, 1263 cm^{-1}. **m.p. (°C) =** 42-47.

General procedure for the Triazole Synthesis via a Copper-Catalysed 1,3-Dipolar Cycloaddition (19a-21e)

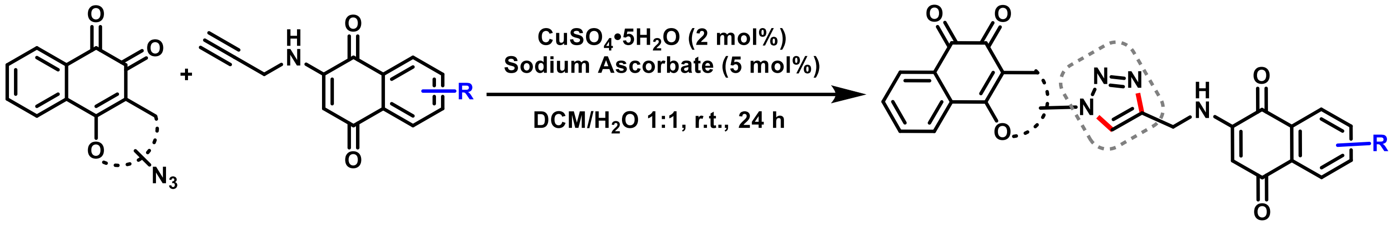

A reaction tube was charged with the corresponding azide-lapachone derivative (0.2 mmol, 1.0 equiv.), aminoalkyne naphthoquinone (0.22 mmol, 1.1 equiv.), $CuSO_4{\cdot}5H_2O$ (1 mg, 0.002 mmol, 1 mol %), and sodium ascorbate (4 mg, 0.01 mmol, 5 mol %). Then, a mixture DCM/distilled water 1:1 (4 mL) was added. The reaction mixture was kept under vigorous stirring at room temperature for 24 h. The crude product was partitioned with distilled water (20 mL) and DCM (3 x 30 mL). The organic phase was dried over Na_2SO_4, filtered, and purified by column chromatography (*n*-hexane/EtOAc 7:3).

Characterization Data of Products 19a-21e

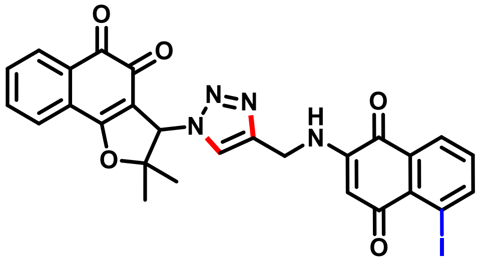

3-(4-(((5-iodo-1,4-dioxo-1,4-dihydronaphthalen-2-yl)amino)methyl)-1*H*-1,2,3-triazol-1-yl)-2,2-dimethyl-2,3-dihydronaphtho[1,2-*b*]furan-4,5-dione (19a): The general procedure for the triazole synthesis was followed by using 3-azido-2,2-dimethyl-2,3-dihydronaphtho[1,2-*b*]furan-4,5-dione (**4**) (54 mg, 0.2 mmol) and 5-iodo-2-(prop-2-yn-1-ylamino)naphthalene-1,4-dione (**18a**) (74 mg, 0.22 mmol) as starting material. Purification by column chromatography on silica gel (*n*-hexane/EtOAc 4:1) yielded **19a** (74 mg, 61%) as an orange solid. **^{1}H NMR** (600 MHz, DMSO-*d*$_6$) δ = 8.29 (s, 1H), 8.04 (d, *J* = 7.2 Hz, 1H), 8.00-7.90 (m, 2H), 7.84-7.78 (m, 3H), 7.75 (d, *J* = 7.2 Hz, 1H), 7.72 (td, 7.2, 0.6 Hz, 1H), 6.04 (s, 1H), 5.70 (d, *J* = 6.0 Hz, 1H), 4.48 (d, *J* = 6.0 Hz, 2H), 1.68 (s, 3H), 1.00 (s, 3H). **^{13}C NMR** (150 MHz, DMSO-*d*$_6$) δ = 181.7 (C_q), 180.0 (C_q), 179.6 (C_q), 174.7 (C_q), 170.0 (C_q), 148.9 (C_q), 148.4 (C_q), 146.3 (CH), 143.2 (C_q), 135.1 (C_q), 134.9 (CH), 133.2 (CH), 128.9 (CH), 125.5 (CH), 125.2 (CH), 123.6 (CH), 111.3 (C_q), 100.7 (CH), 99.8 (C_q), 95.5 (C_q), 94.3 (C_q), 66.2 (CH), 37.8 (CH_2), 37.7 (C_q), 27.1 (CH_3), 22.7 (CH), 20.7 (CH_3). **IR** (KBr): $\tilde{\nu}$ = 3365, 2360, 1657, 1609, 1570, 1508, 1221, 1082, 662 cm^{-1}. **m.p.** (°C) = 112-116. **HRMS** (ESI): Calcd. for $C_{27}H_{20}IN_4O_5$ $[M+H]^+$ 607.0478, found 607.0459.

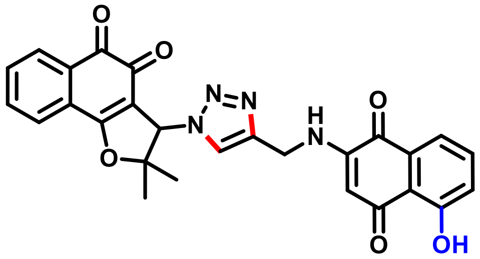

3-(4-(((5-hydroxy-1,4-dioxo-1,4-dihydronaphthalen-2-yl)amino)methyl)-1*H*-1,2,3-triazol-1-yl)-2,2-dimethyl-2,3-dihydronaphtho[1,2-*b*]furan-4,5-dione (19b): The general procedure for the triazole synthesis was followed by using 3-azido-2,2-dimethyl-2,3-dihydronaphtho[1,2-*b*]furan-4,5-dione (**4**) (54 mg, 0.2 mmol) and 5-hydroxy-2-(prop-2-yn-1-ylamino)naphthalene-1,4-dione (**18b**) (50 mg, 0.22 mmol) as starting material. Purification by column chromatography on silica gel (*n*-hexane/EtOAc 4:1) yielded **19b** (71 mg, 71%) as an orange solid. **^{1}H NMR** (600 MHz, DMSO-d_6) δ = 13.24 (s, 1H), 8.34 (t, J = 6.0 Hz, 1H), 8.28 (s, 1H), 8.05 (d, J = 7.2 Hz, 1H), 7.84 (t, J = 7.8 Hz, 1H), 7.80 (t, J = 7.8 Hz, 1H), 7.76 (d, J = 7.2 Hz, 1H), 7.58 (t, J = 7.8 Hz, 1H), 7.50 (d, J = 7.8 Hz, 1H), 7.27 (d, J = 8.4 Hz, 1H), 6.03 (s, 1H), 5.68 (s, 1H), 4.51 (d, J = 6.0 Hz, 2H), 1.68 (s, 3H), 1.00 (s, 3H). **^{13}C NMR** (150 MHz, DMSO-d_6) δ = 188.2 (C_q), 180.8 (C_q), 179.9 (C_q), 174.6 (C_q), 169.9 (C_q), 160.3 (C_q), 149.5 (C_q), 142.8 (C_q), 134.9 (CH), 134.5 (CH), 133.2 (CH), 131.8 (C_q), 130.6 (C_q), 128.9 (CH), 126.6 (C_q), 125.3 (CH), 125.1 (CH), 123.7 (CH), 118.7 (CH), 114.4 (C_q), 111.3 (C_q), 99.4 (CH), 95.4 (C_q), 66.2 (CH), 40.1 (C_q), 37.8 (CH_2), 27.0 (CH_3), 20.7 (CH_3). **IR** (KBr): $\tilde{\nu}$ = 3348, 2358, 2340, 1652, 1616, 1569, 1469, 1248, 1051 cm^{-1}. **m.p.** (°C) = 114-118. **HRMS** (ESI): Calcd. for $C_{27}H_{20}N_4O_6Na$ [M+Na]$^+$ 519.1281, found 519.1282.

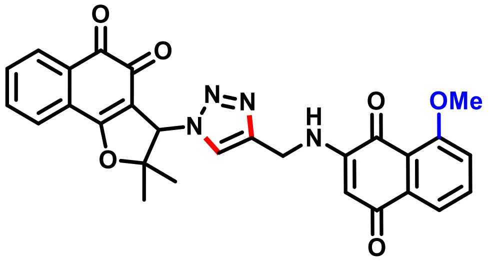

3-(4-(((8-methoxy-1,4-dioxo-1,4-dihydronaphthalen-2-yl)amino)methyl)-1*H*-1,2,3-triazol-1-yl)-2,2-dimethyl-2,3-dihydronaphtho[1,2-*b*]furan-4,5-dione (19c): The general procedure for the triazole synthesis was followed by using 3-azido-2,2-dimethyl-2,3-dihydronaphtho[1,2-*b*]furan-4,5-dione (**4**) (54 mg, 0.2 mmol) and 8-methoxy-2-(prop-2-yn-1-ylamino)naphthalene-1,4-dione (**18c**) (53 mg, 0.22 mmol) as starting material. Purification by column chromatography on silica gel (*n*-hexane/EtOAc 4:1) yielded **19c** (59 mg, 58%) as an orange solid. **^{1}H NMR** (600 MHz, DMSO-d_6) δ = 8.27 (s, 1H), 8.03 (d, J = 7.2 Hz, 1H), 7.83 (t, J = 7.2 Hz, 1H), 7.78 (t, J = 7.8 Hz, 1H), 7.75-7.72 (m, 3H), 7.54 (d, J = 7.2 Hz, 1H), 7.38 (d, J = 8.4 Hz, 1H), 6.03 (s, 1H), 5.59 (s, 1H), 4.45 (d, J = 6.0 Hz, 2H), 3.88 (s, 3H), 1.67 (s, 3H), 0.99 (s, 3H). **^{13}C NMR** (150 MHz, DMSO-d_6) δ = 181.1 (C_q), 179.9 (C_q), 179.4 (C_q), 174.6 (C_q), 169.9 (C_q), 159.7 (C_q), 149.2 (C_q), 136.2 (CH), 135.2 (C_q), 134.8 (CH), 133.1 (CH), 131.8 (C_q), 128.8 (CH), 126.6 (C_q), 125.1 (CH), 123.5 (CH), 117.9 (CH), 117.8 (C_q), 117.0 (CH), 111.3 (C_q), 99.0 (CH), 95.4 (C_q), 66.2 (CH), 56.4 (CH_3), 40.1 (C_q), 37.6 (CH_2), 27.0 (CH_3), 20.7 (CH_3). **IR** (KBr): $\tilde{\nu}$ = 3437, 3238, 2360, 2341, 1667, 1653, 1615, 1572, 1384, 1220, 1048 cm^{-1}. **m.p.** (°C) = 173-179. **HRMS** (ESI): Calcd. for $C_{28}H_{22}N_4O_6Na$ [M+Na]$^+$ 533.1437, found 533.1425.

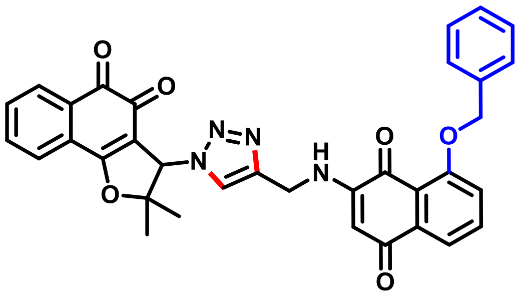

3-(4-(((8-benzyloxy-1,4-dioxo-1,4-dihydronaphthalen-2-yl)amino)methyl)-1*H*-1,2,3-triazol-1-yl)-2,2-dimethyl-2,3-dihydronaphtho[1,2-*b*]furan-4,5-dione (19d): The general procedure for the triazole synthesis was followed by using 3-azido-2,2-dimethyl-2,3-dihydronaphtho[1,2-*b*]furan-4,5-dione (**4**) (54 mg, 0.2 mmol) and 8-benzyloxy-2-(prop-2-yn-1-ylamino)naphthalene-1,4-dione (**18d**) (70 mg, 0.22 mmol) as starting material. Purification by column chromatography on silica gel (*n*-hexane/EtOAc 4:1) yielded **19d** (57 mg, 49%) as an orange solid. ^{1}**H NMR** (600 MHz, DMSO-d_6) δ = 8.25 (s, 1H), 8.04 (d, J = 7.2 Hz, 1H), 7.92 (t, J = 6.0 Hz, 1H), 7.85-7.74 (m, 4H), 7.60 (d, J = 7.2 Hz, 2H), 7.57 (d, J = 7.8 Hz, 1H), 7.50 (d, J = 8.4 Hz, 1H), 7.41 (t, J = 7.2 Hz, 2H), 7.33 (t, J = 7.2 Hz, 1H), 6.02 (s, 1H), 5.62 (s, 1H), 5.30 (s, 2H), 4.44 (d, J = 5.4 Hz, 2H), 1.67 (s, 3H), 0.98 (s, 3H). ^{13}**C NMR** (150 MHz, DMSO-d_6) δ = 181.0 (C$_q$), 179.8 (C$_q$), 179.6 (C$_q$), 174.6 (C$_q$), 169.9 (C$_q$), 158.6 (C$_q$), 149.2 (C$_q$), 143.2 (C$_q$), 136.7 (C$_q$), 136.1 (CH), 135.3 (C$_q$), 134.8 (CH), 133.1 (CH), 131.8 (C$_q$), 128.8 (CH), 128.4 (CH), 127.7 (CH), 127.0 (CH), 126.6 (C$_q$), 125.1 (CH), 123.5 (CH), 118.2 (CH), 118.1 (CH), 111.3 (C$_q$), 99.0 (CH), 95.3 (C$_q$), 70.1 (CH$_2$), 66.1 (CH), 40.1 (C$_q$), 37.6 (CH$_2$), 27.0 (CH$_3$), 20.6 (CH$_3$). **IR** (KBr): $\tilde{\nu}$ = 3372, 2362, 1651, 1609, 1571, 1497, 1356, 1279, 1053 cm^{-1}. **m.p.** (°C) = 205-210. **HRMS** (ESI): Calcd. for $C_{34}H_{27}N_4O_6$ [M+H]$^+$ 587.1931, found 587.1929.

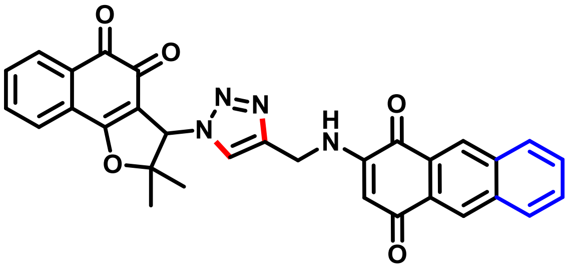

3-(4-(((1,4-dioxo-1,4-dihydroanthracen-2-yl)amino)methyl)-1*H*-1,2,3-triazol-1-yl)-2,2-dimethyl-2,3-dihydronaphtho[1,2-*b*]furan-4,5-dione (19e): The general procedure for the triazole synthesis was followed by using 3-azido-2,2-dimethyl-2,3-dihydronaphtho[1,2-*b*]furan-4,5-dione (**4**) (54 mg, 0.2 mmol) and 2-(prop-2-yn-1-ylamino)anthracene-1,4-dione (**18e**) (58 mg, 0.22 mmol) as starting material. Purification by column chromatography on silica gel (*n*-hexane/EtOAc 4:1) yielded **19e** (45 mg, 42%) as an orange solid. ^{1}**H NMR** (600 MHz, DMSO-d_6) δ = 8.60 (br s, 1H), 8.45 (br s, 1H), 8.31 (br s, 1H), 8.21 (d, J = 7.6 Hz, 1H), 8.17 (d, J = 7.6 Hz, 1H), 8.02 (d, J = 7.1 Hz, 1H), 7.99 (t, J = 4.9 Hz, 1H), 7.81-7.76 (m, 2H), 7.73-7.68 (m, 3H), 6.03 (s, 1H), 5.82 (s, 1H), 4.50 (d, J = 5.3 Hz, 2H), 1.67 (s, 3H), 1.00 (s, 3H). ^{13}**C NMR** (100 MHz, DMSO-d_6) δ = 181.5 (C$_q$), 180.2 (C$_q$), 175.0 (C$_q$), 170.2 (C$_q$), 149.6 (C$_q$), 143.5 (C$_q$), 135.5 (C$_q$), 135.2 (CH), 135.2 (C$_q$), 134.0 (C$_q$), 133.5 (CH), 132.2 (C$_q$), 130.6 (CH), 130.2 (CH), 129.8 (C$_q$), 129.3 (CH), 129.2 (CH), 128.9 (CH), 127.9 (C$_q$), 127.1 (CH), 127.0 (CH), 125.5 (CH), 124.0 (CH), 111.7 (C$_q$), 103.0 (CH), 95.7 (C$_q$), 66.6 (CH), 60.3 (C$_q$), 38.1 (CH$_2$), 27.4 (CH$_3$), 21.1 (CH$_3$). **IR** (KBr): $\tilde{\nu}$ = 3374, 2923, 2852, 2358, 1658, 1601, 1572, 1313, 1261, 1084 cm^{-1}. **m.p.** (°C) **=** 135-139. **HRMS** (ESI): Calcd. for $C_{31}H_{23}N_4O_5$ [M+H]$^+$ 531.1668, found 531.1660.

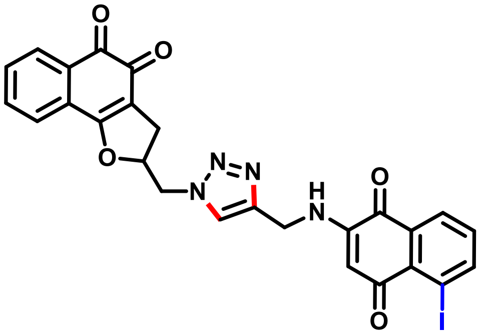

2-((4-(((5-iodo-1,4-dioxo-1,4-dihydronaphthalen-2-yl)amino)methyl)-1*H*-1,2,3-triazol-1-yl)methyl)-2,3-dihydronaphtho[1,2-*b*]furan-4,5-dione (20a): The general procedure for the triazole synthesis was followed by using 2-(azidomethyl)-2,3-dihydronaphtho[1,2-*b*]furan-4,5-dione (**7**) (51 mg, 0.2 mmol) and 5-iodo-2-(prop-2-yn-1-ylamino)naphthalene-1,4-dione (**18a**) (74 mg, 0.22 mmol) as starting material. Purification by column chromatography on silica gel (*n*-hexane/EtOAc 4:1) yielded **20a** (73 mg, 62%) as an orange solid. **^{1}H NMR** (600 MHz, DMSO-d_6) δ = 8.33 (d, J = 2.8 Hz, 1H), 8.28 (d, J = 7.8 Hz, 1H), 8.10-8.08 (m, 1H), 7.99-7.88 (m, 2H), 7.83-7.75 (m, 1H), 7.61 (dd, J = 15.5, 7.2 Hz, 1H), 7.55-7.52 (m, 1H), 7.44-7.43 (m, 1H), 5.69-5.68 (m, 1H), 5.55-5.50 (m, 1H), 4.86 (dd, J = 14.8, 3.0 Hz, 1H), 4.76 (dd, J = 14.8, 7.3 Hz, 1H), 4.51-4.43 (m, 2H), 3.14 (dd, J = 15.4, 10.3 Hz, 1H), 2.80 (dd, J = 15.5, 6.6 Hz, 1H). **^{13}C NMR** (150 MHz, DMSO-d_6) δ = 180.3 (C_q), 179.8 (C_q), 179.5 (C_q), 174.9 (C_q), 168.1 (C_q), 148.8 (C_q), 146.3 (CH), 143.4 (C_q), 138.8 (CH), 135.0 (CH), 132.1 (CH), 129.5 (C_q), 128.7 (CH), 126.8 (C_q), 126.6 (CH), 124.4 (C_q), 124.2 (CH), 115.0 (C_q), 99.6 (CH), 94.2 (C_q), 84.8 (CH), 79.3 (C_q), 69.9 (CH_2), 52.6 (CH_2), 37.6 (CH_2), 29.0 (CH). **IR** (KBr): $\tilde{\nu}$ = 3278, 2360, 1676, 1654, 1607, 1570, 1384, 1241, 658 cm^{-1}. **m.p.** (°C) **=** 150-155. **HRMS** (ESI): Calcd. for $C_{26}H_{18}IN_4O_5$ [M+H]$^+$ 593.0322, found 593.0320.

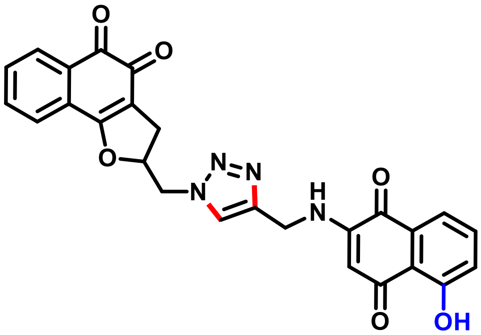

2-((4-(((5-hydroxy-1,4-dioxo-1,4-dihydronaphthalen-2-yl)amino)methyl)-1*H*-1,2,3-triazol-1-yl)methyl)-2,3-dihydronaphtho[1,2-*b*]furan-4,5-dione (20b): The general procedure for the triazole synthesis was followed by using 2-(azidomethyl)-2,3-dihydronaphtho[1,2-*b*]furan-4,5-dione (**7**) (51 mg, 0.2 mmol) and 5-hydroxy-2-(prop-2-yn-1-ylamino)naphthalene-1,4-dione (**18b**) (50 mg, 0.22 mmol) as starting material. Purification by column chromatography on silica gel (*n*-hexane/EtOAc 4:1) yielded **20b** (63 mg, 65%) as an orange solid. **^{1}H NMR** (600 MHz, DMSO-d_6) δ = 13.19 (s, 1H), 8.33 (t, J = 6.0 Hz, 1H), 8.09 (s, 1H), 7.76 (d, J = 7.8 Hz, 1H), 7.59 (dd, J = 17.4, 7.8 Hz, 2H), 7.52 (t, J = 7.2 Hz, 1H), 7.47 (dd, J = 13.2, 7.8 Hz, 2H), 7.27 (d, J = 7.8 Hz, 1H), 5.62 (s, 1H), 5.54 (d, J = 6.0 Hz, 1H), 4.86 (dd, J = 15.0, 2.4 Hz, 1H), 4.77 (dd, J = 15.0, 7.2 Hz, 1H), 4.53-4.45 (m, 2H), 3.15 (dd, J = 15.6, 10.2 Hz, 1H), 2.81 (dd, J = 15.6, 6.6 Hz, 1H). **^{13}C NMR** (150 MHz, DMSO-d_6) δ = 188.1 (C_q), 180.7 (C_q), 180.3 (C_q), 174.9 (C_q), 168.0 (C_q), 160.3 (C_q), 149.4 (C_q), 134.7 (CH), 134.5 (CH), 132.1 (CH), 130.5 (C_q), 130.4 (C_q), 128.7 (CH), 126.8 (C_q), 125.3 (CH), 124.4 (CH), 124.1 (CH), 118.7 (CH), 115.0 (C_q), 114.4 (C_q), 99.1 (CH), 84.7 (CH), 52.6 (CH_2), 40.1 (C_q), 37.6 (CH_2), 28.9 (CH_2). **IR** (KBr): $\tilde{\nu}$ = 3383, 2361, 1681, 1616, 1572, 1469, 1384, 1255,

1230, cm^{-1}. **m.p.** (°C) = 150-155. **HRMS** (ESI): Calcd. for $C_{26}H_{19}N_4O_6$ [M+H]$^+$ 483.1305, found 483.1302.

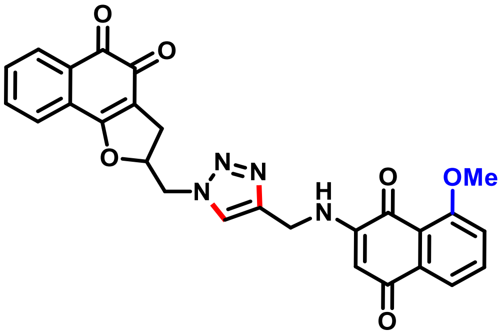

2-((4-(((8-methoxy-1,4-dioxo-1,4-dihydronaphthalen-2-yl)amino)methyl)-1*H*-1,2,3-triazol-1-yl)methyl)-2,3-dihydronaphtho[1,2-*b*]furan-4,5-dione (20c): The general procedure for the triazole synthesis was followed by using 2-(azidomethyl)-2,3-dihydronaphtho[1,2-*b*]furan-4,5-dione (**7**) (51 mg, 0.2 mmol) and 8-methoxy-2-(prop-2-yn-1-ylamino)naphthalene-1,4-dione (**18c**) (53 mg, 0.22 mmol) as starting material. Purification by column chromatography on silica gel (*n*-hexane/EtOAc 4:1) yielded **20c** (85 mg, 86%) as an orange solid. ^{1}**H NMR** (600 MHz, DMSO-d_6) δ = 8.67 (br s, 1H), 8.50 (br s, 1H), 8.26-8.25 (m, 1H), 8.21-8.19 (m, 1H), 8.06 (br s, 1H), 7.92-7.91 (m, 1H), 7.77-7.73 (m, 4H), 7.61 (br s, 1H), 5.89 (s, 1H), 4.65 (d, J = 11.8 Hz, 1H), 4.55 (br s, 2H), 4.31 (t, J = 10.3 Hz, 1H), 3.51 (s, 3H), 2.20 (d, J = 15.9 Hz, 1H), 2.09-2.05 (m, 1H). ^{13}**C NMR** (150 MHz, DMSO-d_6) δ = 181.1 (C_q), 180.3 (C_q), 179.4 (C_q), 174.9 (C_q), 168.0 (C_q), 159.7 (C_q), 149.2 (C_q), 143.5 (C_q), 136.2 (CH), 135.3 (C_q), 134.8 (CH), 132.1 (CH), 130.4 (C_q), 128.7 (CH), 126.8 (C_q), 124.3 (CH), 124.2 (CH), 118.0 (CH), 117.0 (CH), 114.9 (C_q), 98.8 (CH), 84.8 (CH), 56.5 (CH_3), 52.6 (CH_2), 40.1 (C_q), 37.5 (CH_2), 29.0 (CH_2). **IR** (KBr): $\tilde{\nu}$ = 3430, 1612, 1572, 1384, 1284 cm^{-1}.**m.p.** (°C) = 183-189. **HRMS** (ESI): Calcd. for $C_{27}H_{21}N_4O_6$ [M+H]$^+$ 497.1461, found 497.1444.

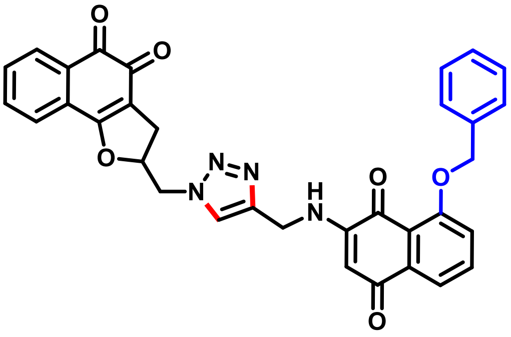

2-((4-(((8-benzyloxy-1,4-dioxo-1,4-dihydronaphthalen-2-yl)amino)methyl)-1*H*-1,2,3-triazol-1-yl)methyl)-2,3-dihydronaphtho[1,2-*b*]furan-4,5-dione (20d): The general procedure for the triazole synthesis was followed by using 2-(azidomethyl)-2,3-dihydronaphtho[1,2-*b*]furan-4,5-dione (**7**) (51 mg, 0.2 mmol) and 8-benzyloxy-2-(prop-2-yn-1-ylamino)naphthalene-1,4-dione (**18d**) (70 mg, 0.22 mmol) as starting material. Purification by column chromatography on silica gel (*n*-hexane/EtOAc 4:1) yielded **20d** (81 mg, 71%) as an orange solid. ^{1}**H NMR** (600 MHz, DMSO-d_6) δ = 8.08 (s, 1H), 7.96 (t, J = 6.0 Hz, 1H), 7.79 (d, J = 7.2 Hz, 1H), 7.76 (t, J = 8.4 Hz, 1H), 7.62 (d, J = 7.8 Hz, 2H), 7.59 (dd, J = 7.2, 0.6 Hz, 1H), 7.56 (d, J = 7.2 Hz, 1H), 7.52 (t, J = 6.6 Hz, 2H), 7.42 (t, J = 6.0 Hz, 3H), 7.34 (t, J = 7.8 Hz, 1H), 5.62 (s, 1H), 5.54-5.50 (m, 1H), 5.32-5.30 (m, 2H), 4.85 (dd, J = 15.0, 3.6 Hz, 1H), 4.76 (dd, J = 15.6, 7.8 Hz, 1H), 4.49-4.43 (m, 2H), 3.15 (dd, J = 15.0, 10.2 Hz, 1H), 2.80 (dd, J = 15.6, 6.6 Hz, 1H). ^{13}**C NMR** (150 MHz, DMSO-d_6) δ = 181.1 (C_q), 180.3 (C_q), 179.5 (C_q), 174.9 (C_q), 168.0 (C_q), 158.6 (C_q), 143.5 (C_q), 136.8 (C_q), 136.2 (CH), 135.3

(C_q), 134.7 (CH), 132.1 (CH), 130.4 (C_q), 128.7 (CH), 128.5 (CH), 127.8 (CH), 127.0 (CH), 126.8 (C_q), 124.3 (CH), 124.1 (CH), 118.3 (CH), 118.2 (C_q), 118.1 (CH), 115.0 (C_q), 98.9 (CH), 84.8 (CH), 70.2 (CH_2), 52.6 (CH_2), 40.1 (C_q), 37.5 (CH_2), 29.0 (CH_2). **IR** (KBr): $\tilde{\nu}$ = 3432, 1649, 1613, 1572, 1384, 1290, 1048 cm^{-1}. **m.p.** (°C) = 115-120. **HRMS** (ESI): Calcd. for $C_{33}H_{25}N_4O_6$ $[M+H]^+$ 573.1774, found 573.1764.

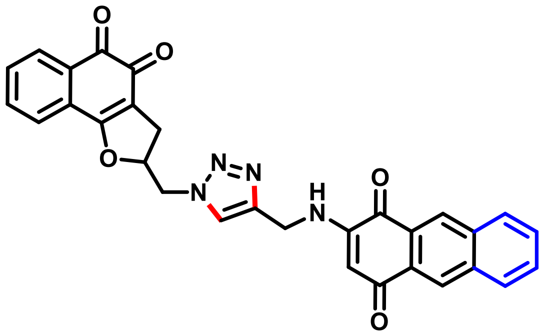

2-((4-(((1,4-dioxo-1,4-dihydroanthracen-2-yl)amino)methyl)-1*H*-1,2,3-triazol-1-yl)methyl)-2,3-dihydronaphtho[1,2-*b*]furan-4,5-dione (20e): The general procedure for the triazole synthesis was followed by using 2-(azidomethyl)-2,3-dihydronaphtho[1,2-*b*]furan-4,5-dione (**7**) (51 mg, 0.2 mmol) and 2-(prop-2-yn-1-ylamino)anthracene-1,4-dione (**18e**) (58 mg, 0.22 mmol) as starting material. Purification by column chromatography on silica gel (*n*-hexane/EtOAc 4:1) yielded **20e** (65 mg, 63%) as an orange solid. **^{1}H NMR** (600 MHz, DMSO-d_6) δ = 8.62 (t, J = 3.0 Hz, 1H), 8.47 (t, J = 2.4 Hz, 1H), 8.25 (d, J = 7.8 Hz, 1H), 8.20 (d, J = 7.2 Hz, 1H), 8.13 (s, 1H), 8.02 (t, J = 6.0 Hz, 1H), 7.78-7.71 (m, 3H), 7.64-7.63 (m, 1H), 7.52-7.49 (m, 1H), 7.47-7.45 (m, 1H), 5.85 (t, J = 1.8 Hz, 1H), 5.56-5.51 (m, 1H), 4.86 (dd, J = 15.0, 3.6 Hz, 1H), 4.77 (dd, J = 14.4, 7.2 Hz, 1H), 4.55-4.48 (m, 2H), 3.15 (dd, J = 15.6, 10.8 Hz, 1H), 2.81 (dd, J = 15.6, 6.6 Hz, 1H). **^{13}C NMR** (150 MHz, DMSO-d_6) δ = 181.1 (C_q), 181.0 (C_q), 180.2 (C_q), 174.8 (C_q), 167.9 (C_q), 149.1 (C_q), 143.4 (C_q), 135.1 (C_q), 134.7 (CH), 133.6 (C_q), 132.0 (CH), 130.2 (CH), 129.8 (CH), 129.8 (CH), 129.4 (C_q), 128.9 (CH), 128.6 (CH), 128.5 (CH), 127.5 (C_q), 126.7 (CH), 124.2 (CH), 124.1 (CH), 114.9 (C_q), 102.4 (CH), 84.7 (CH), 54.9 (C_q), 52.5 (CH_2), 40.1 (C_q), 37.4 (CH_2), 29.0 (CH_2). **IR** (KBr): $\tilde{\nu}$ = 3438, 2353, 1599, 1573, 1384, 1318, 1266 cm^{-1}. **m.p.** (°C) = 158-163. **HRMS** (ESI): Calcd. for $C_{30}H_{21}N_4O_5$ $[M+H]^+$ 517.1512, found 517.1499.

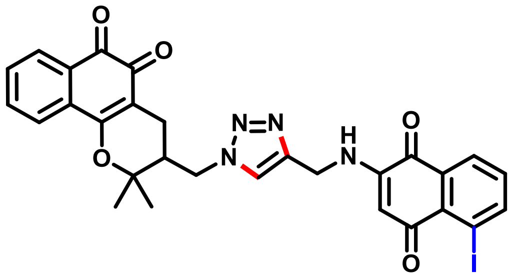

3-((4-(((5-iodo-1,4-dioxo-1,4-dihydronaphthalen-2-yl)amino)methyl)-1*H*-1,2,3-triazol-1-yl)methyl)-2,2-dimethyl-3,4-dihydro-2*H*-benzo[*h*]chromene-5,6-dione (21a): The general procedure for the triazole synthesis was followed by using 3-(azidomethyl)-2,2-dimethyl-3,4-dihydro-2*H*-benzo[*h*]chromene-5,6-dione (**10**) (60 mg, 0.2 mmol) and 5-iodo-2-(prop-2-yn-1-ylamino)naphthalene-1,4-dione (**18a**) (74 mg, 0.22 mmol) as starting material. Purification by column chromatography on silica gel (*n*-hexane/EtOAc 4:1) yielded **21a** (81 mg, 64%) as an orange solid. **^{1}H NMR** (600 MHz, DMSO-d_6) δ = 8.25 (d, J = 7.8 MHz, 1H), 8.15 (s, 1H), 8.08 (t, J = 6.0 MHz, 1H), 7.97 (d, J = 7.8 Hz, 1H), 7.86 (d, 7.2 Hz, 1H), 7.72 (d, J = 3.6 Hz, 2H), 7.58-7.55 (m, 1H), 7.41 (t, J = 7.8 Hz, 1H), 5.73 (s, 1H), 4.62 (dd, J = 13.8, 4.2 Hz, 1H), 4.49 (d, J = 6.0 Hz, 2H), 4.28 (dd, J = 13.8, 10.2 Hz, 1H), 2.46-2.41

(m, 1H), 2.17 (dd, 17.4, 5.4 Hz, 1H), 2.05 (dd, *J* = 17.4, 9.0 Hz, 1H), 1.50 (s, 3H), 1.34 (s, 3H). **^{13}C NMR** (150 MHz, DMSO-*d*$_6$) δ = 179.9 (C$_q$), 179.6 (C$_q$), 178.9 (C$_q$), 177.8 (C$_q$), 160.2 (C$_q$), 148.8 (C$_q$), 146.2 (CH), 143.5 (C$_q$), 135.9 (C$_q$), 135.1 (CH), 134.9 (CH), 131.8 (C$_q$), 131.1 (CH), 130.0 (C$_q$), 129.6 (C$_q$), 128.0 (CH), 126.5 (CH), 123.9 (CH), 123.9 (CH), 111.6 (C$_q$), 99.8 (CH), 94.2 (C$_q$), 80.7 (C$_q$), 50.2 (CH$_2$), 40.4 (CH), 37.7 (CH$_2$), 26.6 (CH$_3$), 21.4 (CH$_3$), 19.9 (CH$_2$). **IR** (KBr): $\tilde{\nu}$ = 3436, 2355, 2339, 1606, 1573, 1231, 729 cm^{-1}. **m.p.** (°C) **=** 146-148. **HRMS** (ESI): Calcd. for $C_{29}H_{24}IN_4O_5$ [M+H]$^+$ 635.0791, found 635.0792.

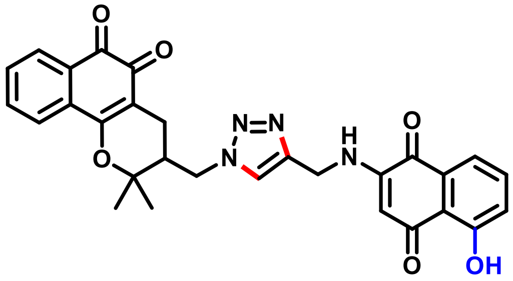

3-((4-(((5-hydroxy-1,4-dioxo-1,4-dihydronaphthalen-2-yl)amino)methyl)-1*H*-1,2,3-triazol-1-yl)methyl)-2,2-dimethyl-3,4-dihydro-2*H*-benzo[*h*]chromene-5,6-dione (21b): The general procedure for the triazole synthesis was followed by using 3-(azidomethyl)-2,2-dimethyl-3,4-dihydro-2*H*-benzo[*h*]chromene-5,6-dione (**10**) (60 mg, 0.2 mmol) and 5-hydroxy-2-(prop-2-yn-1-ylamino)naphthalene-1,4-dione (**18b**) (50 mg, 0.22 mmol) as starting material. Purification by column chromatography on silica gel (*n*-hexane/EtOAc 4:1) yielded **21b** (60 mg, 57%) as an orange solid. **^{1}H NMR** (600 MHz, DMSO-*d*$_6$) δ = 13.24 (d, *J* = 3.0 Hz, 1H), 8.37 (dd, *J* = 6.0, 4.2 Hz, 1H), 8.17 (d, *J* = 3.0 Hz, 1H), 7.89 (dd, *J* = 7.8, 4.2 Hz, 1H), 7.75 (t, *J* = 3.0 Hz, 2H), 7.60-7.54 (m, 2H), 7.50-7.48 (m, 1H), 7.25-7.23 (m, 1H), 5.71 (d, *J* = 1.8 Hz, 1H), 4.64 (dd, *J* = 13.8, 4.2 Hz, 1H), 4.53 (d, *J* = 6.6 Hz, 2H), 4.29 (dd, *J* = 13.2, 11.4 Hz, 1H), 2.48-2.43 (m, 1H), 2.18 (dd, *J* = 17.4, 5.4 Hz, 1H), 2.05 (dd, *J* = 17.4, 9.6 Hz, 1H), 1.51 (s, 3H), 1.36 (s, 3H). **^{13}C NMR** (150 MHz, DMSO-*d*$_6$) δ = 188.2 (C$_q$), 180.8 (C$_q$), 178.9 (C$_q$), 177.7 (C$_q$), 160.3 (C$_q$), 160.1 (C$_q$), 149.5 (C$_q$), 143.1 (C$_q$), 135.0 (CH), 134.4 (CH), 131.7 (C$_q$), 131.0 (CH), 130.0 (C$_q$), 127.9 (CH), 125.3 (CH), 124.0 (CH), 123.9 (CH), 118.6 (CH), 114.4 (C$_q$), 111.5 (C$_q$), 99.3 (CH), 80.6 (C$_q$), 50.1 (CH$_2$), 40.3 (CH), 37.6 (CH$_2$), 26.6 (CH$_3$), 21.4 (CH$_3$), 19.8 (CH$_2$), 18.9 (C$_q$). **IR** (ATR): $\tilde{\nu}$ = 3376, 2360, 1695, 1680, 1604, 1568, 1473, 1399, 1382, 1365, 1303, 1254, 1235, 1133, 842, 773 cm^{-1}. **m.p.** (°C) **=** 187-189. **HRMS** (ESI): Calcd. for $C_{29}H_{25}N_4O_6$ [M+H]$^+$ 525.1774, found 525.1756.

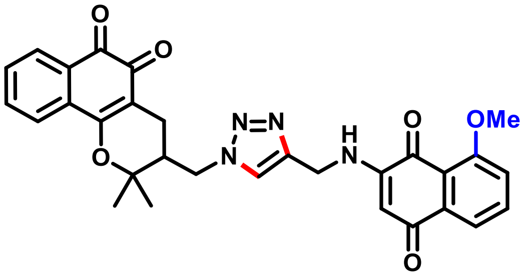

3-((4-(((8-methoxy-1,4-dioxo-1,4-dihydronaphthalen-2-yl)amino)methyl)-1*H*-1,2,3-triazol-1-yl)methyl)-2,2-dimethyl-3,4-dihydro-2*H*-benzo[*h*]chromene-5,6-dione (21c): The general procedure for the triazole synthesis was followed by using 3-(azidomethyl)-2,2-dimethyl-3,4-dihydro-2*H*-benzo[*h*]chromene-5,6-dione (**10**) (60 mg, 0.2 mmol) and 8-methoxy-2-(prop-2-yn-1-ylamino)naphthalene-1,4-dione (**18c**) (53 mg, 0.22 mmol) as starting material. Purification by column chromatography on silica gel (*n*-hexane/EtOAc 4:1) yielded **21c** (42 mg, 39%) as an orange solid. **^{1}H NMR** (600 MHz, DMSO-*d*$_6$) δ = 8.30 (s, 1H), 8.14 (s, 1H), 7.91 (d, *J* = 7.2 Hz, 1H), 7.81 (t, *J* = 6.0 Hz, 1H), 7.77-7.76 (m, 2H), 7.74

(d, J = 7.8 Hz, 1H), 7.62-7.59 (m, 1H), 7.55 (dd, J = 7.8, 0.6 Hz, 1H), 7.40 (d, J = 8.4 Hz, 1H), 5.65 (s, 1H), 4.63 (dd, J = 13.8, 4.8 Hz, 1H), 4.48 (d, J = 6.0 Hz, 2H), 4.28 (dd, J = 13.8, 10.2 Hz. 1H), 3.91 (s, 3H), 2.18 (dd, J = 17.4, 5.4 Hz, 1H), 2.06 (dd, J = 17.4, 9.0 Hz, 1H), 1.51 (s, 3H), 1.36 (s, 3H). **^{13}C NMR** (100 MHz, DMSO-d_6) δ = 181.1 (C_q), 179.4 (C_q), 178.9 (C_q), 177.7 (C_q), 160.1 (C_q), 159.7 (C_q), 149.2 (C_q), 143.5 (C_q), 136.1 (CH), 135.3 (C_q), 135.0 (CH), 131.7 (C_q), 131.0 (CH), 130.0 (C_q), 127.9 (CH), 123.8 (CH), 123.8 (CH), 117.9 (CH), 117.9 (C_q), 116.9 (CH), 111.5 (C_q), 98.9 (CH), 80.6 (C_q), 56.4 (CH_3), 50.0 (CH_2), 40.3 (CH), 37.5 (CH_2), 26.6 (CH_3), 21.4 (CH_3), 19.8 (CH_2) cm^{-1}. **IR** (KBr): $\tilde{\nu}$ = 3354, 2926, 2360, 1695, 1576, 1506, 1286, 1262, 1234, 1049. cm^{-1}. **m.p.** (°C) = 140-142. **HRMS** (ESI): Calcd. for $C_{30}H_{27}N_4O_6$ [M+H]$^+$ 539.1931, found 539.1956.

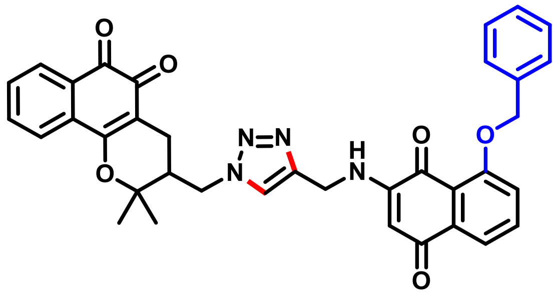

3-((4-(((8-benzyloxy-1,4-dioxo-1,4-dihydronaphthalen-2-yl)amino)methyl)-1*H*-1,2,3-triazol-1-yl)methyl)-2,2-dimethyl-3,4-dihydro-2*H*-benzo[*h*]chromene-5,6-dione (21d): The general procedure for the triazole synthesis was followed by using 3-(azidomethyl)-2,2-dimethyl-3,4-dihydro-2*H*-benzo[*h*]chromene-5,6-dione (**10**) (60 mg, 0.2 mmol) and 8-benzyloxy-2-(prop-2-yn-1-ylamino)naphthalene-1,4-dione (**18d**) (70 mg, 0.22 mmol) as starting material. Purification by column chromatography on silica gel (*n*-hexane/EtOAc 4:1) yielded **21d** (65 mg, 53%) as an orange solid. **^{1}H NMR** (600 MHz, DMSO-d_6) δ = 8.14 (s, 1H), 7.96 (t, J = 6.0 Hz, 1H), 7.89 (d, J = 7.8 Hz, 1H), 7.75-7.72 (m, 3H), 7.61 (d, J = 7.2 Hz, 2H), 7.59-7.58 (m, 1H), 7.56 (d, J = 7.2 Hz, 1H), 7.48 (d, J = 8.4 Hz, 1H), 7.41 (t, J = 7.8 Hz, 2H), 7.32 (t, J = 7.2 Hz, 1H), 5.67 (s, 1H), 5.29 (s, 2H), 4.63 (dd, J = 14.4, 4.8 Hz, 1H), 4.47 (d, J = 6.0, 2H), 4.29 (dd, J = 13.8, 9.6 Hz, 1H), 2.47-2.42 (m, 1H), 2.18 (dd, J = 17.4, 5.4 Hz, 1H), 2.06 (dd, J = 17.4, 9.0 Hz, 1H), 1.50 (s, 3H), 1.35 (s, 3H). **^{13}C NMR** (150 MHz, DMSO-d_6) δ = 181.0 (C_q), 179.6 (C_q), 178.9 (C_q), 177.7 (C_q), 160.1 (C_q), 158.5 (C_q), 143.5 (C_q), 136.7 (C_q), 135.4 (C_q), 135.0 (CH), 131.7 (C_q), 131.0 (CH), 130.0 (C_q), 128.4 (CH), 127.9 (CH), 127.7 (CH), 127.0 (CH), 123.8 (CH), 123.8 (CH), 118.2 (C_q), 118.2 (CH), 111.5 (CH), 98.9 (CH), 80.6 (C_q), 70.1 (CH_2), 50.0 (CH_2), 40.3 (CH), 37.5 (CH_2), 31.4 (C_q), 26.5 (CH_3), 21.3 (CH_3), 19.8 (CH), 18.9 (CH_2), 13.9 (C_q). **IR** (KBr): $\tilde{\nu}$ = 3384, 2924, 2853, 2363, 1609, 1573, 1286, 1260, 1232 cm^{-1}. **m.p.** (°C) = 128-130. **HRMS** (ESI): Calcd. for $C_{36}H_{31}N_4O_6$ [M+H]$^+$ 615.2244, found 615.2216.

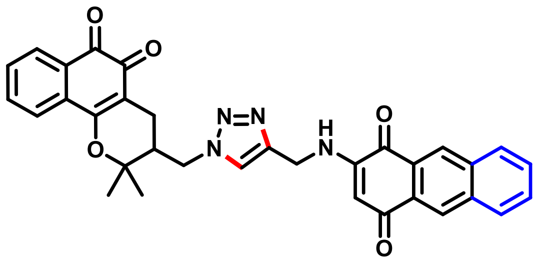

3-((4-(((1,4-dioxo-1,4-dihydroanthracen-2-yl)amino)methyl)-1*H*-1,2,3-triazol-1-yl)methyl)-2,2-dimethyl-3,4-dihydro-2*H*-benzo[h]chromene-5,6-dione (21e): The general procedure for the triazole synthesis was followed by using *N*-(1,4-dioxo-1,4-dihydronaphthalen-2-yl)acetamide (**10**) (43 mg, 0.2 mmol) and 2-(prop-2-yn-1-ylamino)anthracene-1,4-dione (**18e**) (58 mg, 0.22 mmol) as starting material. Purification by column chromatography on silica gel (*n*-hexane/EtOAc 4:1) yielded **21e** (66 mg, 59%)

as an orange solid. **^{1}H NMR** (400 MHz, DMSO-d_6) δ = 8.65 (s, 1H), 8.49 (s, 1H), 8.24 (d, *J* = 7.7 Mz, 1H), 8.19-8.18 (m, 2H), 8.06 (t, *J* = 6.3 Hz, 1H), 7.90 (d, *J* = 7.5 Hz, 1H), 7.76-7.68 (m, 4H), 7.62-7.58 (m, 1H), 5.88 (s, 1H), 4.64 (dd, *J* = 13.6, 4.4 Hz, 1H), 4.54 (d, *J* = 6.0 Hz, 2H), 4.30 (dd, *J* = 13.7, 10.2 Hz, 1H), 3.50 (s, 1H), 2.19 (dd, *J* = 17.5, 5.3 Hz, 1H), 2.06 (dd, *J* = 17.5, 9.3 Hz, 1H), 1.51 (s, 3H), 1.36 (s, 3H). **^{13}C NMR** (100 MHz, DMSO-d_6) δ = 181.6 (C_q), 181.4 (C_q), 179.6 (C_q), 178.0 (C_q), 160.5 (C_q), 149.6 (C_q), 143.8 (C_q), 135.4 (C_q), 135.4 (CH), 134.0 (C_q), 132.1 (C_q), 131.4 (CH), 130.6 (CH), 130.4 (C_q), 130.2 (CH), 130.2 (CH), 129.8 (C_q), 129.3 (CH), 128.9 (CH), 128.3 (CH), 127.9 (C_q), 127.1 (CH), 124.3 (CH), 124.2 (CH), 112.1 (C_q), 103.1 (CH), 80.8 (C_q), 70.2 (CH_2), 50.4 (CH_2), 40.7 (CH), 37.9 (CH_2), 26.8 (CH_3), 21.8 (CH_3) cm^{-1}. **IR** (KBr): $\tilde{\nu}$ = 3375, 2361, 1611, 1574, 1458, 1391, 1311, 1130 cm^{-1}. **m.p.** (°C) = 227-231 (decomposition). **HRMS** (ESI): Calcd. for $C_{33}H_{27}N_4O_5$ [M+H]$^+$ 559.1981, found 559.1956.

X-Ray Analysis

Single crystals of $C_{13}H_9NO_3$ (**18b**) were recrystallised from a mixture of acetonitrile and petroleum ether by solvent vapor diffusion. A suitable crystal was selected and mounted on a D8 Venture MoCu Dual Source diffractometer. The crystal was kept at 100.0 K during data collection.

Table S1. Crystal Data and structure refinement for **18b**.

**CCDC number**	2226471
**Identification code**	**18b**
**Empirical formula**	$C_{13}H_9NO_3$
**Formula weight**	227.22
**Temperature/K**	100.0
**Crystal system**	triclinic
**Space group**	P-1
**a/Å**	4.8309(2)
**b/Å**	10.8363(4)
**c/Å**	11.0191(4)
**α/°**	64.841(4)
**β/°**	89.551(3)
**γ/°**	79.853(3)
**Volume/Å^3**	512.49(4)
**Z**	2
**ρ_{calc}g/cm^3**	1.472
**μ/mm^{-1}**	0.882
**F(000)**	236
**Crystal size/mm^3**	0.244 × 0.094 × 0.030
**Radiation**	CuKα (λ = 1.54184)
**2Θ range for data collection/°**	4.440 to 79.220
**Index ranges**	$-6 \le h \le 6, -12 \le k \le 13, -13 \le l \le 14$
**Reflections collected**	11103
**Independent reflections**	1938 [R_{int} = 0.0395]
**Refined parameters**	155
**Goodness-of-fit on F^2**	1.062
**Final R indexes [I>=2σ (I)]**	R_1 = 0.0362
**Final R indexes [all data]**	wR_2 = 0.1007
**Largest diff. peak/hole / e Å^{-3}**	0.238/-0.321

Single crystals of $C_{14}H_{11}NO_3$ (**18c**) were recrystallised from a mixture of acetonitrile and petroleum ether by solvent vapor diffusion. A suitable crystal was selected and mounted on a D8 Venture MoCu Dual Source diffractometer. The crystal was kept at 100.0 K during data collection.

Table S2. Crystal Data and structure refinement for **18c**.

**CCDC number**	2226469
**Identification code**	**18c**
**Empirical formula**	$C_{14}H_{11}NO_3$
**Formula weight**	241.24
**Temperature/K**	100.0
**Crystal system**	monoclinic
**Space group**	$P2_1/n$
**a/Å**	7.9179(3)
**b/Å**	17.8842(4)
**c/Å**	8.2537(3)
**α/°**	90
**β/°**	106.660(4)
**γ/°**	90
**Volume/Å^3**	1119.71(7)
**Z**	4
**ρ_{calc}g/cm^3**	1.431
**μ/mm^{-1}**	0.840
**F(000)**	504
**Crystal size/mm^3**	0.150 × 0.092 × 0.021
**Radiation**	CuKα (λ = 1.54184)
**2Θ range for data collection/°**	4.941 to 77.268
**Index ranges**	$-9 \le h \le 9$, $-19 \le k \le 21$, $-10 \le l \le 9$
**Reflections collected**	10281
**Independent reflections**	2106 [R_{int} = 0.1117]
**Refined parameters**	164
**Goodness-of-fit on F^2**	1.101
**Final R indexes [I>=2σ (I)]**	R_1 = 0.0552
**Final R indexes [all data]**	wR_2 = 0.1509
**Largest diff. peak/hole / e Å^{-3}**	0.344/-0.366

Single crystals of $C_{20}H_{15}NO_3$ (**18d**) were recrystallised from a mixture of acetonitrile and petroleum ether by solvent vapor diffusion. A suitable crystal was selected and mounted on a D8 Venture MoCu Dual Source diffractometer. The crystal was kept at 100.0 K during data collection.

Table S3. Crystal Data and structure refinement for **18d**.

**CCDC number**	2226470
**Identification code**	**18d**
**Empirical formula**	$C_{20}H_{15}NO_3$
**Formula weight**	317.33
**Temperature/K**	273(2)
**Crystal system**	monoclinic
**Space group**	$P2_1/c$
**a/Å**	10.25400(10)
**b/Å**	7.84020(10)
**c/Å**	19.6671(2)
**α/°**	90
**β/°**	104.1050(10)
**γ/°**	90
**Volume/Å³**	1533.44(3)
**Z**	4
ρ_{calc}**g/cm³**	1.375
**μ/mm⁻¹**	0.755
**F(000)**	664
**Crystal size/mm³**	0.217 × 0.109 × 0.042
**Radiation**	CuKα (λ = 1.54184)
**2Θ range for data collection/°**	4.446 to 76.134
**Index ranges**	-12 ≤ h ≤ 12, -9 ≤ k ≤ 9, -18 ≤ l ≤ 24
**Reflections collected**	27872
**Independent reflections**	3141 [R_{int} = 0.0259]
**Refined parameters**	217
**Goodness-of-fit on F²**	1.059
**Final R indexes [I>=2σ (I)]**	R_1 = 0.0354
**Final R indexes [all data]**	wR_2 = 0.0908
**Largest diff. peak/hole / e Å⁻³**	0.209/-0.232

Antitumor Assays

The *in vitro* cytotoxicity activity of compounds was evaluated by the colorimetric MTT (3-(4,5-dimethylthiazol-2-yl)-2,5-diphenyl tetrazolium bromide)) assay [56] using tumor cell lines: HCT-116 (colon carcinoma), PC3 (prostate), SNB-19 (glioblastoma), K-562 (myelogenous leukaemia), HL-60 (human promyelocytic leukaemia), B16 (murine melanoma), A549 (human lung carcinoma), KG1 (human acute myeloid leukaemia) and RAJI (human Burkitt's lymphoma), which were provided by the National Cancer Institute (Bethesda, MD, USA). The L929 cell line (mouse fibroblast L cells NCTC clone 929) employed in this study as a control cell line was obtained from the American Type Culture

Collection (Manassas, VA, USA). Cell lines (Table 01) were maintained in flasks containing RPMI 1640 or DMEM medium supplemented with 10% bovive fetal sérum, 100 U/mL penicillin, and 100 μg/mL streptomycin at 37 °C and 5% CO_2 atmosphere. Compounds tested were dissolved in DMSO. Doxorubicin served as positive control. Cell treatments were performed with three replicates and cells were mycoplasma-free. After 72 h incubation, 100 μL of MTT solution (0.5 mg/mL) was added to each well, and cells were incubated for 3 h. The supernatant was removed and 100 μL of DMSO was added and the absorbance at 595 nm was measured using Victor Nivo Multimode plate reader (PerkinElmer, Waltham, MA, USA). The absorbances obtained were used to calculate the IC_{50} values by nonlinear regression employing appropriate statistical software [56].

Table S4. Cell lines, disease, medium and cell seeding concentration used in MTT assay.

Cell Line	Cancer type	Medium	Concentration
**HCT-116**	Human colon carcinoma	RPMI 1640 + 10% Bovine fetal serum + 100 U/mL penicillin + 100 μg/mL streptomycin	7 x 10^4
**PC3**	Human prostatic carcinoma	RPMI 1640 + 10% Bovine fetal serum + 100 U/mL penicillin + 100 μg/mL streptomycin	10 x 10^4
**SNB-19**	Human glioblastoma	RPMI 1640 + 10% Bovine fetal serum + 100 U/mL penicillin + 100 μg/mL streptomycin	10 x 10^4
**K-562**	Human myelogenous leukemia	RPMI 1640 + 10% Bovine fetal serum + 100 U/mL penicillin + 100 μg/mL streptomycin	30 x 10^4
**HL-60**	Human promyelocytic leukemia	RPMI 1640 + 10% Bovine fetal serum + 100 U/mL penicillin + 100 μg/mL streptomycin	30 x 10^4
**B16**	Murine melanoma	DMEM + 10% Bovine fetal serum + 100 U/mL penicillin + 100 μg/mL streptomycin	3 x 10^4
**A549**	Human lung adenocarcinoma	RPMI 1640 + 10% Bovine fetal serum + 100 U/mL penicillin + 100 μg/mL streptomycin	7,5 x 10^4
**KG1**	Human acute myelogenous leukemia	IMDM + 20% Bovine fetal serum + 100 U/mL penicillin + 100 μg/mL streptomycin	30 x 10^4
**RAJI**	Human Burkitt's lymphoma	RPMI 1640 + 10% Bovine fetal serum + 0.45% glucose + 100 U/mL penicillin + 100 μg/mL streptomycin	30 x 10^4
**L929**	Non-tumoral mouse fibroblast	DMEM + 10% Bovine fetal serum + 100 U/mL penicillin + 100 μg/mL streptomycin	7 x 10^4

Figure S1. Cytotoxic effects of compounds on tumor cell lines for 72 h. L929 was used as non-tumoral control. The continuous line represents the fitted dose-response curve.

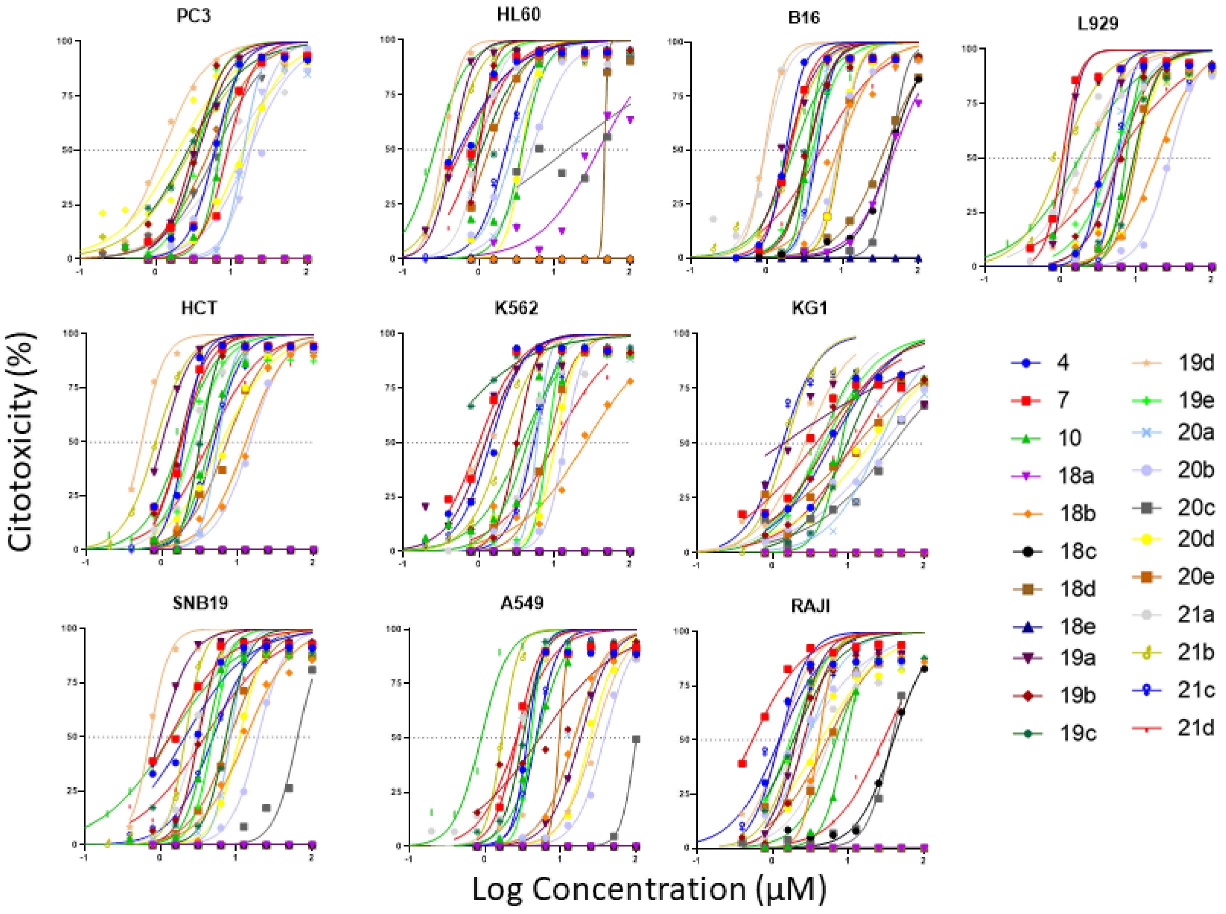

References

32. Lima, D.B.J.; Almeida, R.G.; Jardim, G.A.M.; Barbosa, B.P.A.; Santos, A.C.C.; Valença, W.O.; Scheide, M.R.; Gatto, C.C.; de Carvalho, G.G.C.; Costa, P.M.S.; Pessoa, C.; Pereira, C.L.M.; Jacob, C.; Braga, A.L.; da Silva Júnior, E.N. It takes two to tango: synthesis of cytotoxic quinones containing two redox active centers with potential antitumor activity. *RSC Med. Chem.* **2021**, *12*, 1709-1721.
36. Jardim, G.A.M.; Oliveira, W.X.C.; Freitas, R.P.; Menna-Barreto, R.F.S.; Silva, T.L.; Goulart, M.O.F.; da Silva Júnior, E.N. Direct sequential C–H iodination/organoyl-thiolation for the benzenoid A-ring modification of quinonoid deactivated systems: a new protocol for potent trypanocidal quinones. *Org. Biomol. Chem.* **2018**, *16*, 1686-1691.
43. da Silva Júnior, E.N.; Menna-Barreto, R.F.S.; Pinto, M.C.F.R.; Silva, R.S.F.; Teixeira, D.V.; de Souza, M.C.B.V.; de Simone, C.A.; de Castro, S.L.; Ferreira, V.F.; Pinto, A.V. Naphthoquinoidal [1,2,3]-triazole, a new structural moiety active against *Trypanosoma cruzi*. *Eur. J. Med. Chem.* **2008**, *43*, 1774-1780.
44. Jardim, G.A.M.; Cruz, E.H.G.; Valença, W.O.; Resende, J.M.; Rodrigues, B.L.; Ramos, D.F.; Oliveira, R.N.; Silva, P.E.A.; da Silva Júnior, E.N. On the search for potential antimycobacterial drugs: synthesis of naphthoquinoidal, phenazinic and 1,2,3-triazolic compounds and evaluation against *Mycobacterium tuberculosis*. *J. Braz. Chem. Soc.* **2015**, *26*, 1013-1027.
45. Mezeiova, E.; Janockova, J.; Andrys, R.; Soukup, O.; Kobrlova, T.; Muckova, L.; Pejchal, J.; Simunkova, M.; Handl, J.; Micankova, P.; Capek, J.; Rousar, T.; Hrabinova, M.; Nepovimova, E.; Marco-Contelles, J.L.; Valko, M.; Korabecny, J. 2-Propargylamino-naphthoquinone derivatives as multipotent agents for the treatment of Alzheimer's disease. *Eur. J. Med. Chem.* **2021**, *211*, 113112.
46. Jardim, G.A.M.; da Silva Júnior, E.N.; Bower, J.F. Overcoming naphthoquinone deactivation: rhodium-catalyzed C-5 selective C–H iodination as a gateway to functionalized derivatives. *Chem. Sci.* **2016**, *7*, 3780-3784.
47. Dolomanov, O.V.; Bourhis, L.J.; Gildea, R.J.; Howard, J.A.K.; Puschmann, H. OLEX2: a complete structure solution, refinement and analysis program. *J. Appl. Cryst.* **2009**, *42*, 339-341.
48. Sheldrick, G.M. SHELXT – Integrated space-group and crystal-structure determination. *Acta Cryst. Section A* **2015**, *71*, 3-8.
49. Sheldrick, G.M.A short history of SHELX. *Acta Cryst. Section A* **2008**, *64*, 112-122.
50. Kongkathip, N.; Kongkathip, B.; Siripong, P.; Sangma, C.; Luangkamin, S.; Niyomdecha, M.; Pattanapa, S.; Piyaviriyagul, S.; Kongsaeree, P. Potent antitumor activity of synthetic 1,2-naphthoquinones and 1,4-naphthoquinones. *Bioorg. Med. Chem.* **2003**, *11*, 3179-3191.
51. Silva, R.S.F.; Costa, E.M.; Trindade, U.L.T.; Teixeira, D.V.; Pinto, M.C.F.R.; Santos, G.L.; Malta, V.R.S.; de Simone, C.A.; Pinto, A.V.; de Castro, S.L. Synthesis of naphthofuranquinones with activity against *Trypanosoma cruzi*. *Eur. J. Med. Chem.* **2006**, *41*, 526-530.
52. Montenegro, R.C.; Araújo, A.J.; Molina, M.T.; Filho, J.D.B.M.; Rocha, D.D.; López-Montero, E.; Goulart, M.O.F.; Bento, E.S.; Alves, A.P.N.N.; Pessoa, C.; Moraes, M.O.; Costa-Lotufo, L.V. Cytotoxic activity of naphthoquinones with special emphasis on juglone and its 5-*O*-methyl derivative. *Chem.-Biol. Int.* **2010**, *184*, 439-448.
53. Shu, X.; Chen, C.-C.; Yu, T.; Yang, J.; Hu, X. Enantioselective total synthesis of (−)-Spiroxins A, C, and D. *Angew. Chem. Int. Ed.* **2021**, *60*, 18514-18518.
54. Li, D.; Shen, X. Iron-catalyzed regioselective alkylation of 1,4-quinones and coumarins with functionalized alkyl bromides. *Org Biomol. Chem.* **2020**, *18*, 750-754.
55. Nor, S.M.M.; Sukari, M.A.H.M.; Azziz, S.S.S.A.; Fah, W.C.; Alimon, H.; Juhan, S.F. Synthesis of new cytotoxic aminoanthraquinone derivatives *via* nucleophilic substitution reactions. *Molecules* **2013**, *18*, 8046-8062.
56. Mosmann, T. Rapid colorimetric assay for cellular growth and survival: application to proliferation and cytotoxicity assays. *J. Immunol. Methods* **1983**, *65*, 55-63.

^{1}H and ^{13}C NMR Spectra

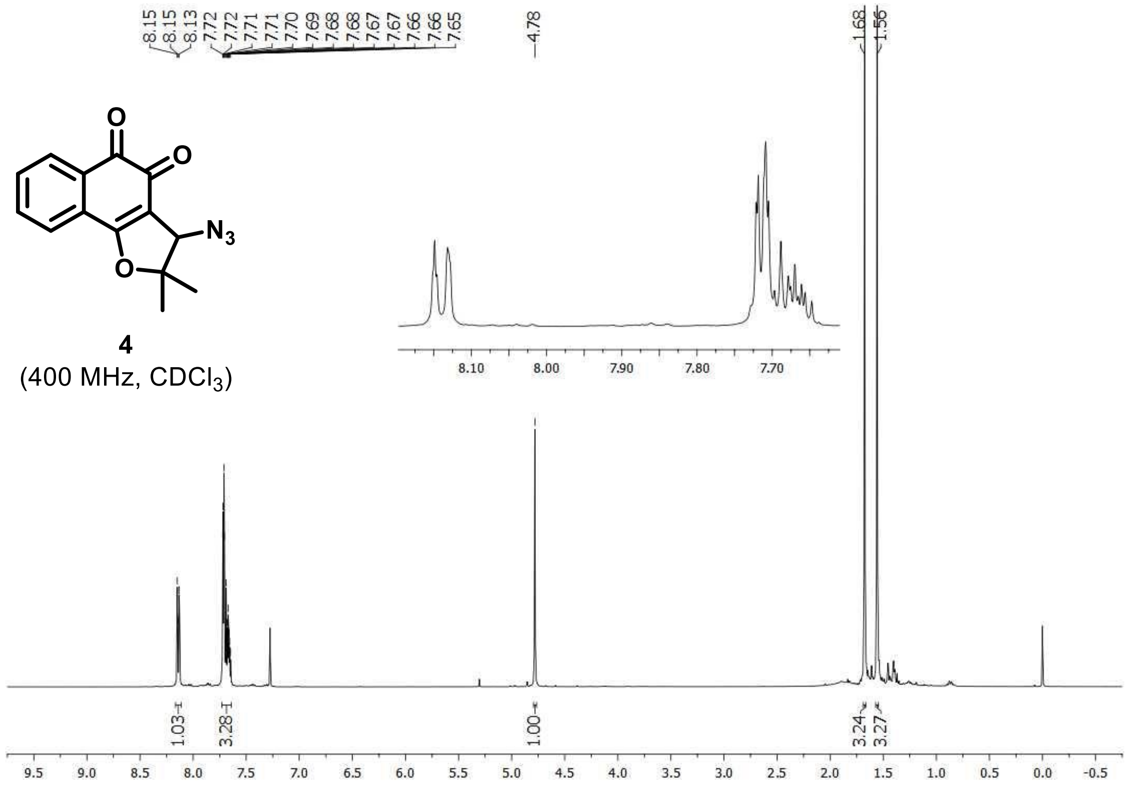

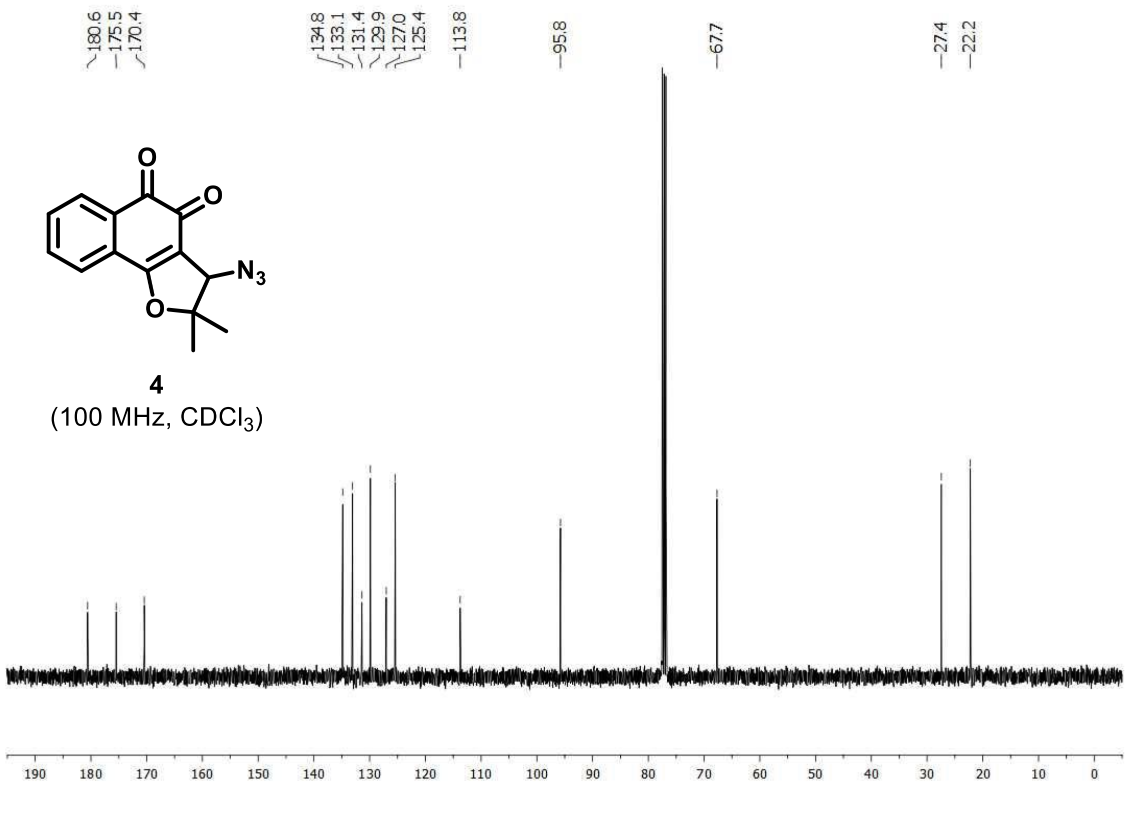

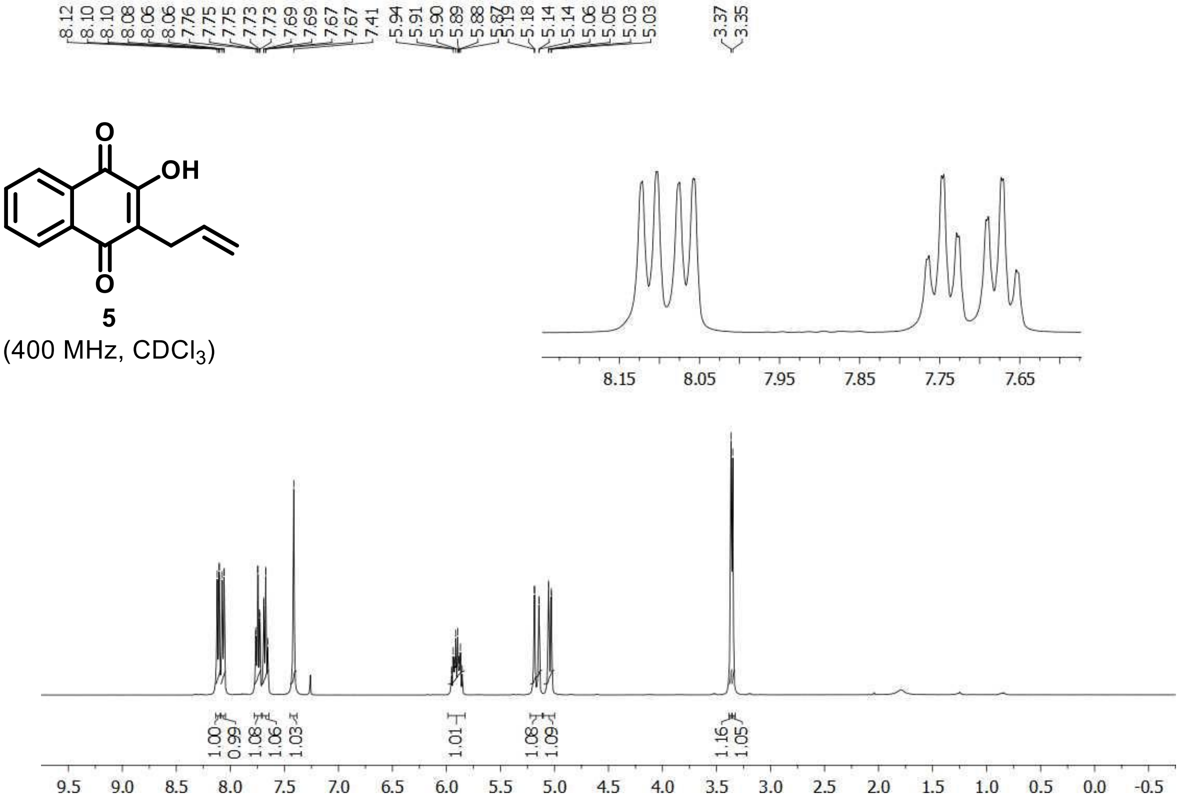
OH
O
O
5
(400 MHz, CDCl3)

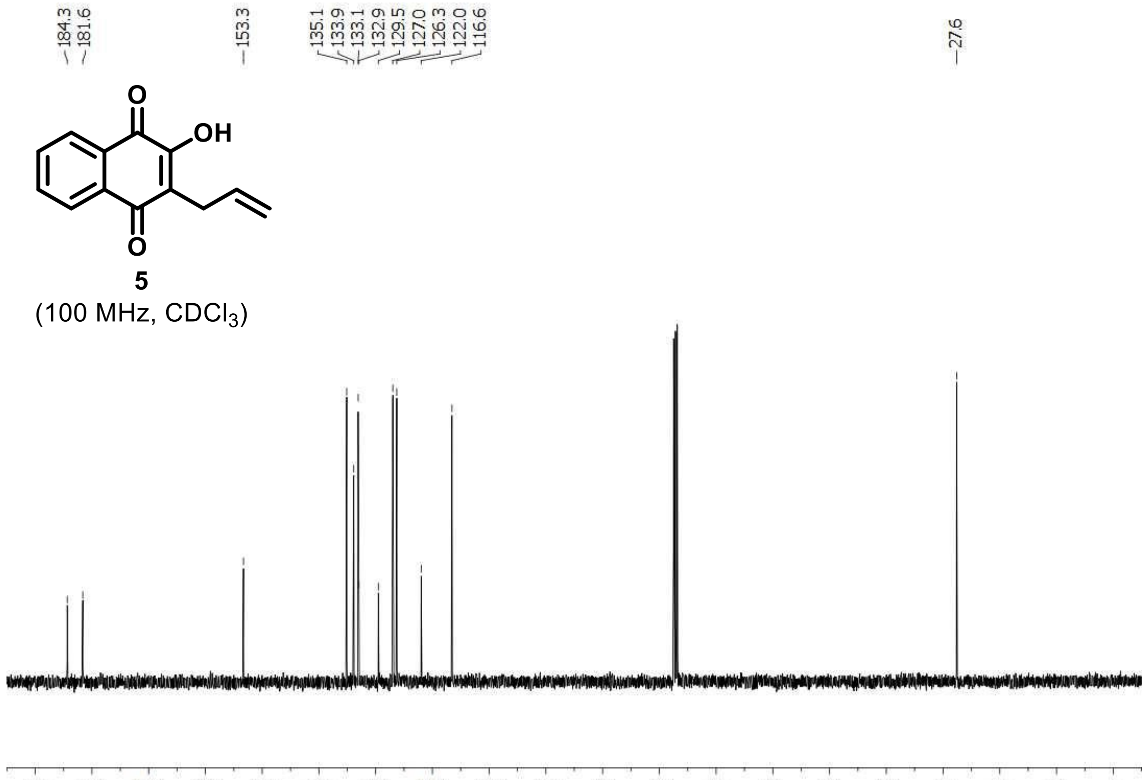
OH
O
O
5
(100 MHz, CDCl3)

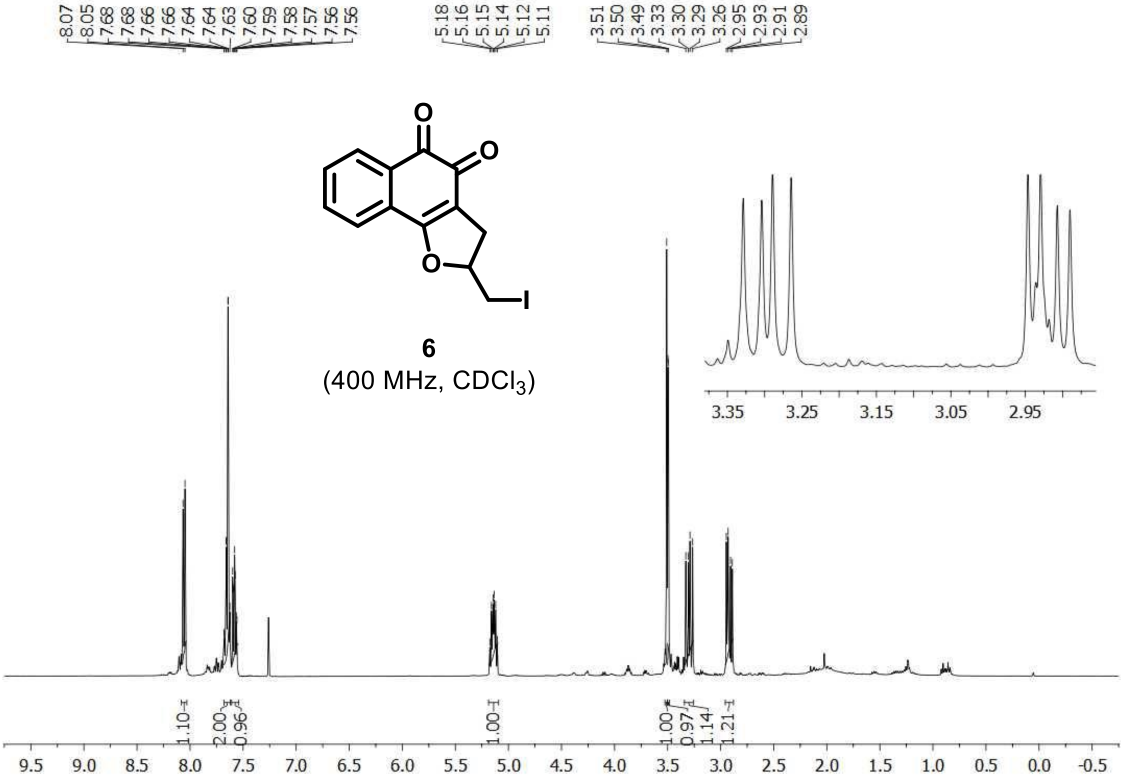
8.07
8.05
7.68
7.68
7.66
7.66
7.64
7.64
7.63
7.60
7.59
7.58
7.57
7.56
7.56
5.18
5.16
5.15
5.14
5.12
5.11
3.51
3.50
3.49
3.33
3.30
3.29
3.26
2.95
2.93
2.91
2.89
6
(400 MHz, CDCl3)
3.35
3.25
3.15
3.05
2.95
1.10
2.00
0.96
1.00
1.00
0.97
1.14
1.21
9.5
9.0
8.5
8.0
7.5
7.0
6.5
6.0
5.5
5.0
4.5
4.0
3.5
3.0
2.5
2.0
1.5
1.0
0.5
0.0
-0.5

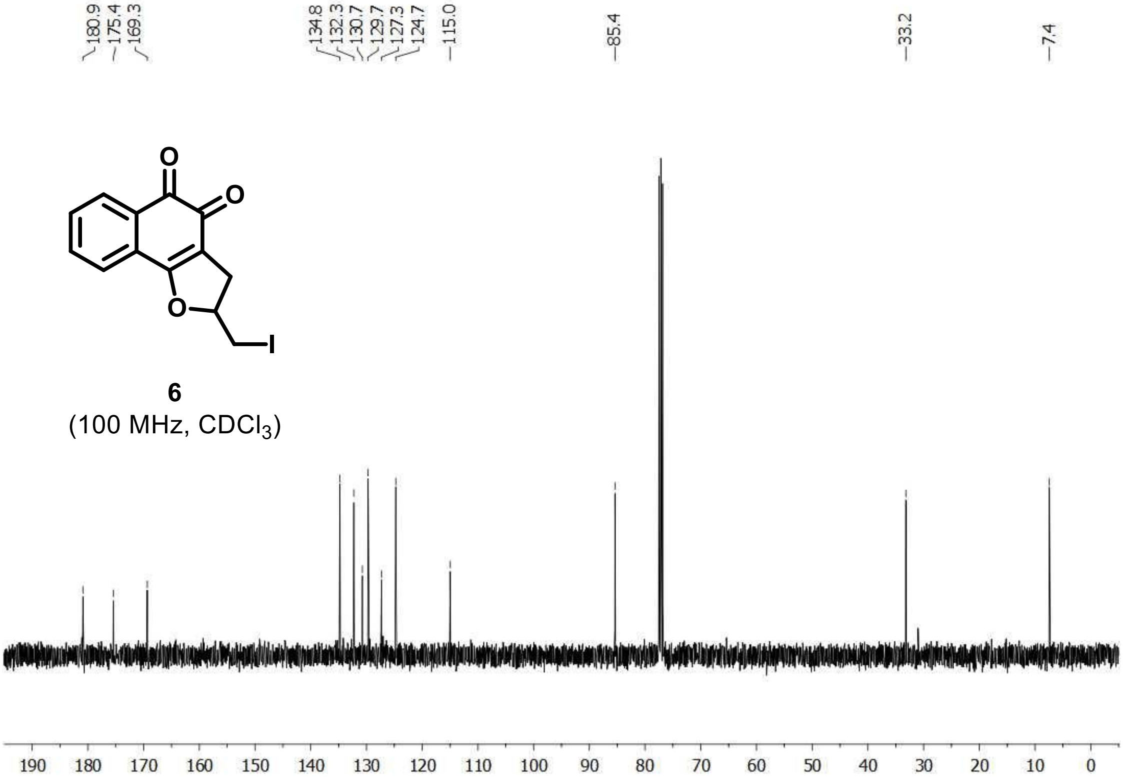
180.9
175.4
169.3
134.8
132.3
130.7
129.7
127.3
124.7
115.0
85.4
33.2
7.4
6
(100 MHz, CDCl3)
190
180
170
160
150
140
130
120
110
100
90
80
70
60
50
40
30
20
10
0

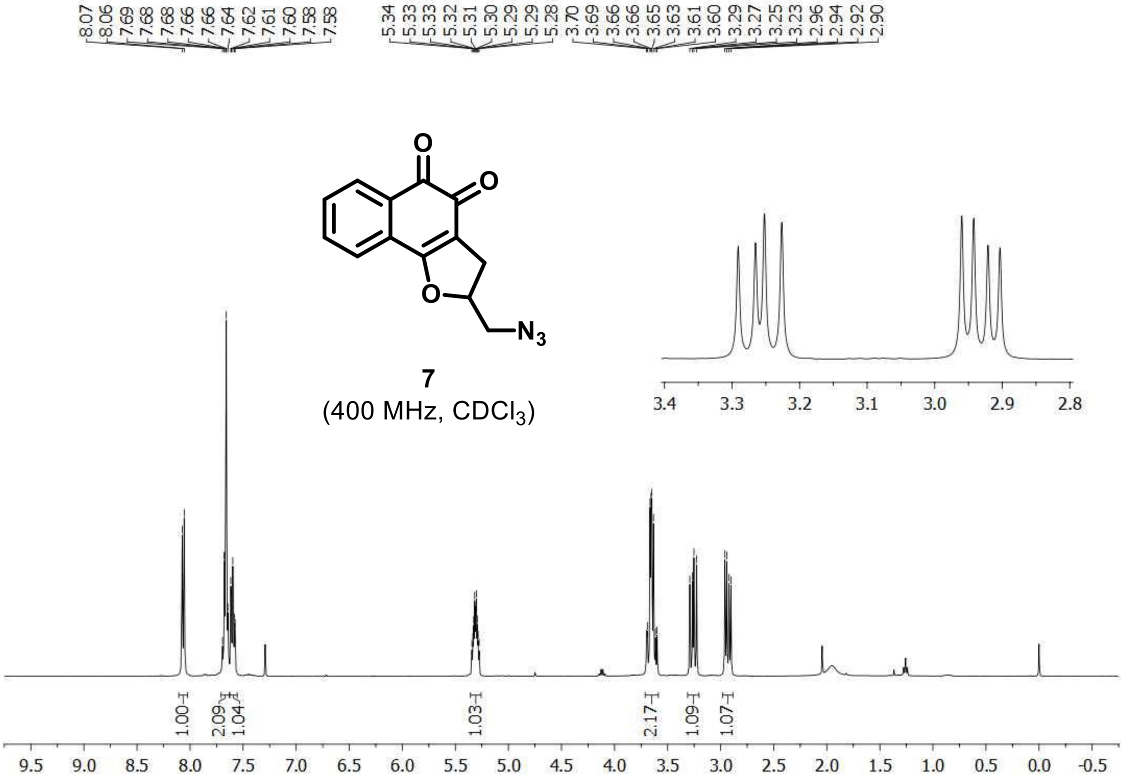
8.07
8.06
7.69
7.68
7.68
7.66
7.66
7.64
7.62
7.61
7.60
7.58
7.58
5.34
5.33
5.33
5.32
5.31
5.30
5.29
5.29
5.28
3.70
3.69
3.66
3.66
3.65
3.63
3.61
3.60
3.29
3.27
3.25
3.23
2.96
2.94
2.92
2.90
O
O
O
N3
7
(400 MHz, CDCl3)
1.00
2.09
1.04
1.03
2.17
1.09
1.07

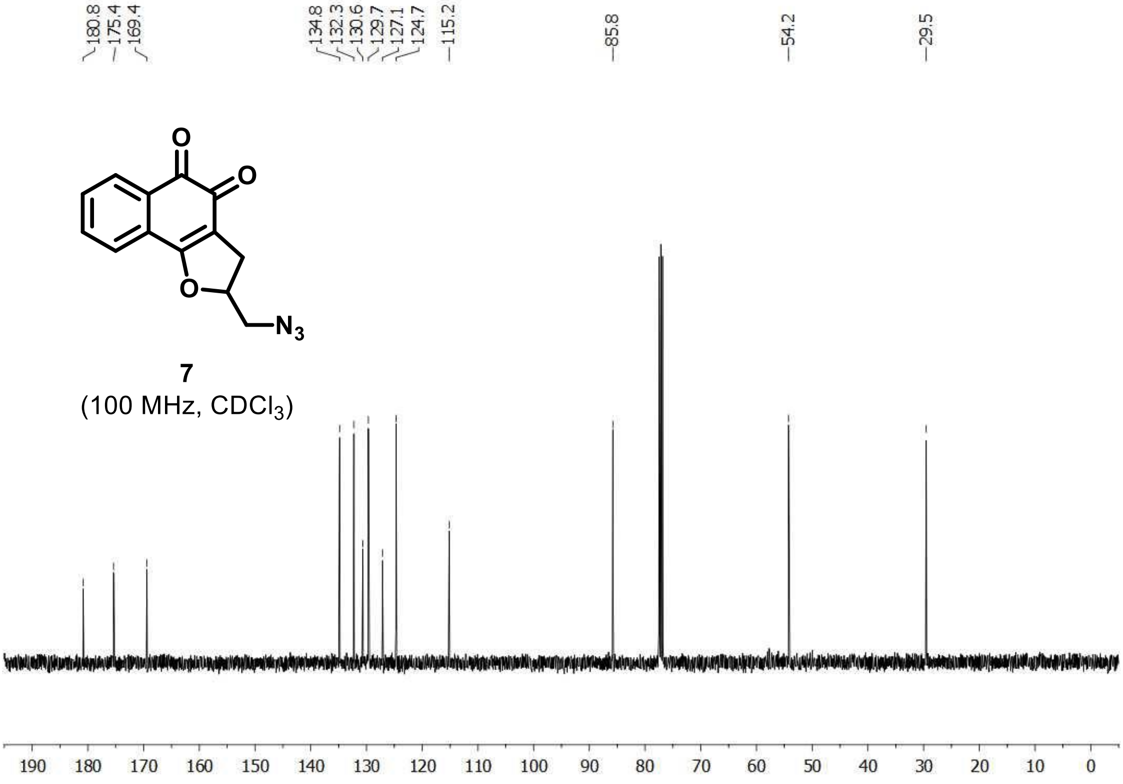
180.8
175.4
169.4
134.8
132.3
130.6
129.7
127.1
124.7
115.2
85.8
54.2
29.5
O
O
O
N3
7
(100 MHz, CDCl3)

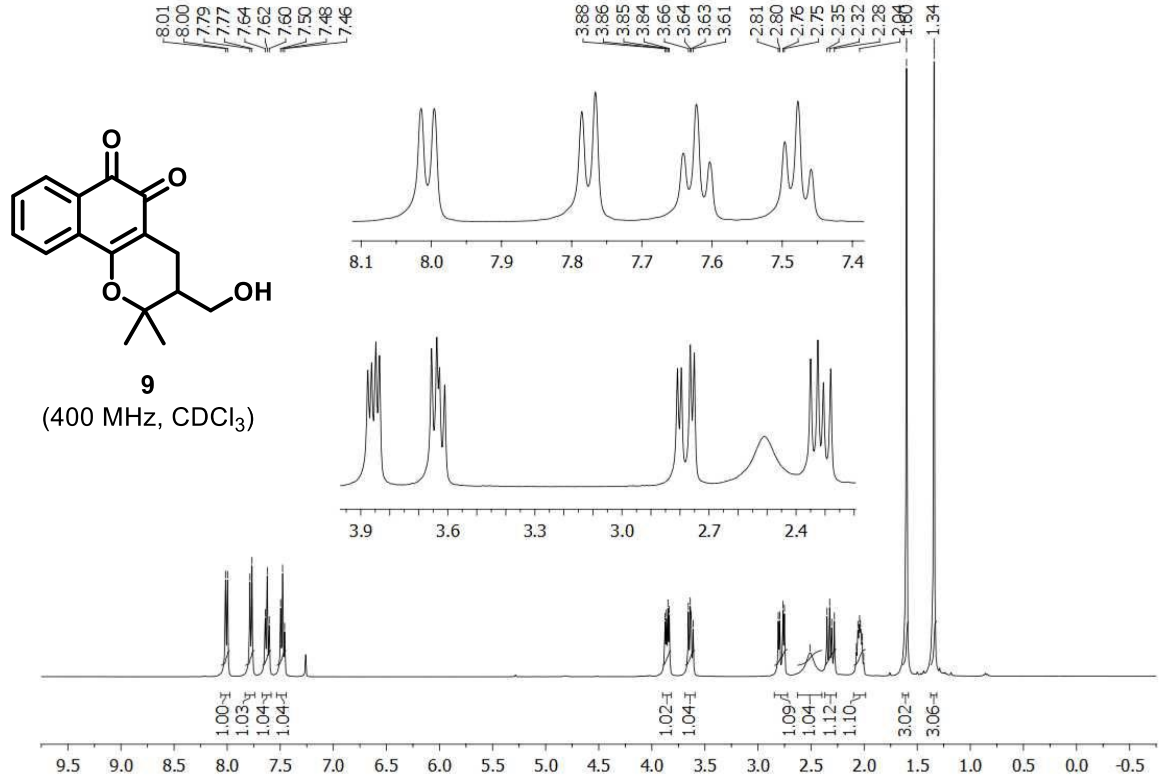
9
(400 MHz, CDCl3)
8.01 8.00 7.79 7.77 7.64 7.62 7.60 7.50 7.48 7.46
3.88 3.86 3.85 3.84 3.66 3.64 3.63 3.61
2.81 2.80 2.76 2.75 2.35 2.32 2.28 2.04 1.60 1.34
1.00 1.03 1.04 1.04 1.02 1.04 1.09 1.04 1.12 1.10 3.02 3.06
9.5 9.0 8.5 8.0 7.5 7.0 6.5 6.0 5.5 5.0 4.5 4.0 3.5 3.0 2.5 2.0 1.5 1.0 0.5 0.0 -0.5

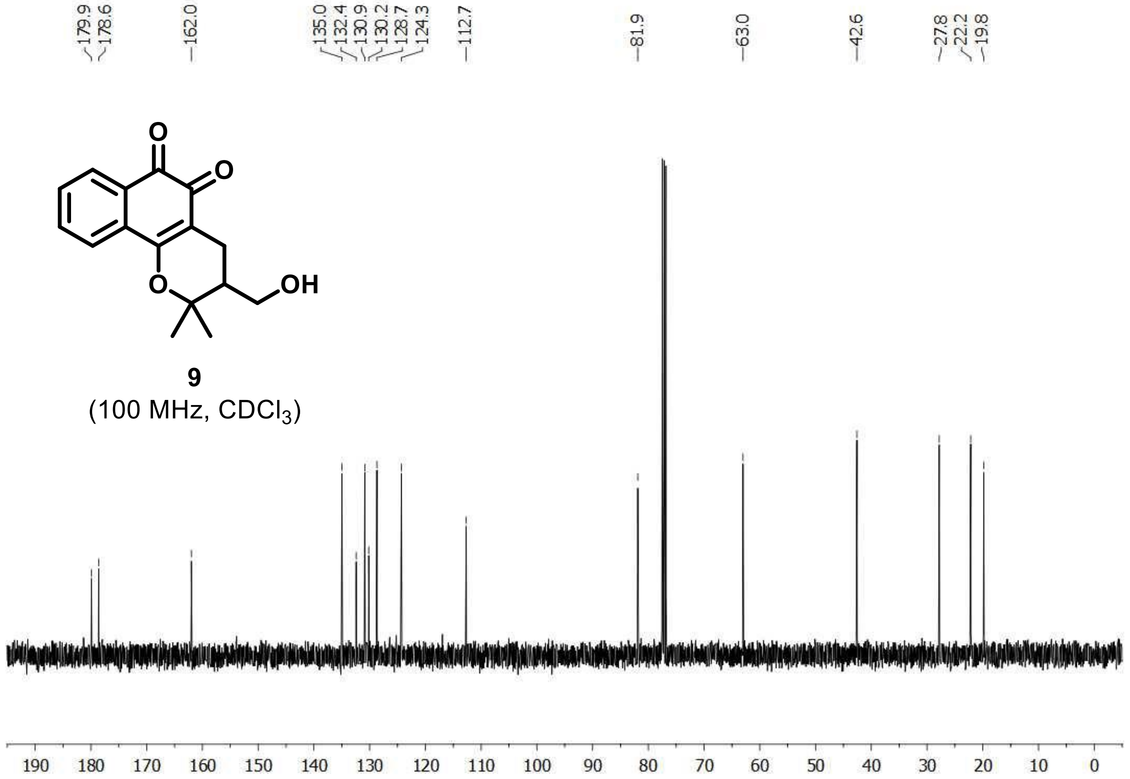
9
(100 MHz, CDCl3)
179.9 178.6 162.0 135.0 132.4 130.9 130.2 128.7 124.3 112.7 81.9 63.0 42.6 27.8 22.2 19.8
190 180 170 160 150 140 130 120 110 100 90 80 70 60 50 40 30 20 10 0

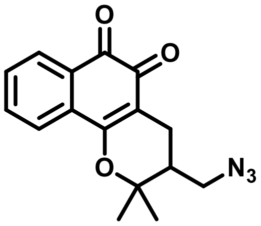

10
(400 MHz, $CDCl_3$)

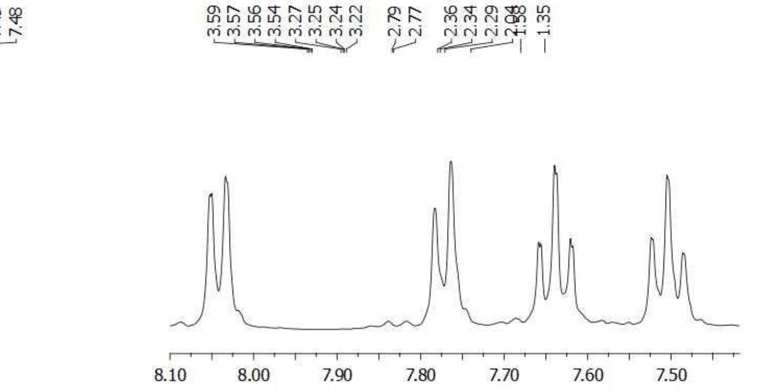

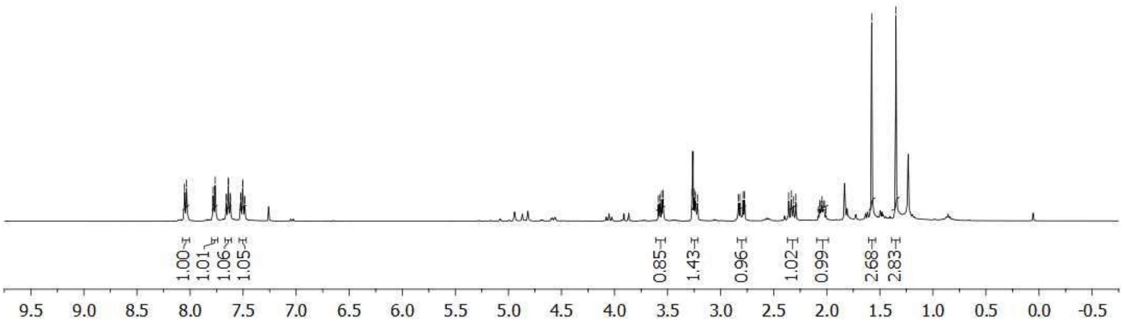

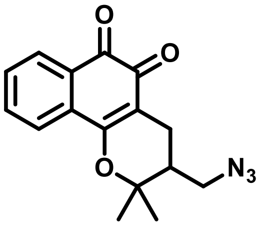

10
(100 MHz, $CDCl_3$)

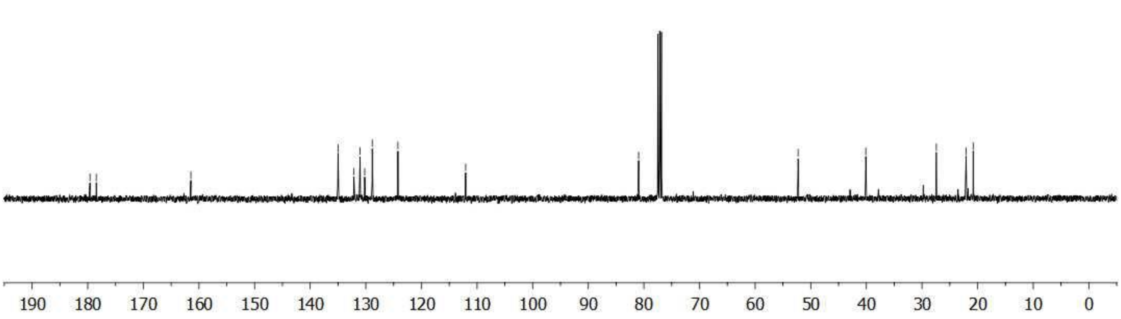

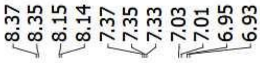

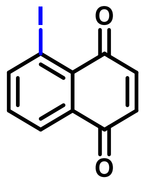

12
($CDCl_3$, 400 MHz)

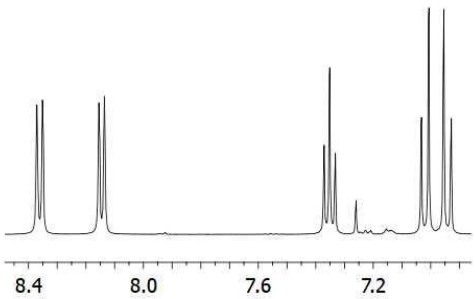

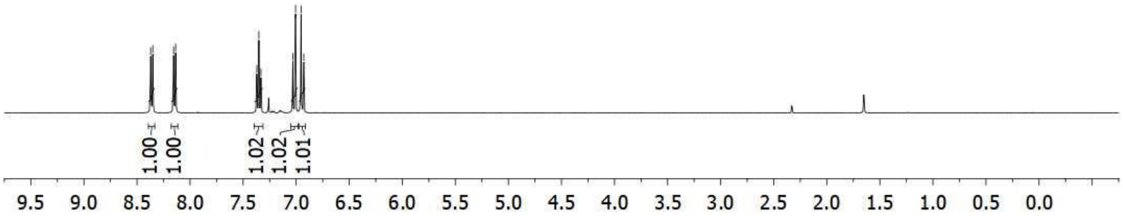

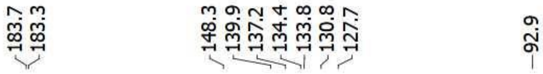

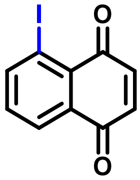

12
($CDCl_3$, 100 MHz)

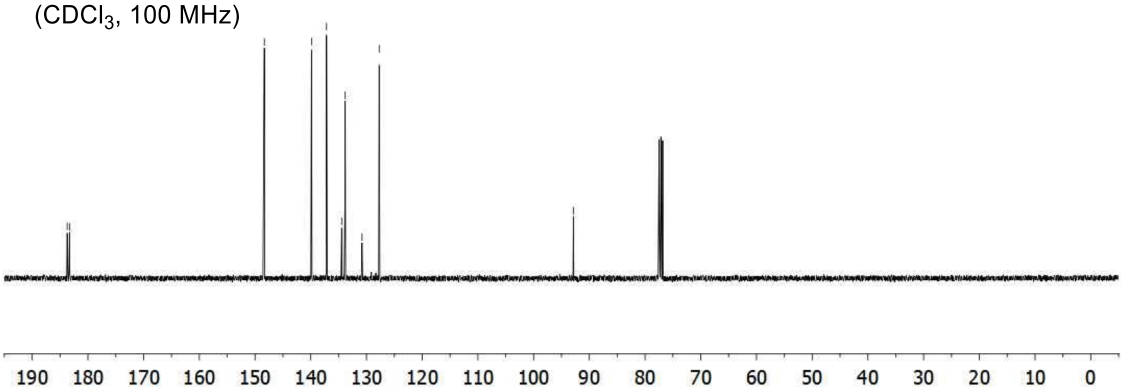

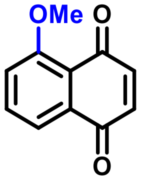

14
($CDCl_3$, 400 MHz)

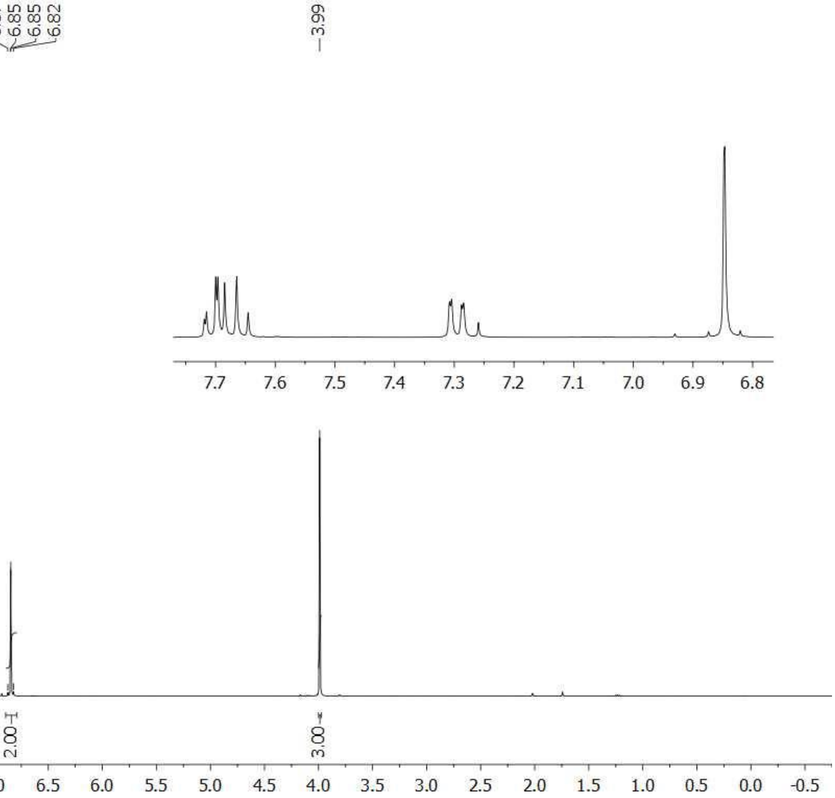

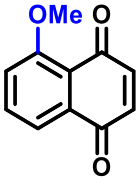

14
($CDCl_3$, 100 MHz)

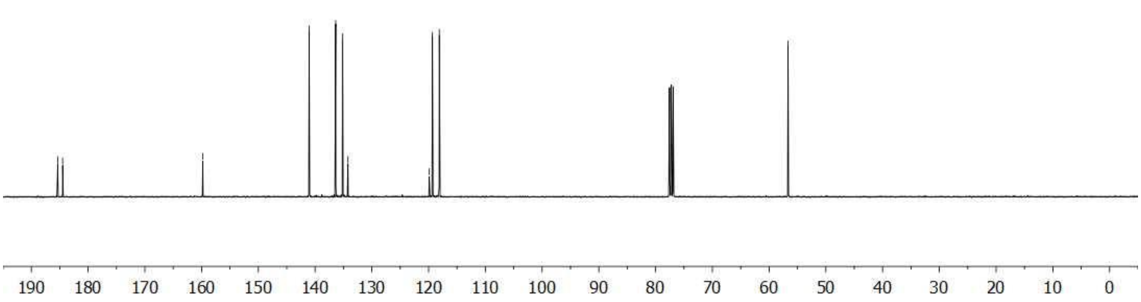

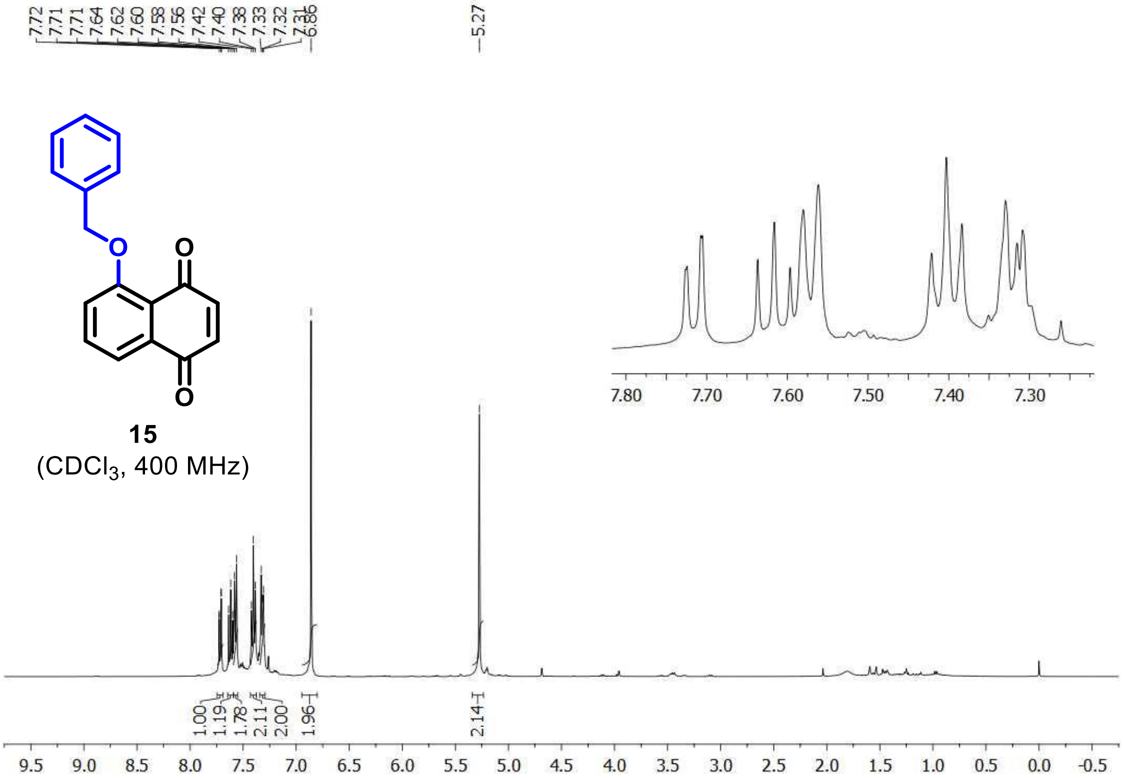
7.72
7.71
7.71
7.64
7.62
7.60
7.58
7.56
7.42
7.40
7.38
7.33
7.32
7.31
6.86
5.27
15
(CDCl$_3$, 400 MHz)
7.80 7.70 7.60 7.50 7.40 7.30
1.00
1.19
1.78
2.11
2.00
1.96
2.14
9.5 9.0 8.5 8.0 7.5 7.0 6.5 6.0 5.5 5.0 4.5 4.0 3.5 3.0 2.5 2.0 1.5 1.0 0.5 0.0 -0.5

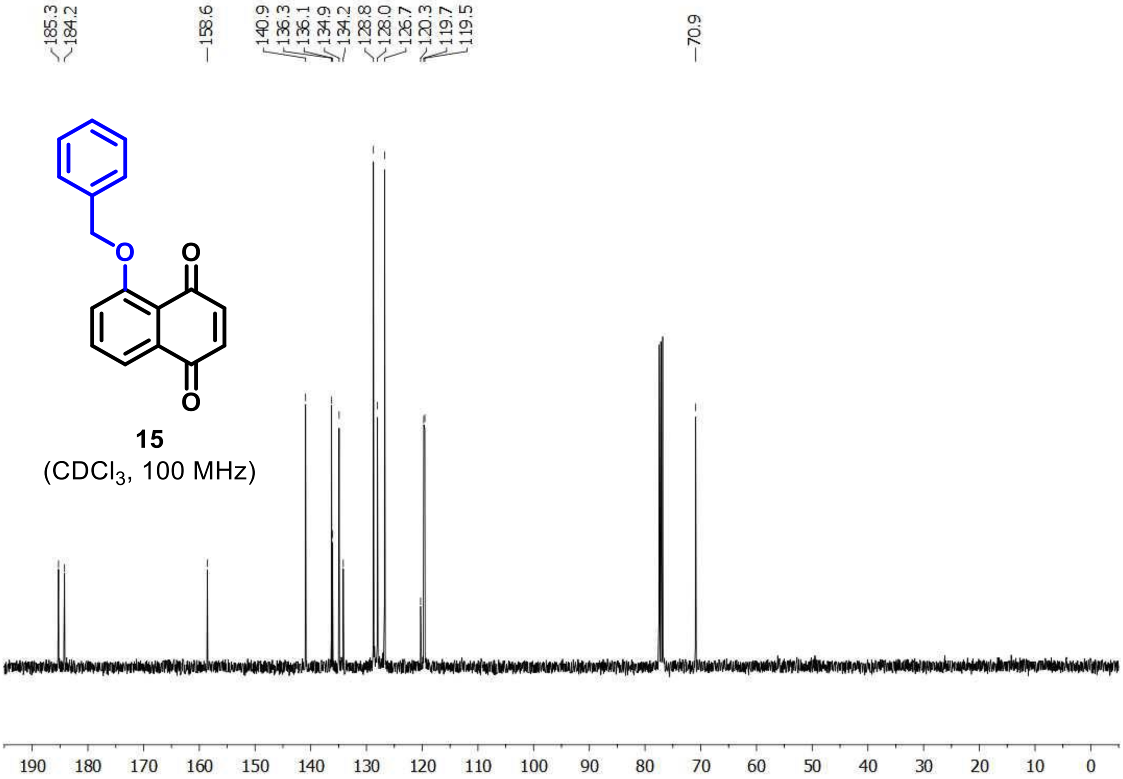
185.3
184.2
158.6
140.9
136.3
136.1
134.9
134.2
128.8
128.0
126.7
120.3
119.7
119.5
70.9
15
(CDCl$_3$, 100 MHz)
190 180 170 160 150 140 130 120 110 100 90 80 70 60 50 40 30 20 10 0

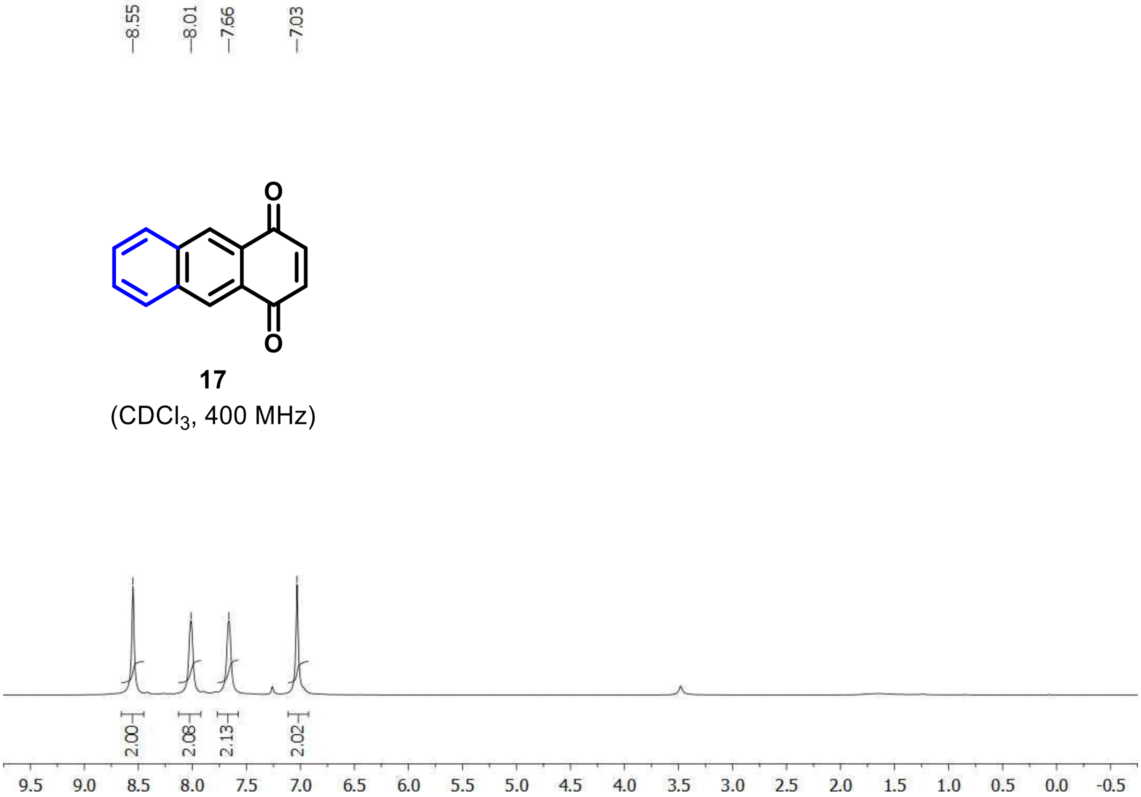
8.55
8.01
7.66
7.03
17
(CDCl3, 400 MHz)
2.00
2.08
2.13
2.02
9.5 9.0 8.5 8.0 7.5 7.0 6.5 6.0 5.5 5.0 4.5 4.0 3.5 3.0 2.5 2.0 1.5 1.0 0.5 0.0 -0.5

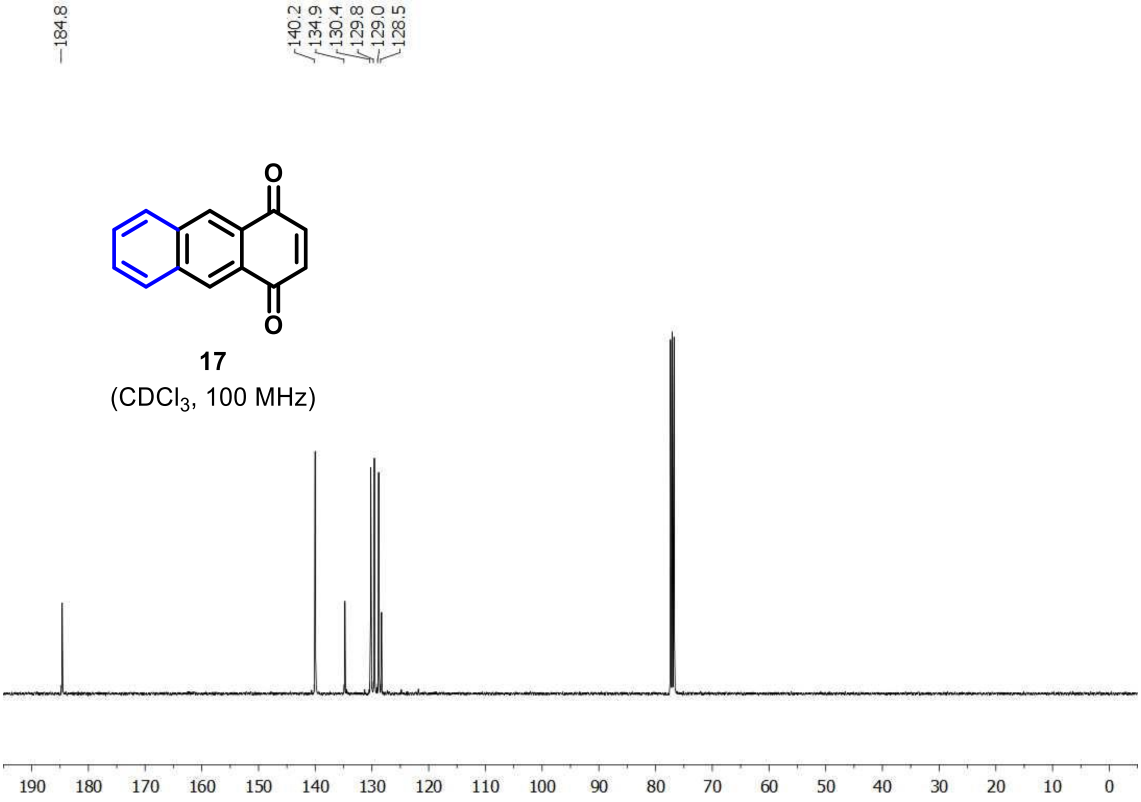
184.8
140.2
134.9
130.4
129.8
129.0
128.5
17
(CDCl3, 100 MHz)
190 180 170 160 150 140 130 120 110 100 90 80 70 60 50 40 30 20 10 0

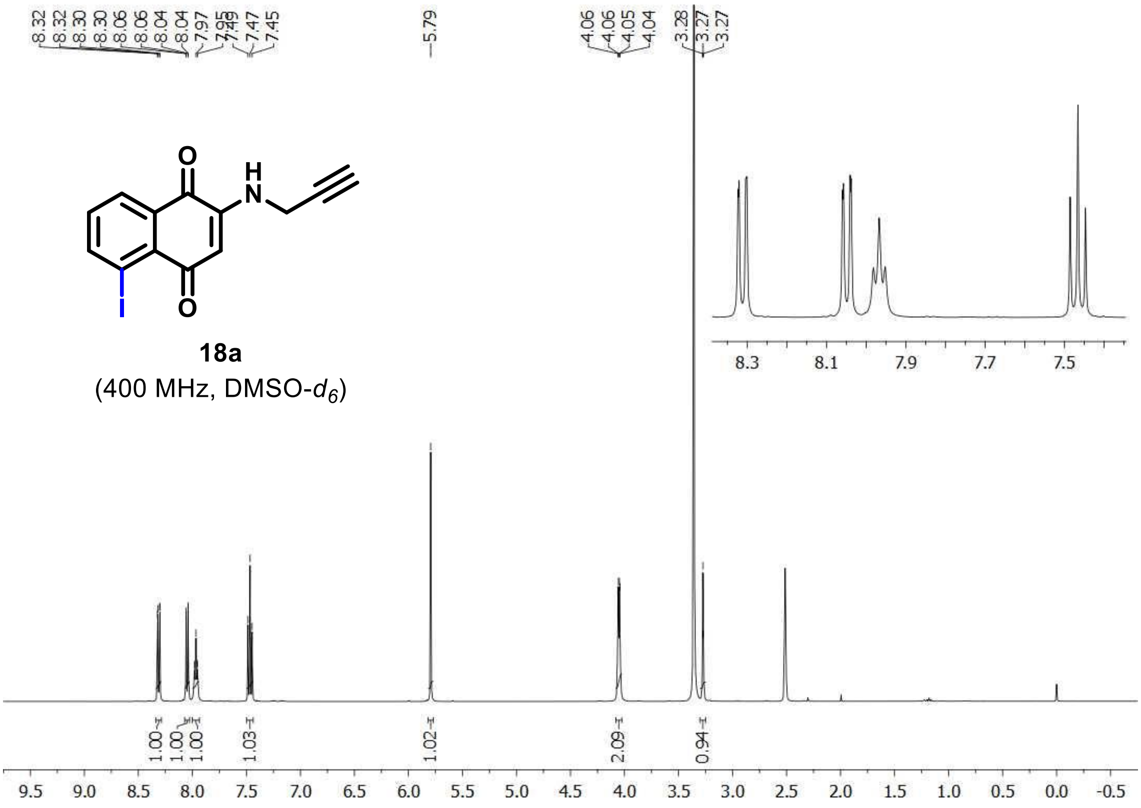
18a
(400 MHz, DMSO-d6)

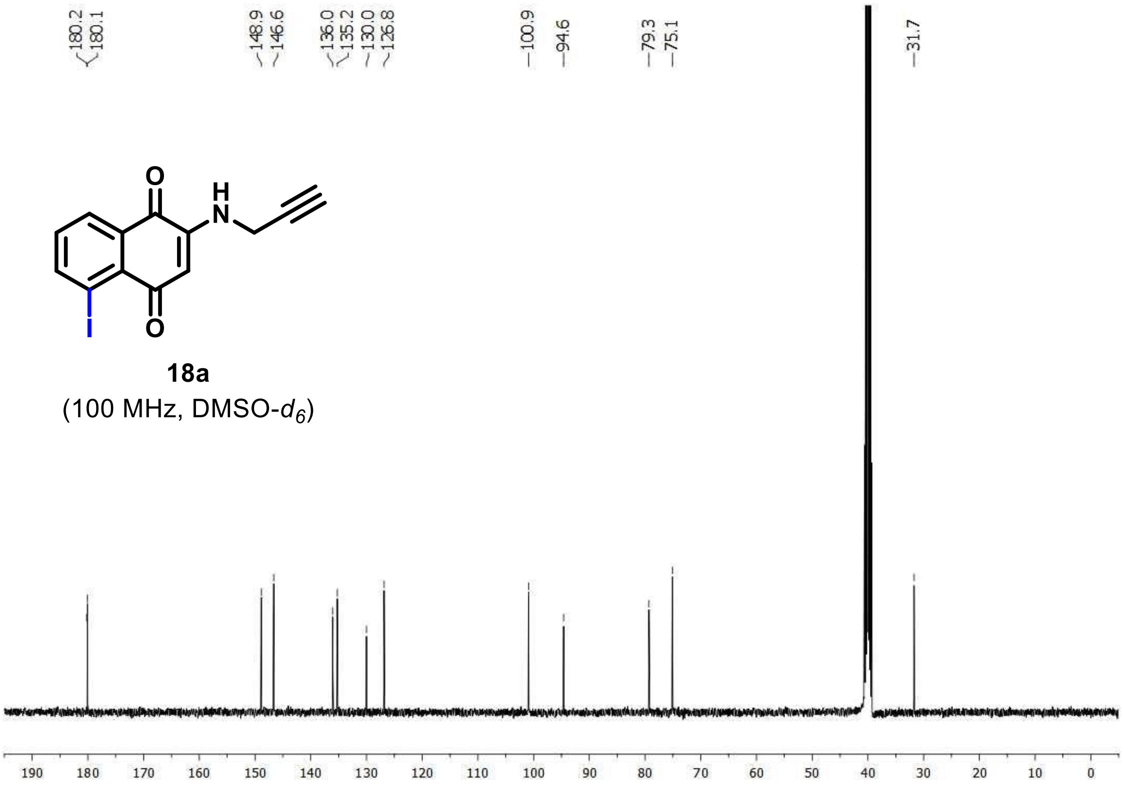
18a
(100 MHz, DMSO-d6)

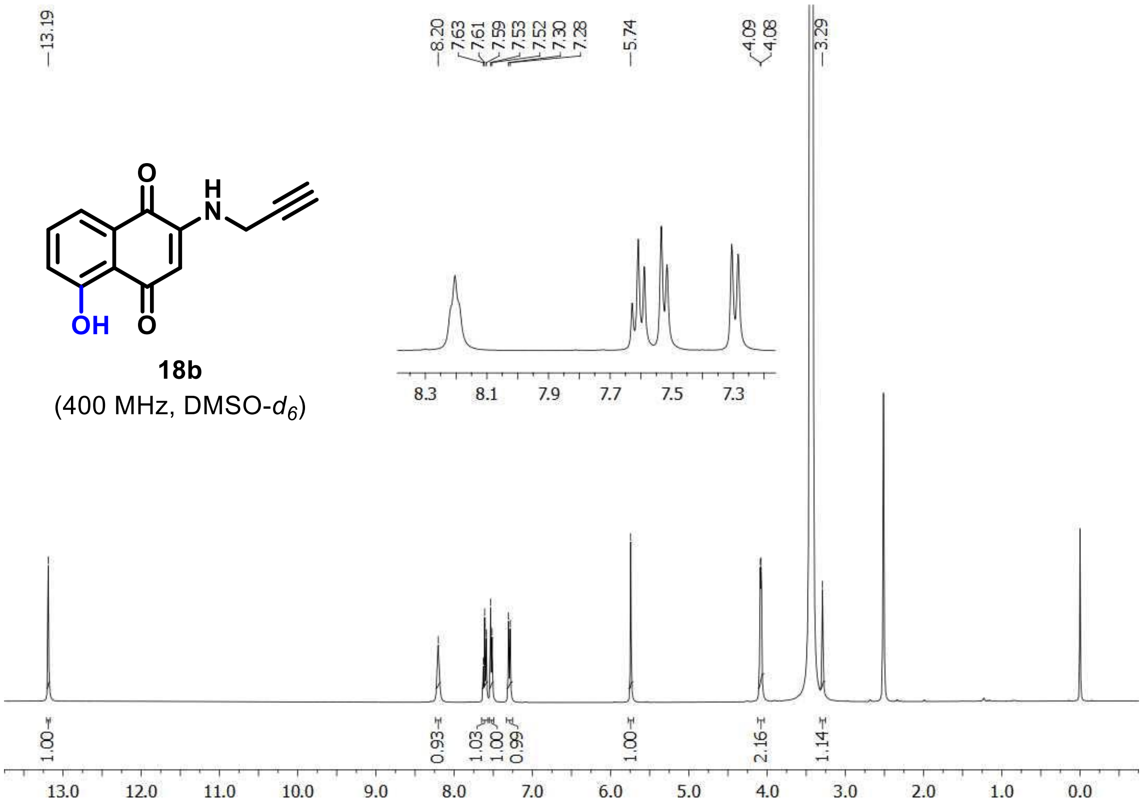
13.19
8.20
7.63
7.61
7.59
7.53
7.52
7.30
7.28
5.74
4.09
4.08
3.29
18b
(400 MHz, DMSO-d_6)
1.00
0.93
1.03
1.00
0.99
1.00
2.16
1.14

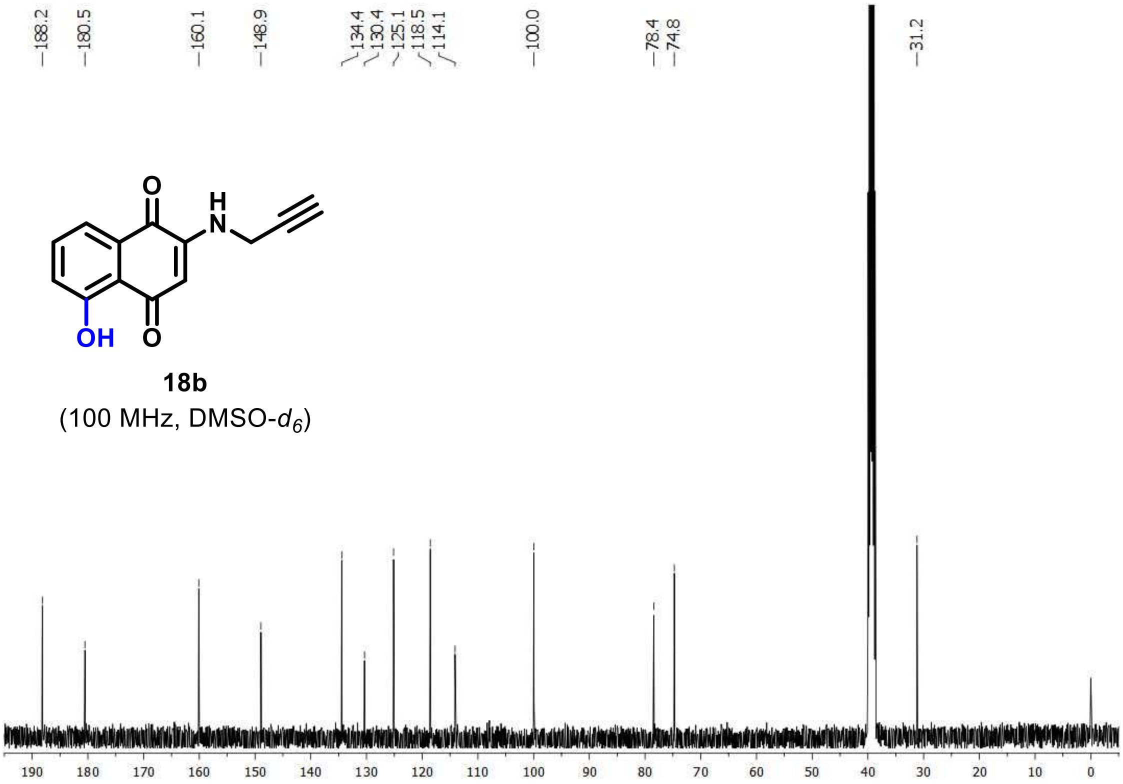
188.2
180.5
160.1
148.9
134.4
130.4
125.1
118.5
114.1
100.0
78.4
74.8
31.2
18b
(100 MHz, DMSO-d_6)

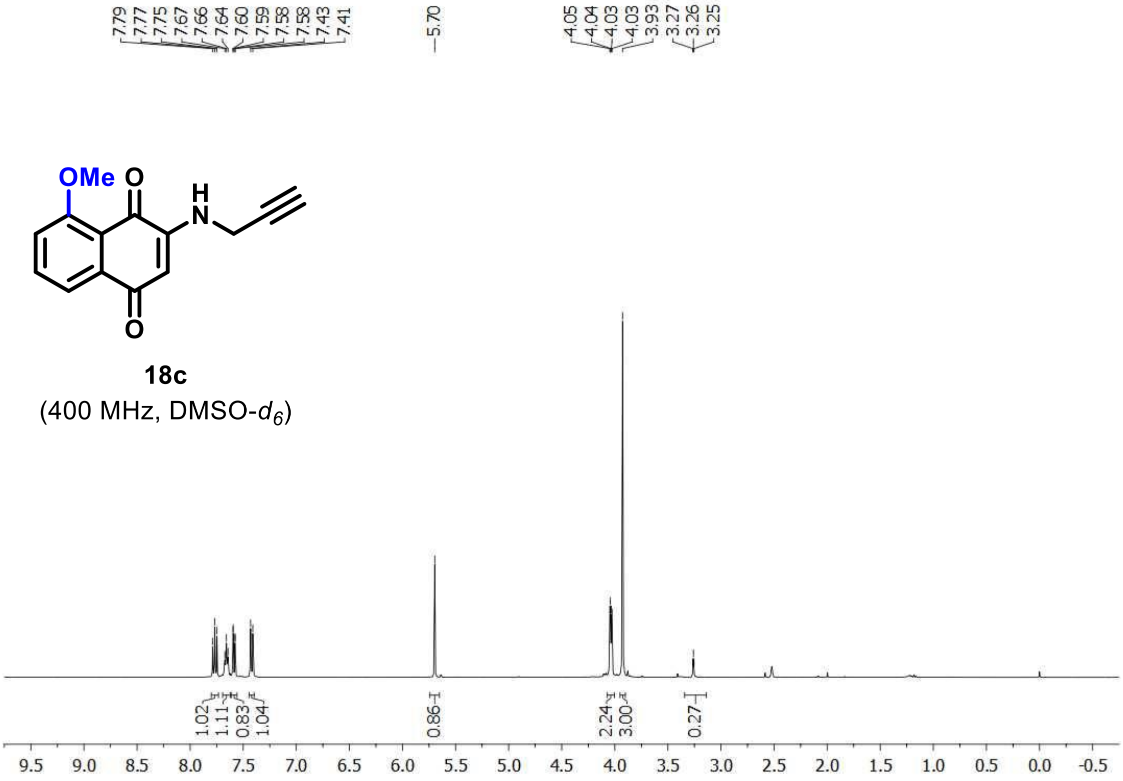
7.79
7.77
7.75
7.67
7.66
7.64
7.60
7.59
7.58
7.58
7.43
7.41
5.70
4.05
4.04
4.03
4.03
3.93
3.27
3.26
3.25
OMe O
H
N
O
18c
(400 MHz, DMSO-d6)
1.02
1.11
0.83
1.04
0.86
2.24
3.00
0.27
9.5 9.0 8.5 8.0 7.5 7.0 6.5 6.0 5.5 5.0 4.5 4.0 3.5 3.0 2.5 2.0 1.5 1.0 0.5 0.0 -0.5

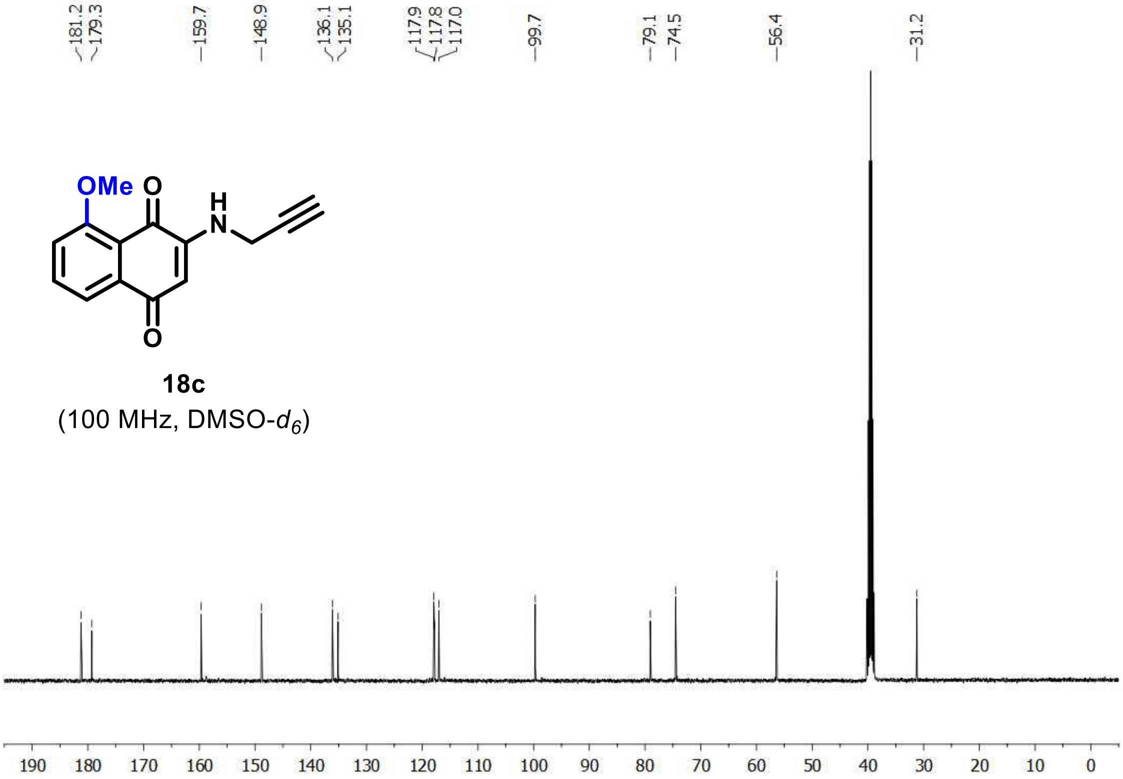
181.2
179.3
159.7
148.9
136.1
135.1
117.9
117.8
117.0
99.7
79.1
74.5
56.4
31.2
OMe O
H
N
O
18c
(100 MHz, DMSO-d6)
190 180 170 160 150 140 130 120 110 100 90 80 70 60 50 40 30 20 10 0

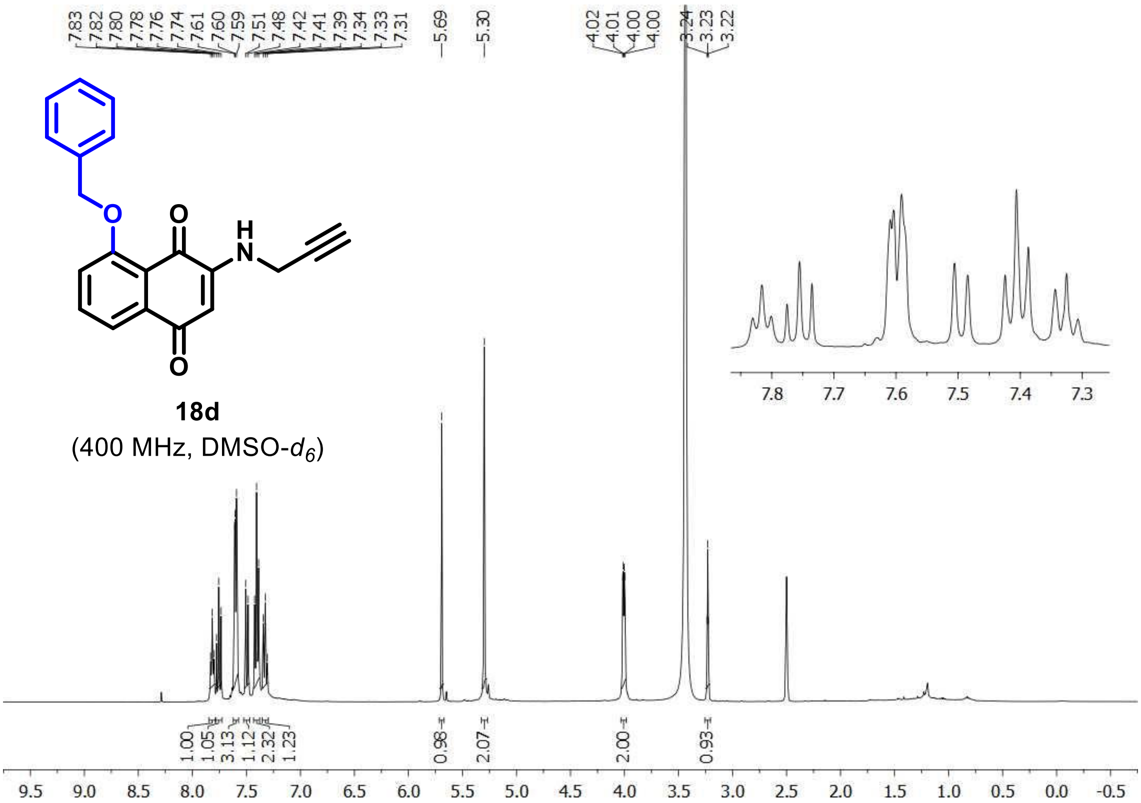
18d
(400 MHz, DMSO-d6)

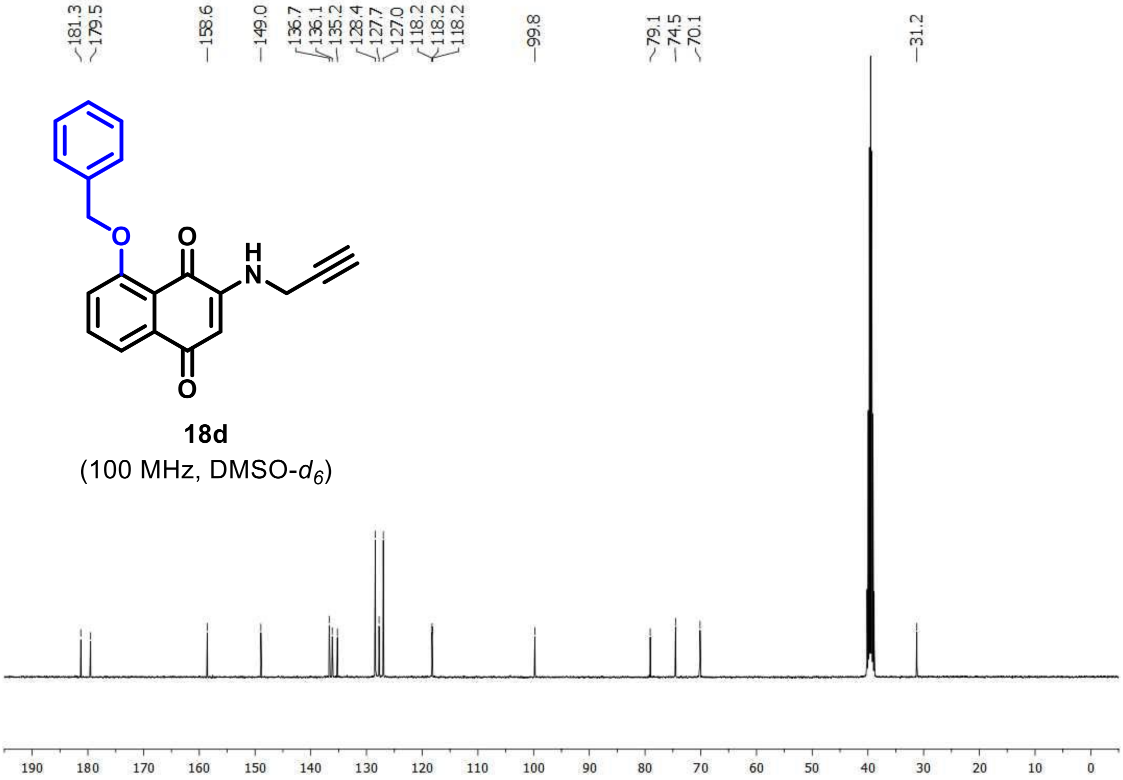
18d
(100 MHz, DMSO-d6)

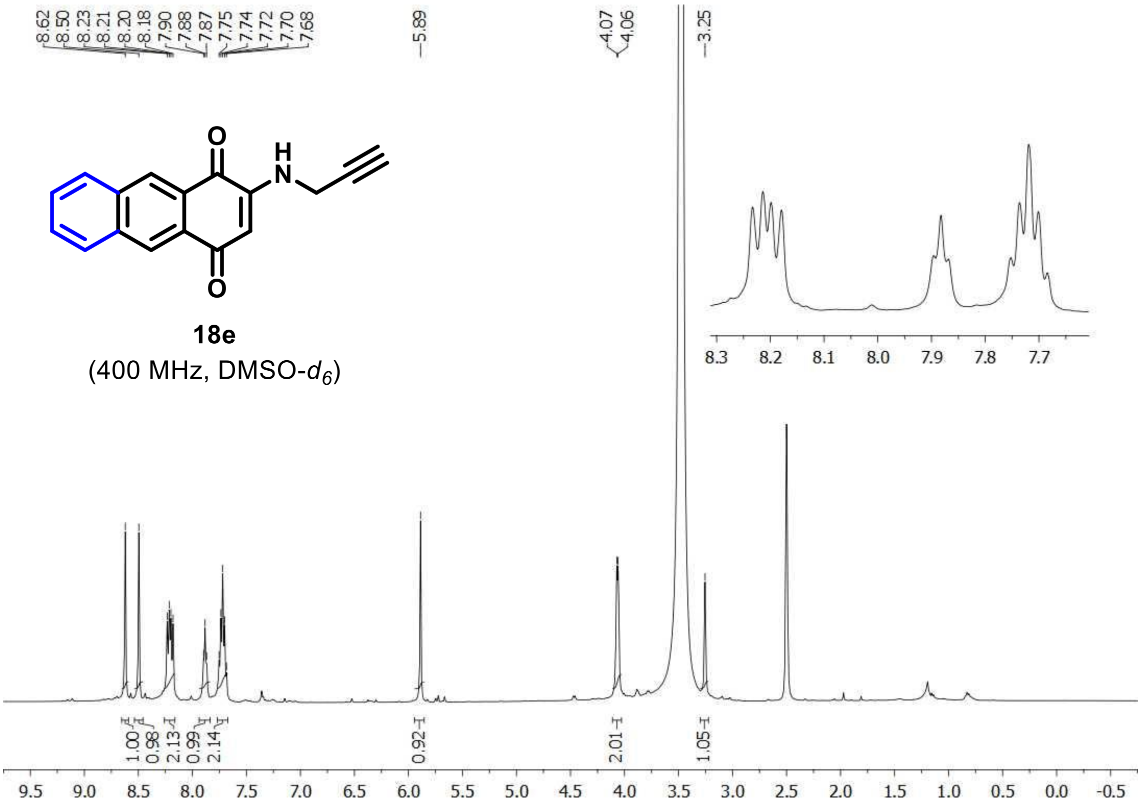

18e
(400 MHz, DMSO-d_6)

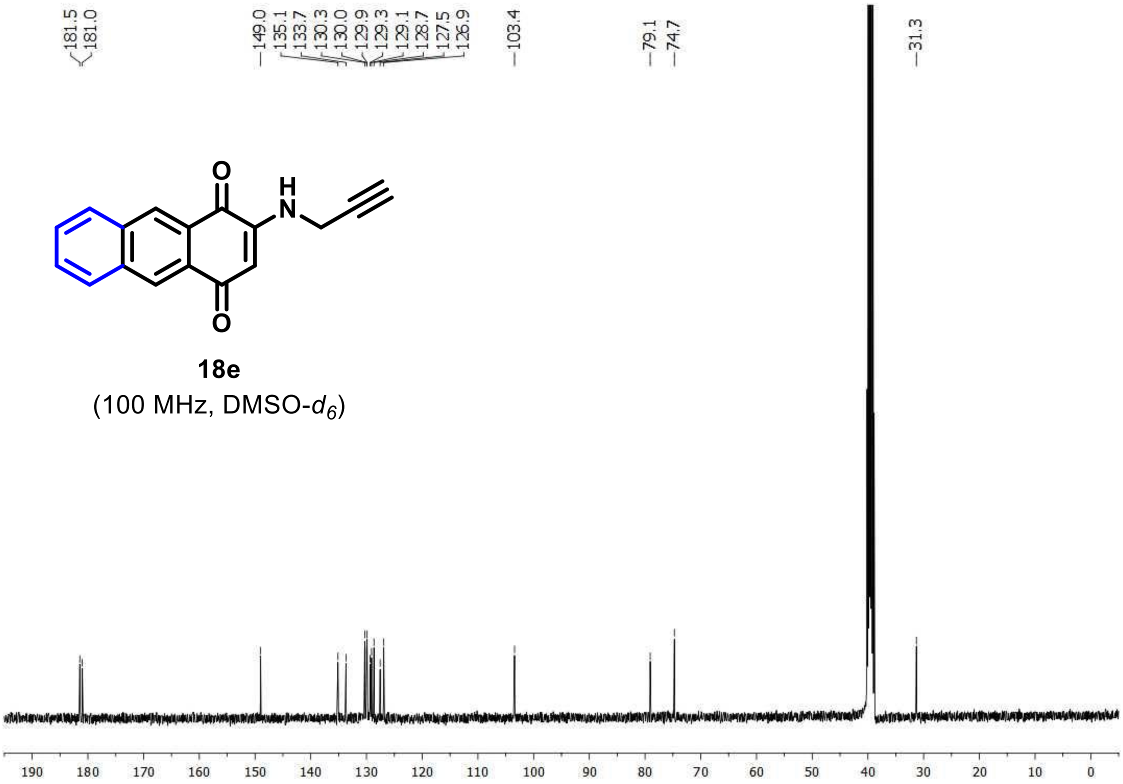

18e
(100 MHz, DMSO-d_6)

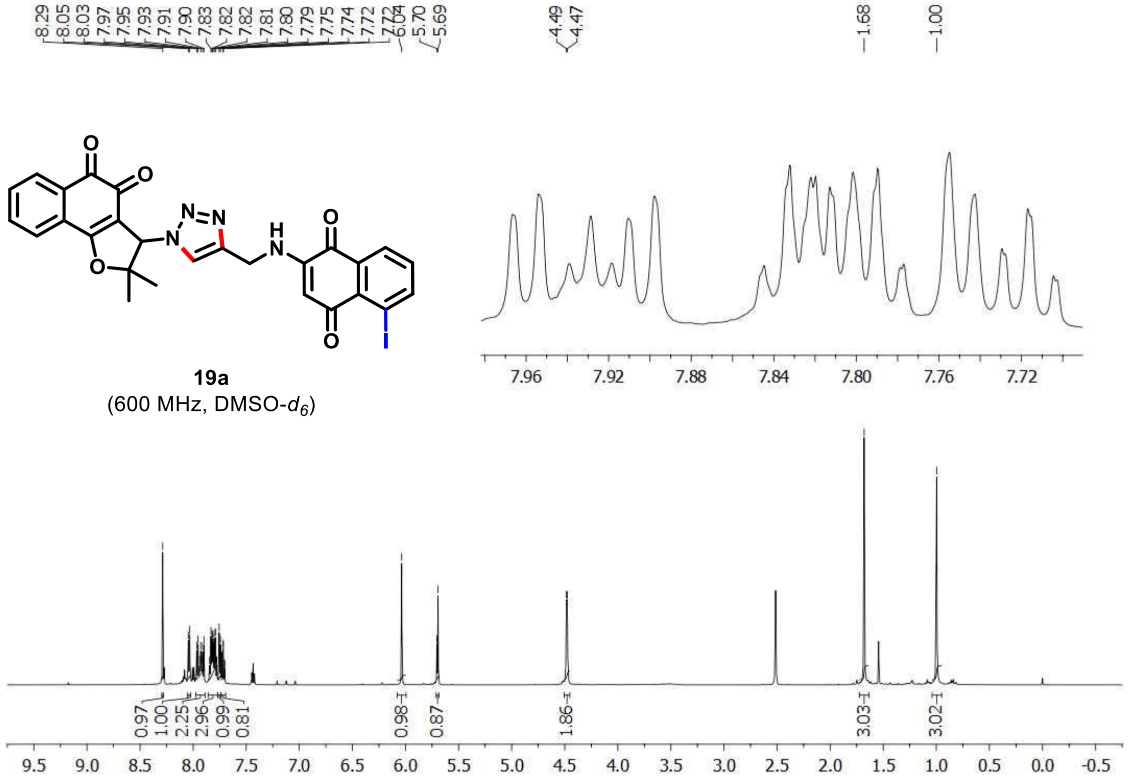
8.29 8.05 8.03 7.97 7.95 7.93 7.91 7.90 7.83 7.82 7.82 7.81 7.80 7.79 7.75 7.74 7.72 7.72 6.04 5.70 5.69
4.49 4.47
1.68
1.00
19a
(600 MHz, DMSO-d_6)
0.97 1.00 2.25 2.96 0.99 0.81
0.98 0.87
1.86
3.03
3.02

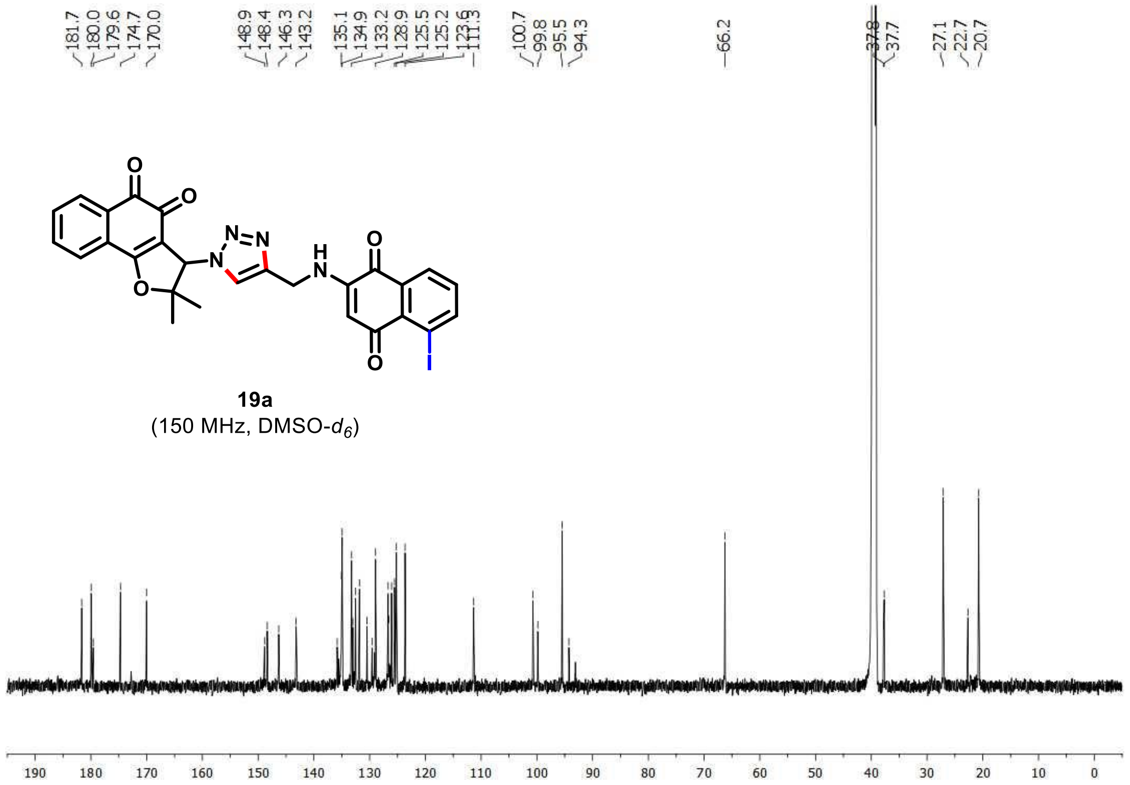
181.7 180.0 179.6 174.7 170.0
148.9 148.4 146.3 143.2
135.1 134.9 133.2 128.9 125.5 125.2 123.6 111.3
100.7 99.8 95.5 94.3
66.2
37.8 37.7
27.1 22.7 20.7
19a
(150 MHz, DMSO-d_6)

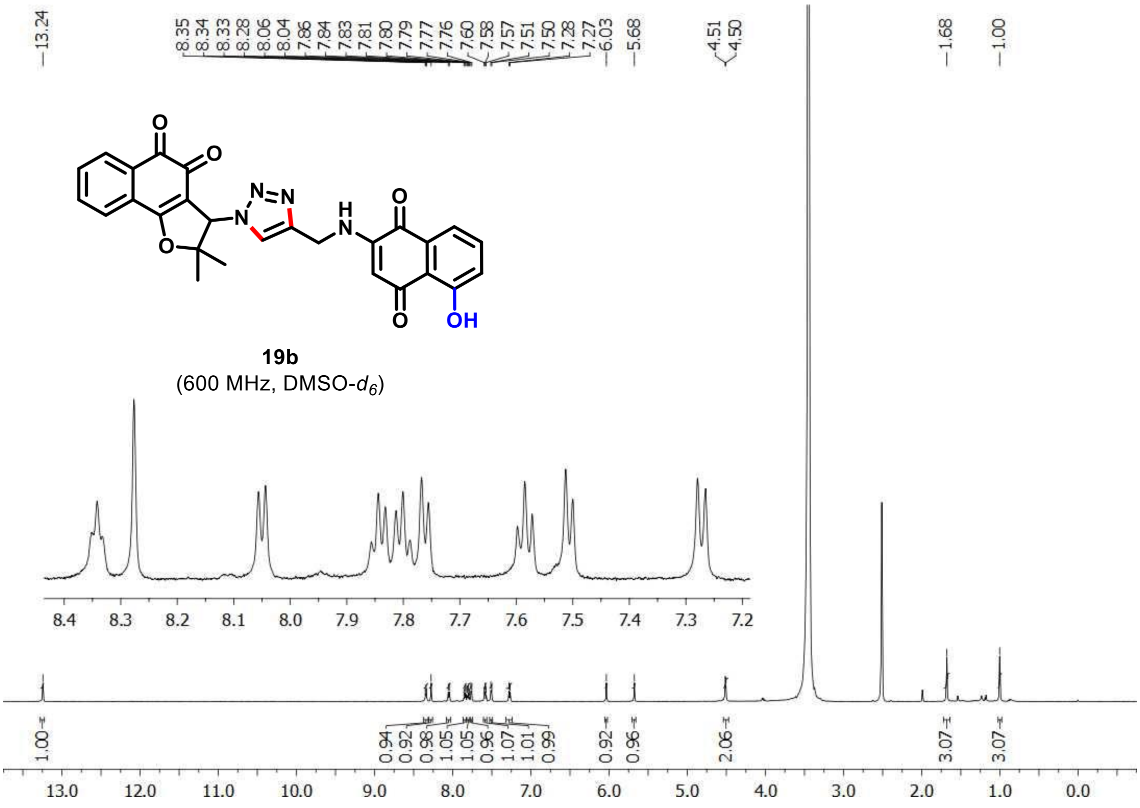
19b
(600 MHz, DMSO-d_6)

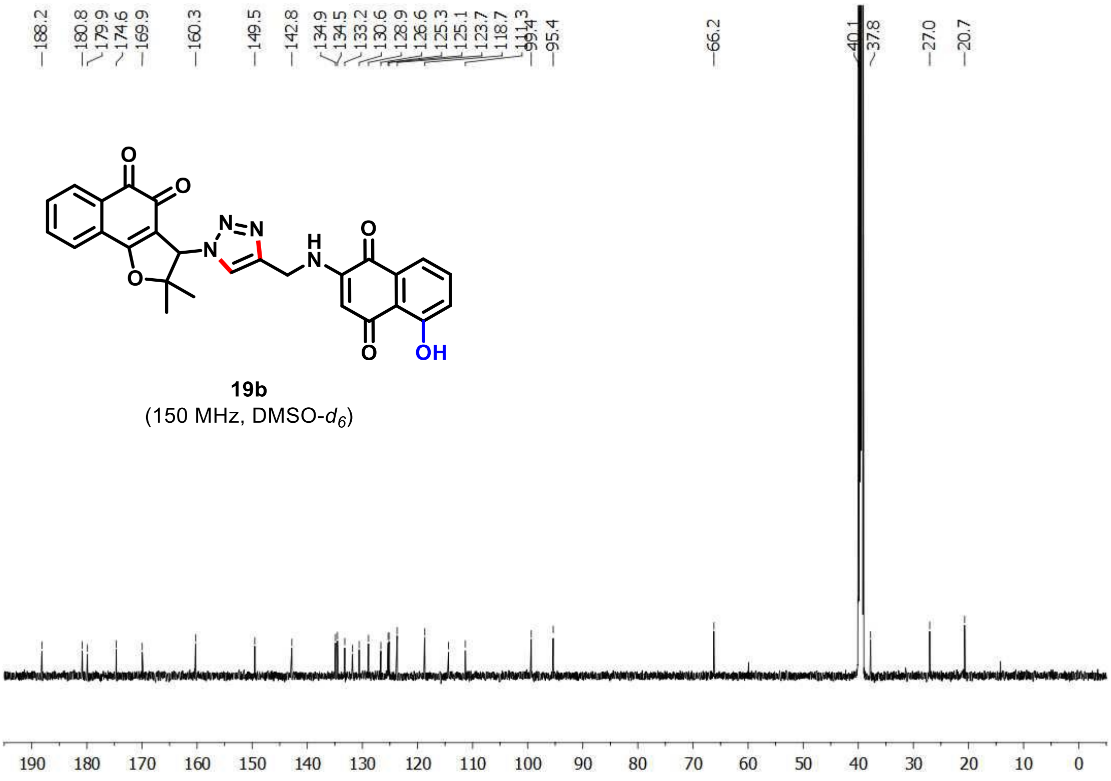
19b
(150 MHz, DMSO-d_6)

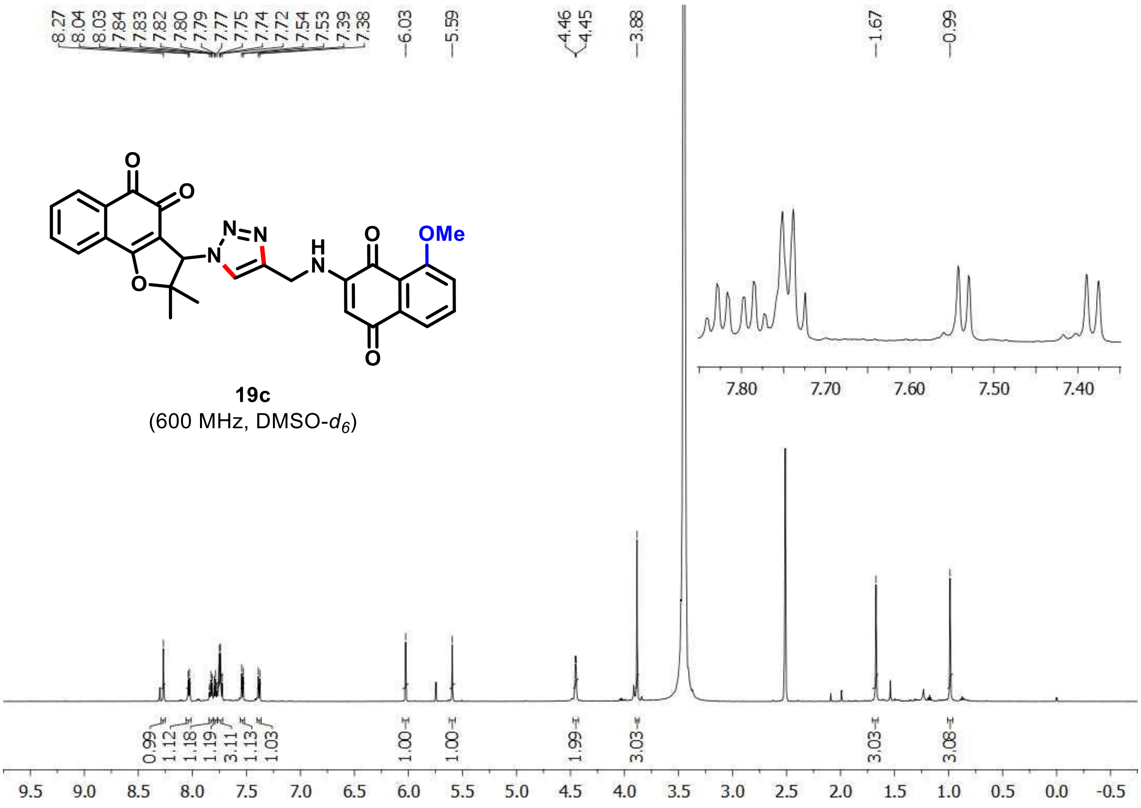
8.27
8.04
8.03
7.84
7.83
7.82
7.80
7.79
7.77
7.75
7.74
7.72
7.54
7.53
7.39
7.38
6.03
5.59
4.46
4.45
3.88
1.67
0.99
O
O
N
N
N
H
N
O
OMe
O
19c
(600 MHz, DMSO-d_6)
0.99
1.12
1.18
1.19
3.11
1.13
1.03
1.00
1.00
1.99
3.03
3.03
3.08

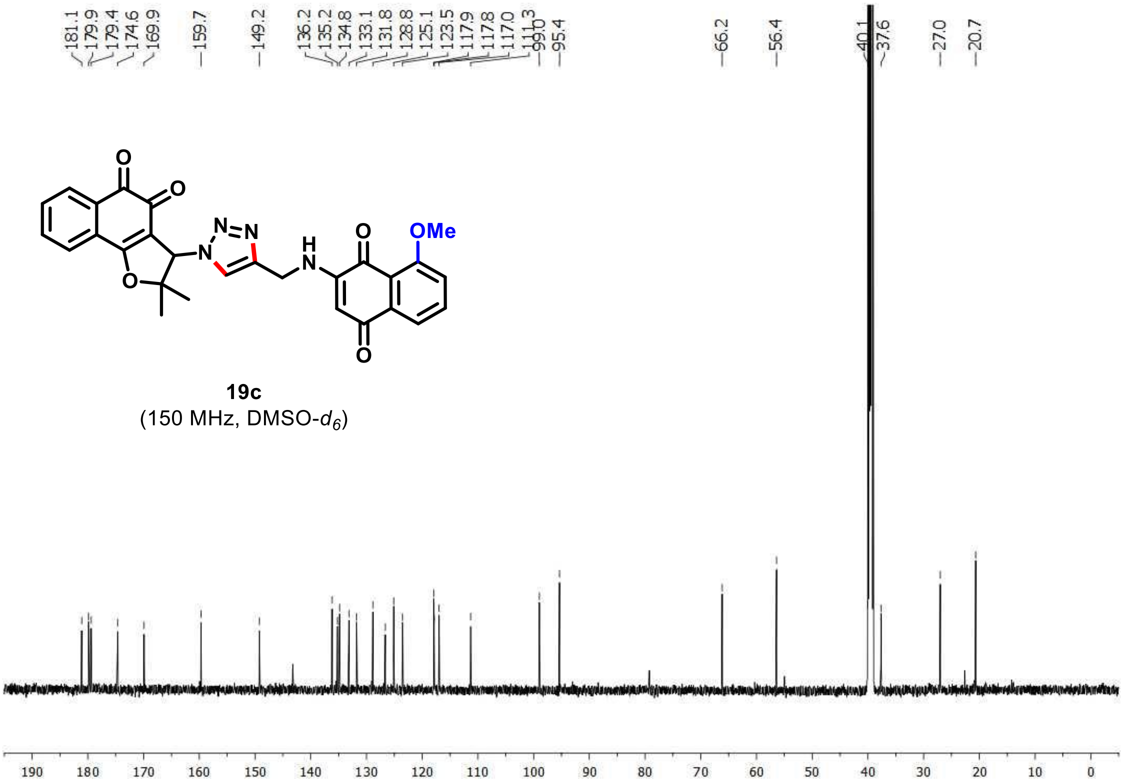
181.1
179.9
179.4
174.6
169.9
159.7
149.2
136.2
135.2
134.8
133.1
131.8
128.8
125.1
123.5
117.9
117.8
117.0
111.3
99.0
95.4
66.2
56.4
40.1
37.6
27.0
20.7
19c
(150 MHz, DMSO-d_6)

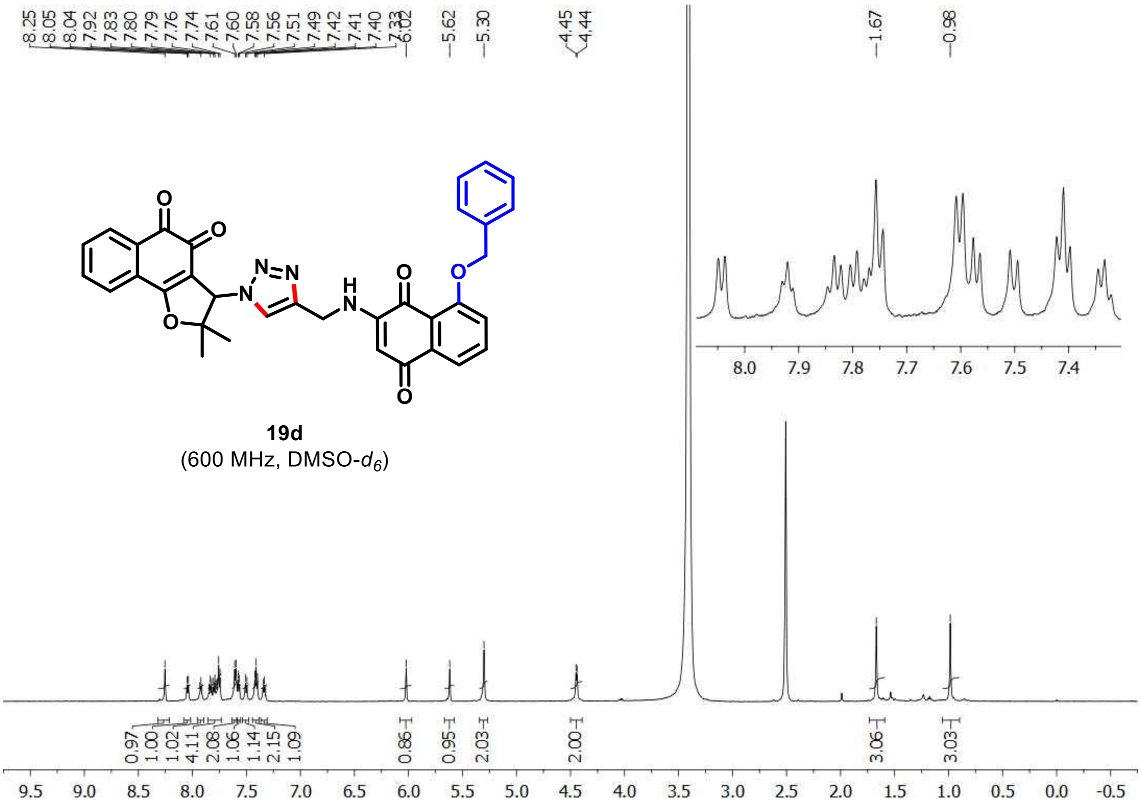
19d
(600 MHz, DMSO-d6)

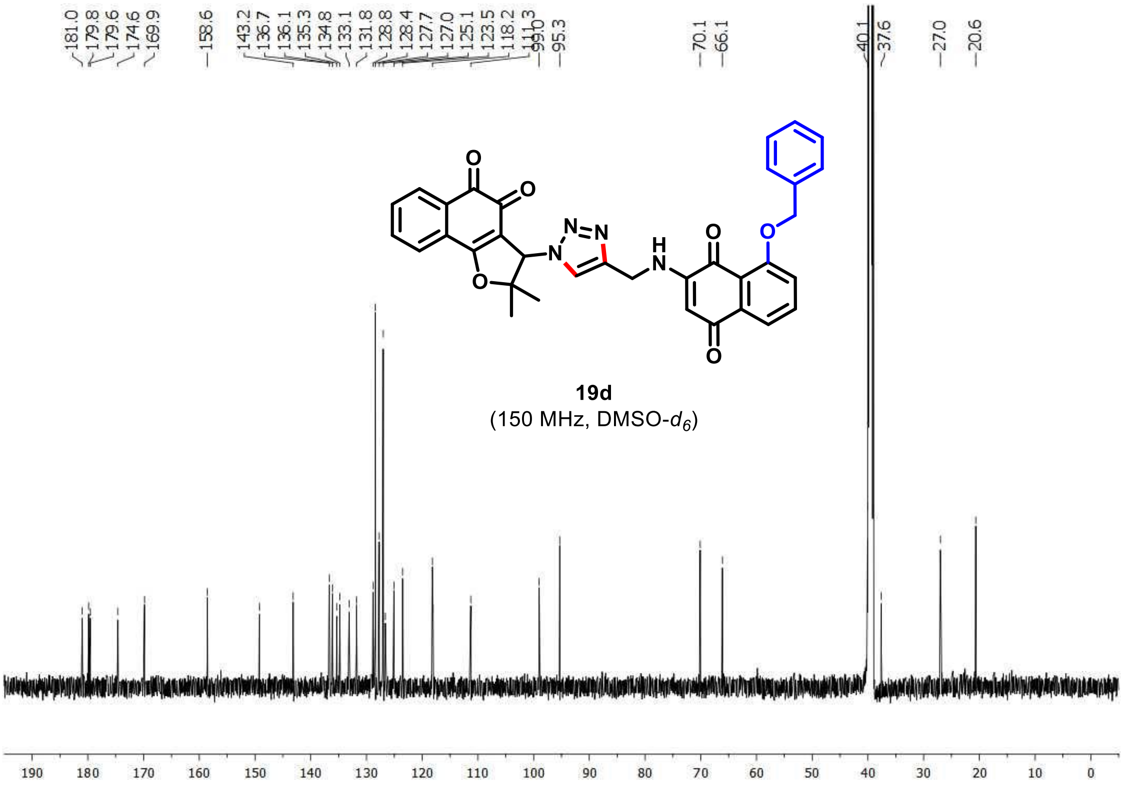
19d
(150 MHz, DMSO-d6)

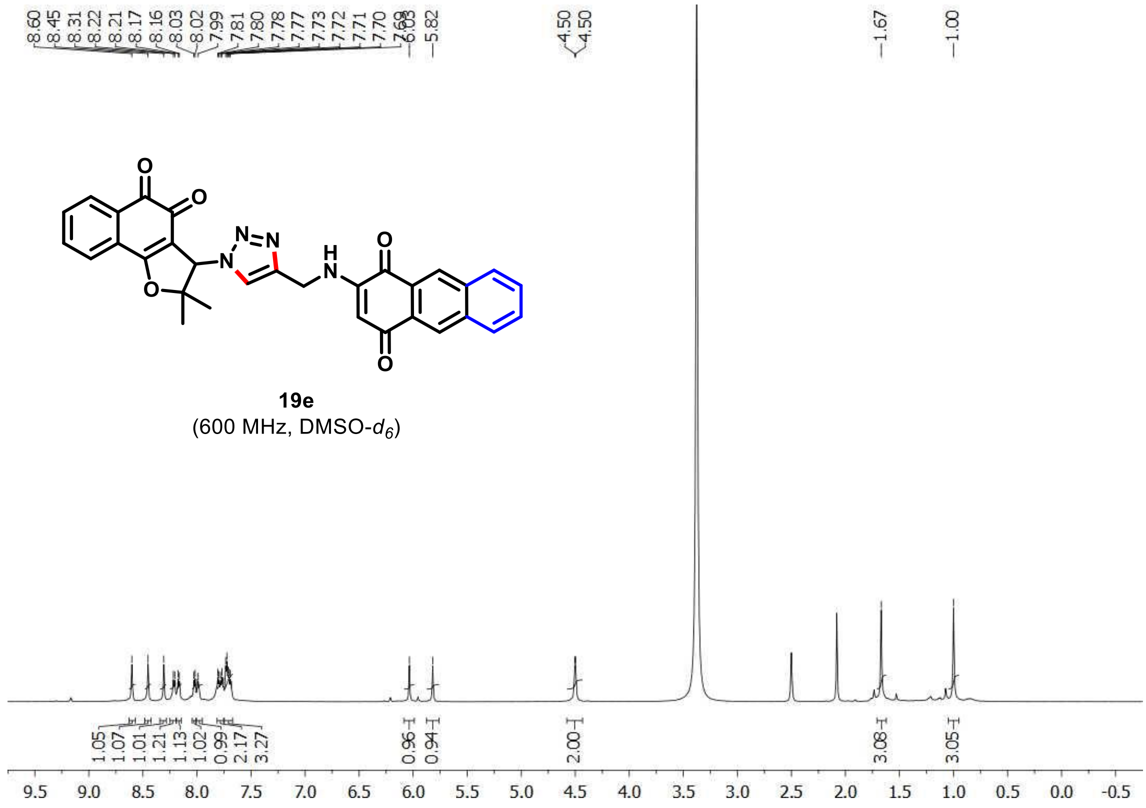
19e
(600 MHz, DMSO-d6)

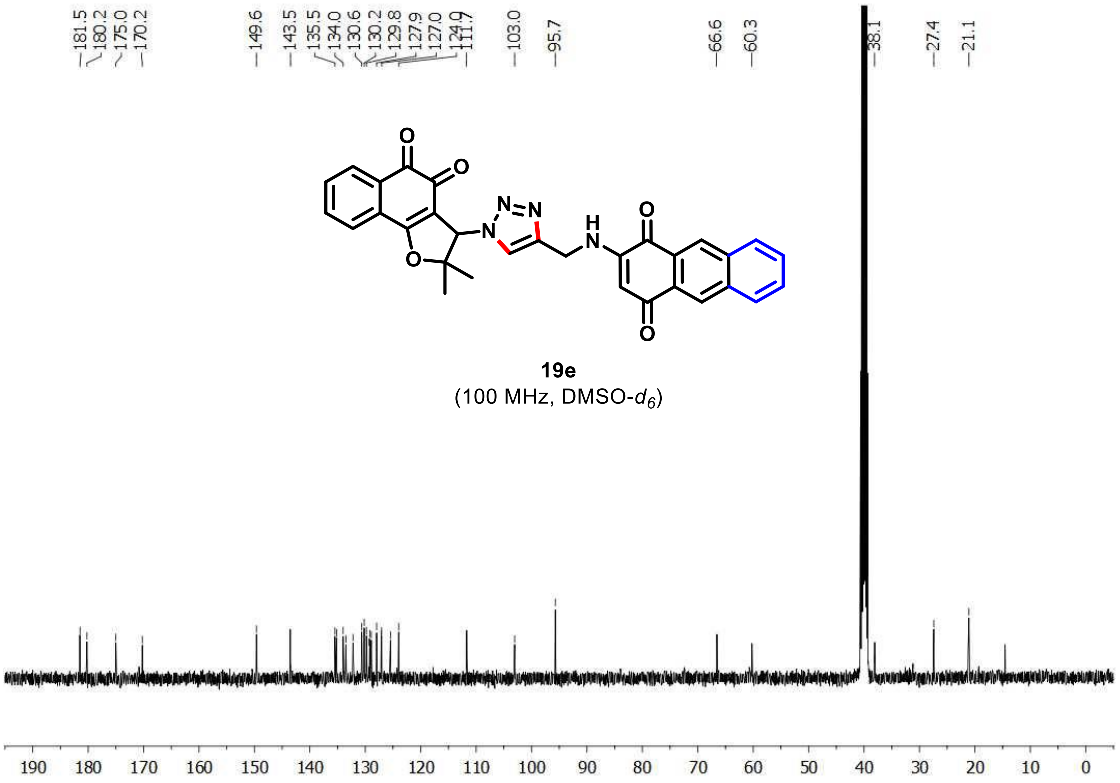
19e
(100 MHz, DMSO-d6)

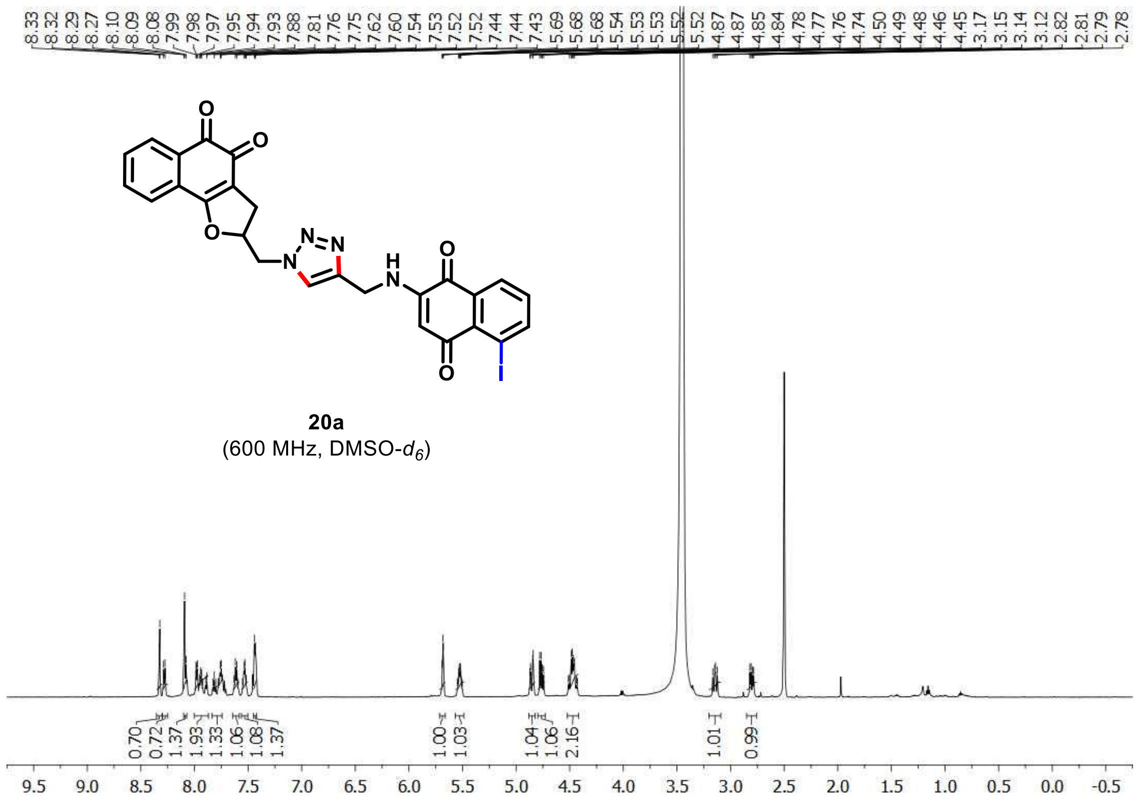
8.33
8.32
8.29
8.27
8.10
8.09
8.08
7.99
7.98
7.97
7.95
7.94
7.93
7.88
7.81
7.76
7.75
7.62
7.60
7.54
7.53
7.52
7.52
7.44
7.44
7.43
5.69
5.68
5.68
5.54
5.53
5.53
5.52
5.52
4.87
4.87
4.85
4.84
4.78
4.77
4.76
4.74
4.50
4.49
4.48
4.46
4.45
3.17
3.15
3.14
3.12
2.82
2.81
2.79
2.78
O
O
O
N
N
N
N
H
N
O
O
I
20a
(600 MHz, DMSO-d_6)
0.70
0.72
1.37
1.93
1.33
1.06
1.08
1.37
1.00
1.03
1.04
1.06
2.16
1.01
0.99
9.5
9.0
8.5
8.0
7.5
7.0
6.5
6.0
5.5
5.0
4.5
4.0
3.5
3.0
2.5
2.0
1.5
1.0
0.5
0.0
-0.5

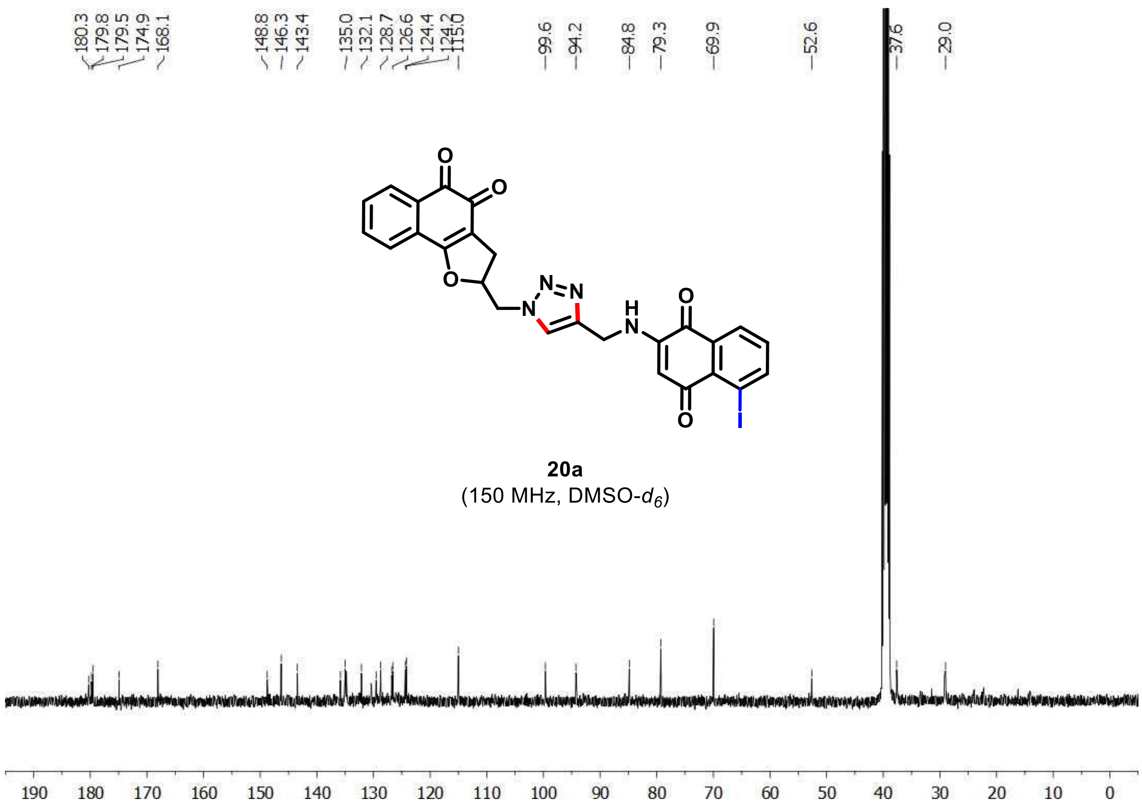
180.3
179.8
179.5
174.9
168.1
148.8
146.3
143.4
135.0
132.1
128.7
126.6
124.4
124.2
115.0
99.6
94.2
84.8
79.3
69.9
52.6
37.6
29.0
O
O
O
N
N
N
N
H
N
O
O
I
20a
(150 MHz, DMSO-d_6)
190
180
170
160
150
140
130
120
110
100
90
80
70
60
50
40
30
20
10
0

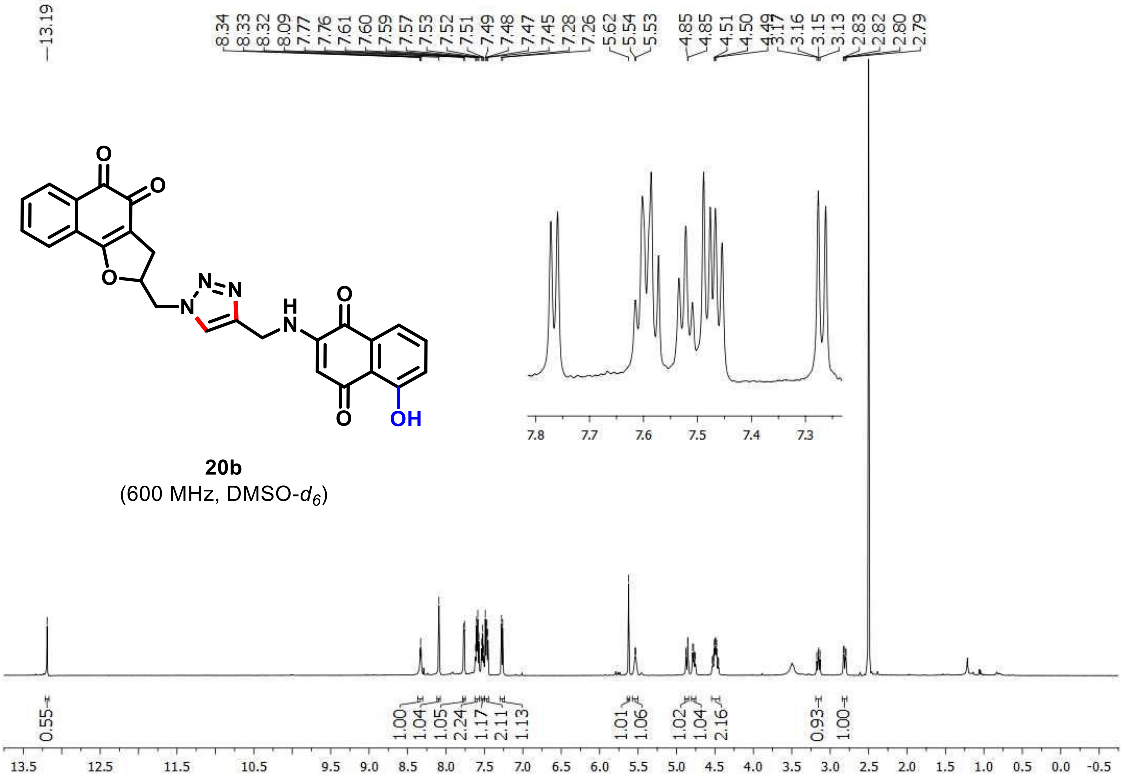

20b
(600 MHz, DMSO-d_6)

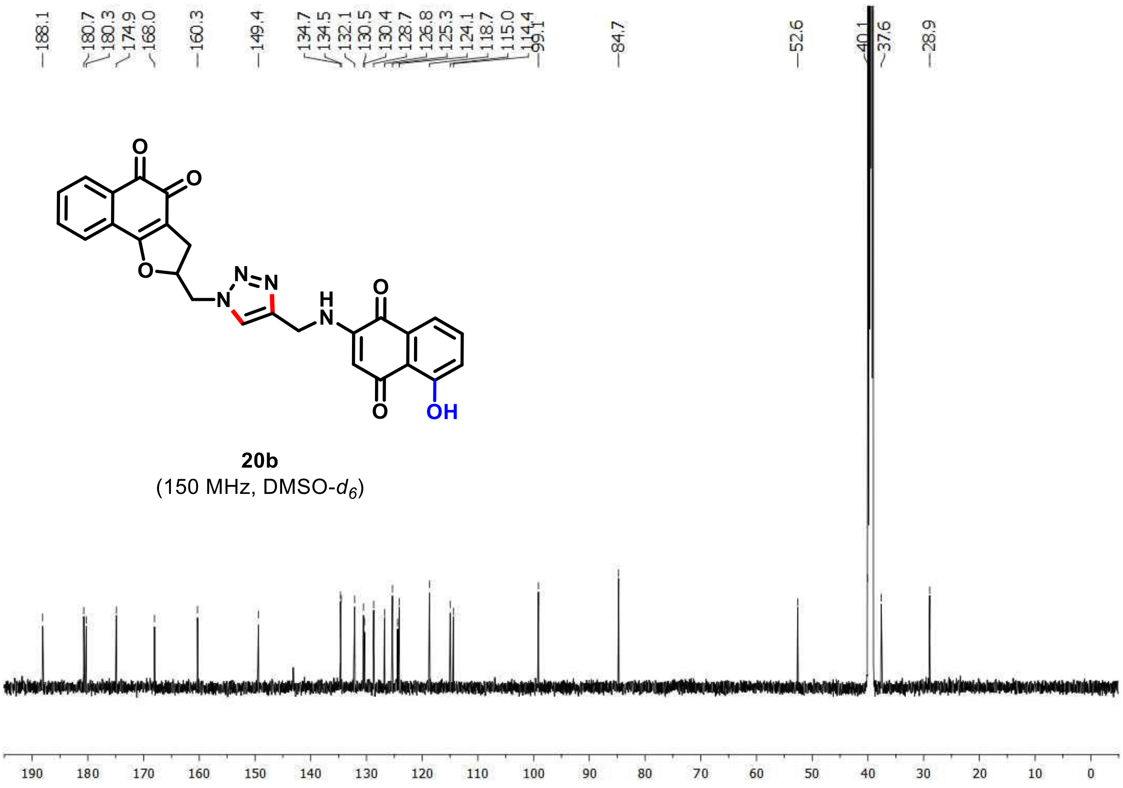

20b
(150 MHz, DMSO-d_6)

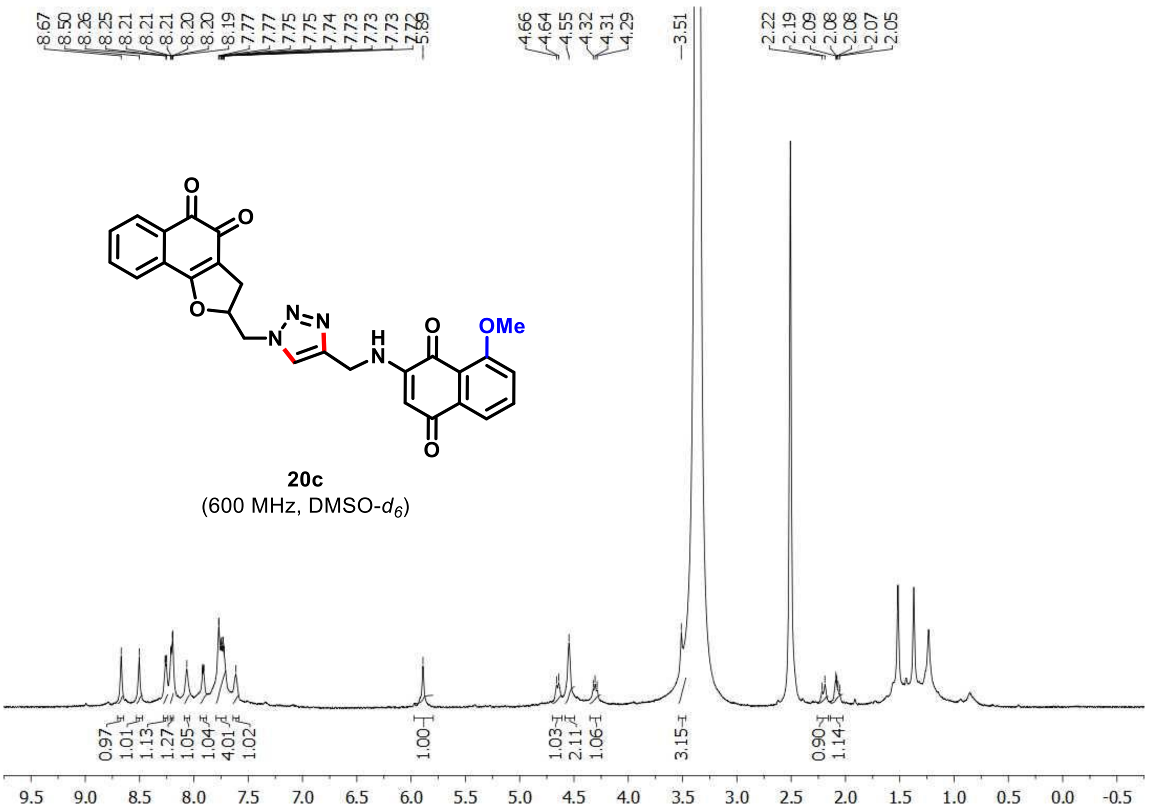
8.67
8.50
8.26
8.25
8.21
8.21
8.21
8.20
8.20
8.19
7.77
7.77
7.75
7.75
7.74
7.73
7.73
7.73
7.73
7.72
5.89
4.66
4.64
4.55
4.32
4.31
4.29
3.51
2.22
2.19
2.09
2.08
2.08
2.07
2.05
OMe
20c
(600 MHz, DMSO-d_6)
0.97
1.01
1.13
1.27
1.05
1.04
4.01
1.02
1.00
1.03
2.11
1.06
3.15
0.90
1.14
9.5 9.0 8.5 8.0 7.5 7.0 6.5 6.0 5.5 5.0 4.5 4.0 3.5 3.0 2.5 2.0 1.5 1.0 0.5 0.0 -0.5

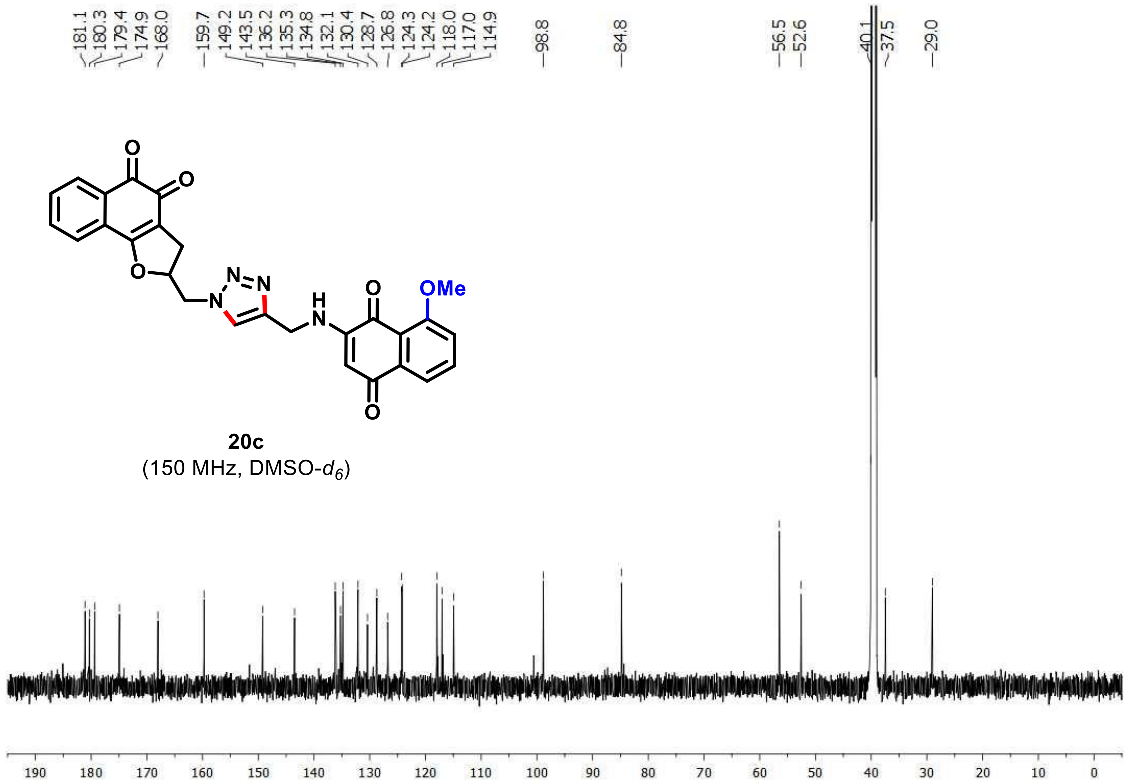
181.1
180.3
179.4
174.9
168.0
159.7
149.2
143.5
136.2
135.3
134.8
132.1
130.4
128.7
126.8
124.3
124.2
118.0
117.0
114.9
98.8
84.8
56.5
52.6
40.1
37.5
29.0
OMe
20c
(150 MHz, DMSO-d_6)
190 180 170 160 150 140 130 120 110 100 90 80 70 60 50 40 30 20 10 0

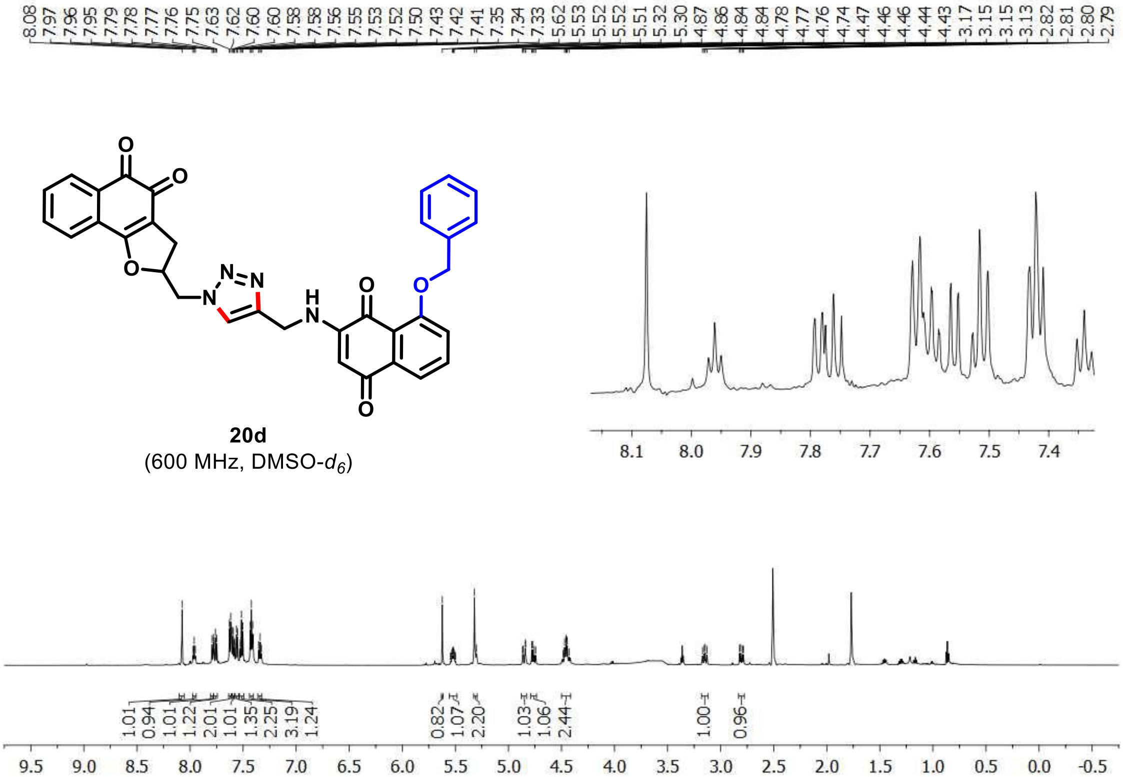

20d
(600 MHz, DMSO-d_6)

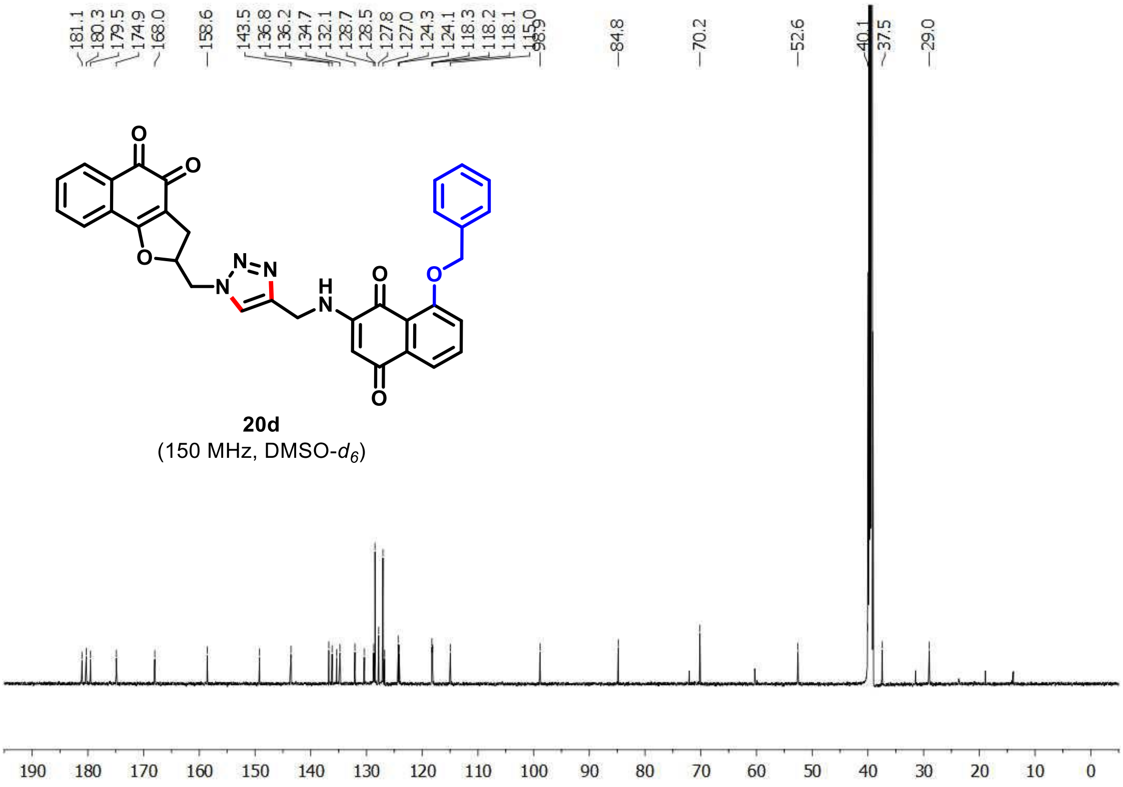

20d
(150 MHz, DMSO-d_6)

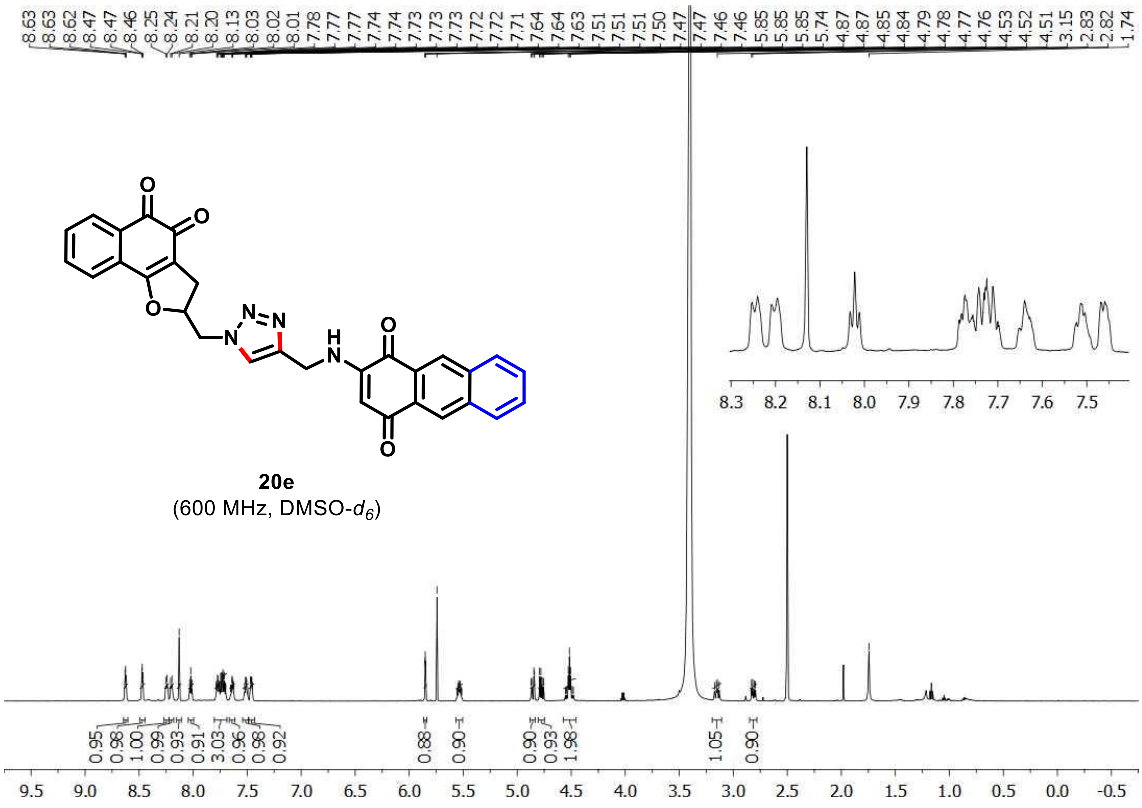

20e
(600 MHz, DMSO-d_6)

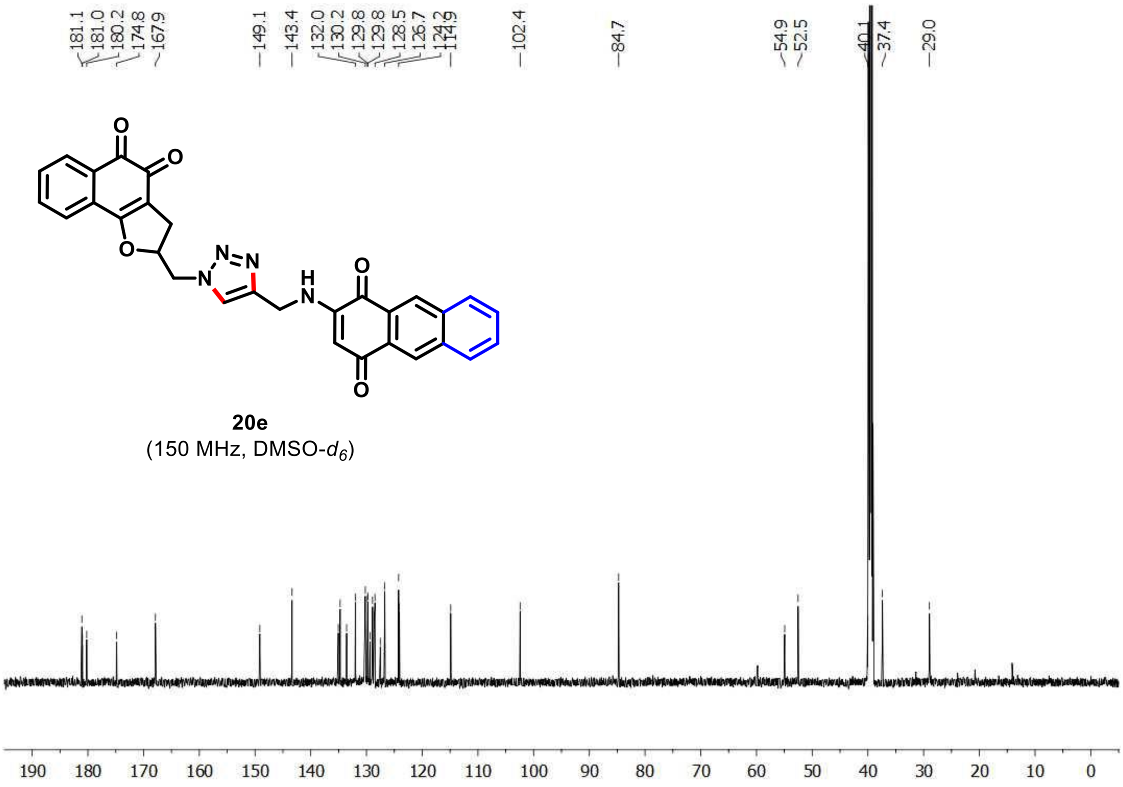

20e
(150 MHz, DMSO-d_6)

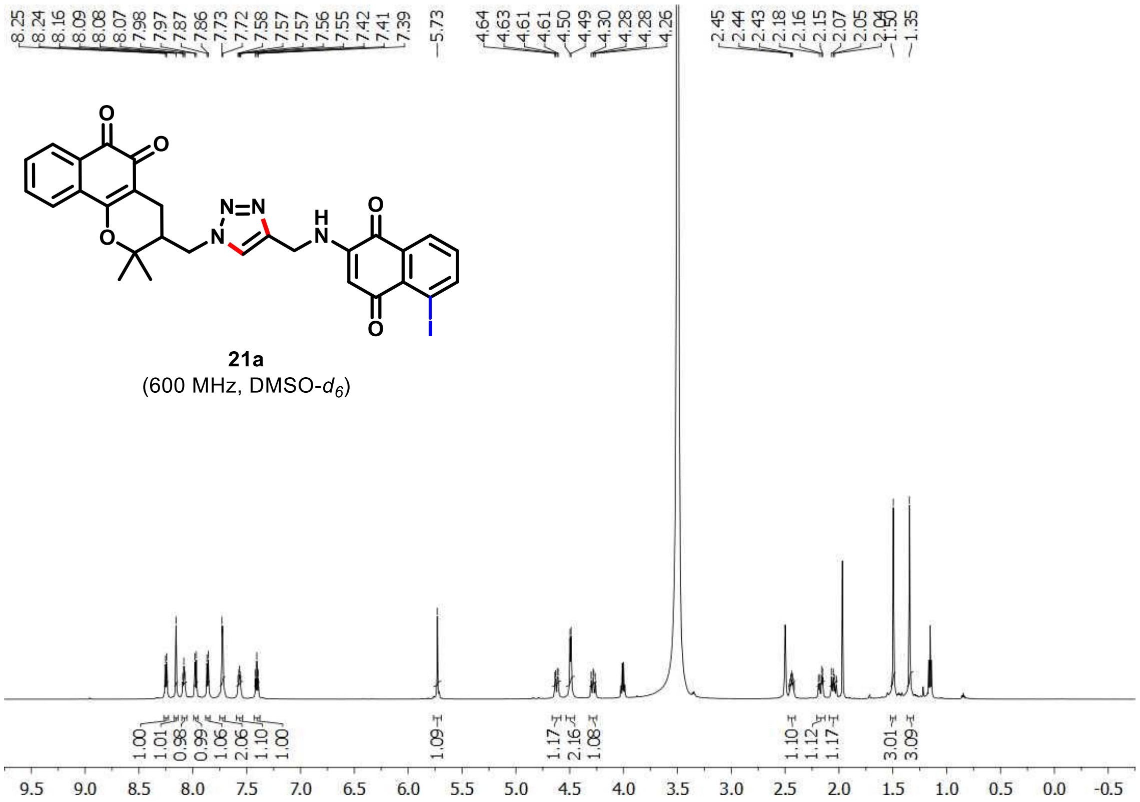

21a
(600 MHz, DMSO-d_6)

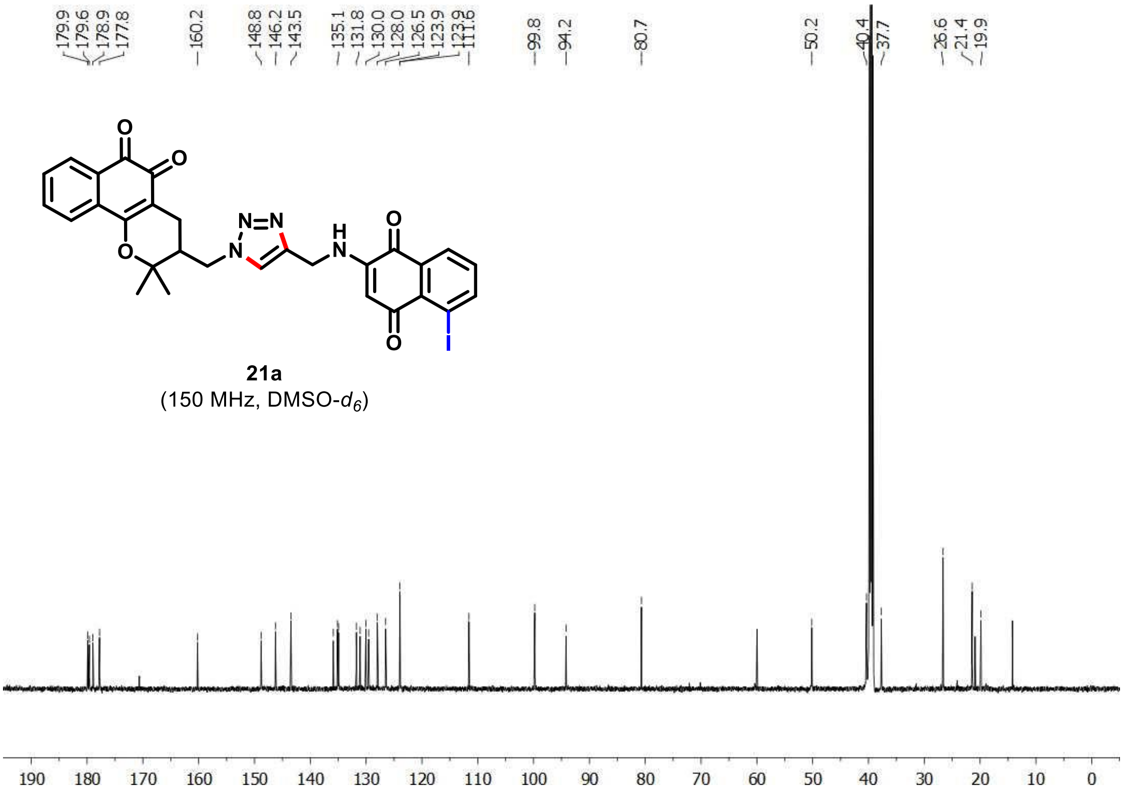

21a
(150 MHz, DMSO-d_6)

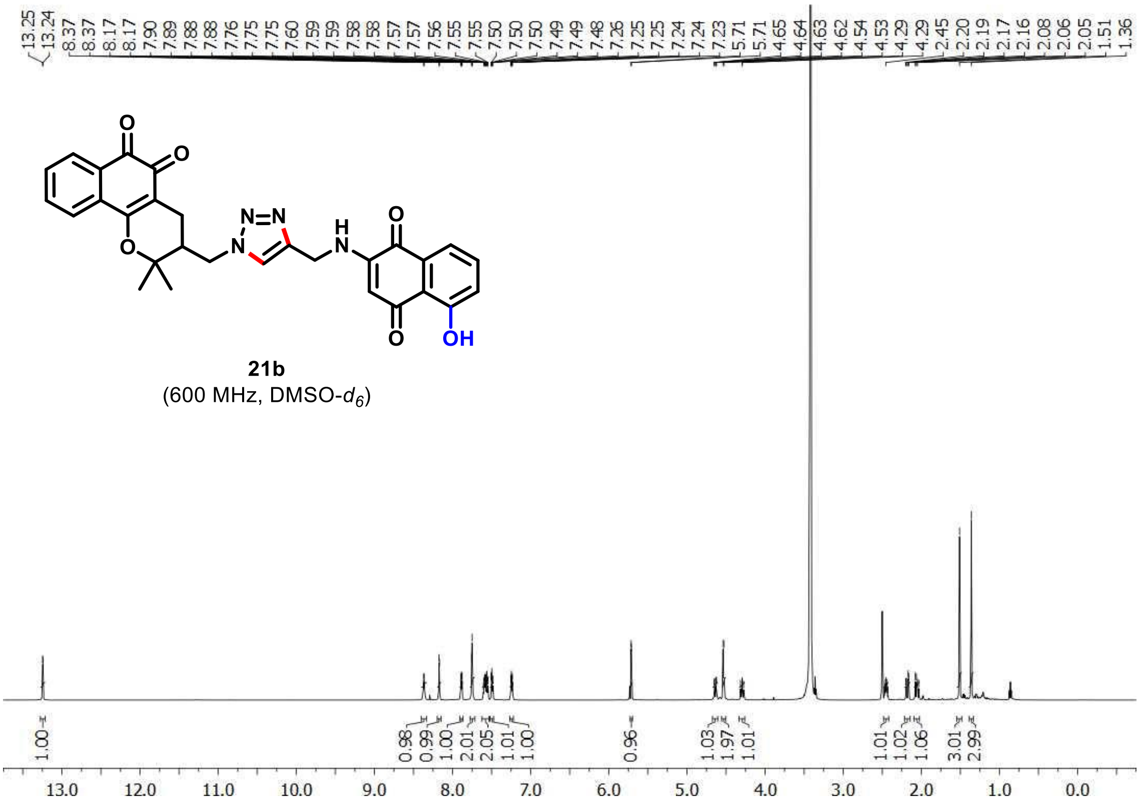

O
N=N
N
H
N
OH
21b
(600 MHz, DMSO-d_6)

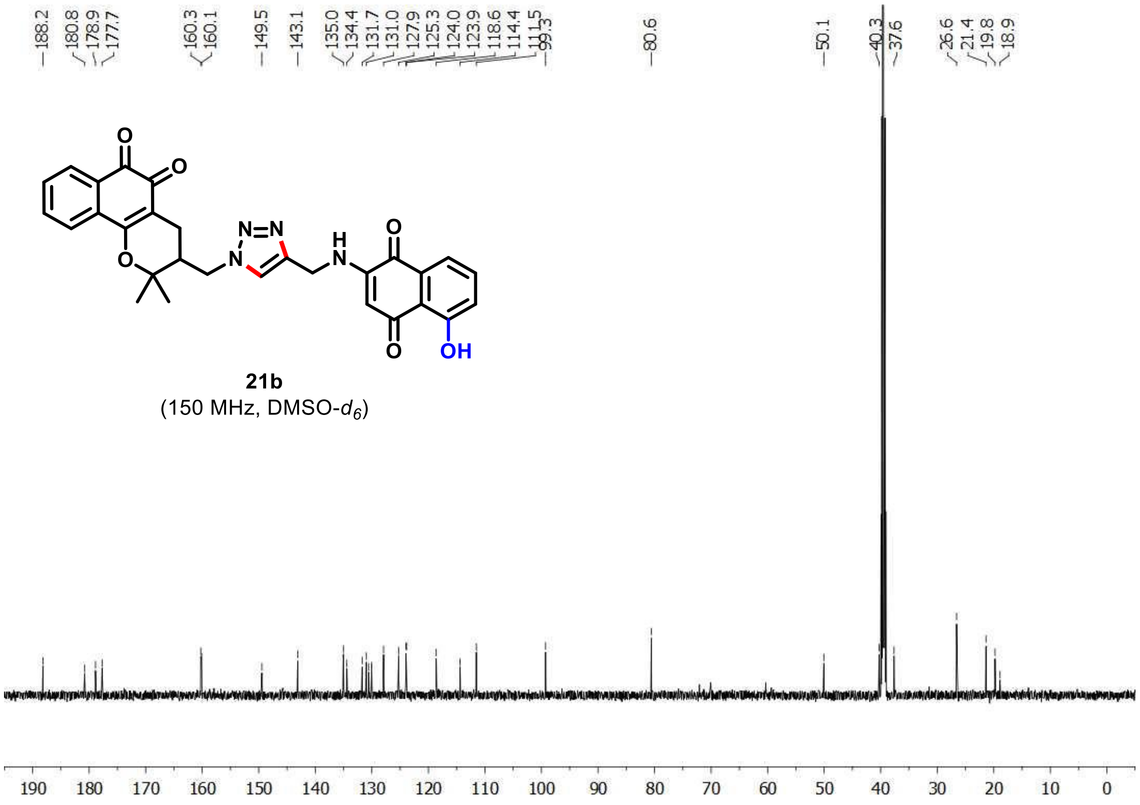

O
N=N
N
H
N
OH
21b
(150 MHz, DMSO-d_6)

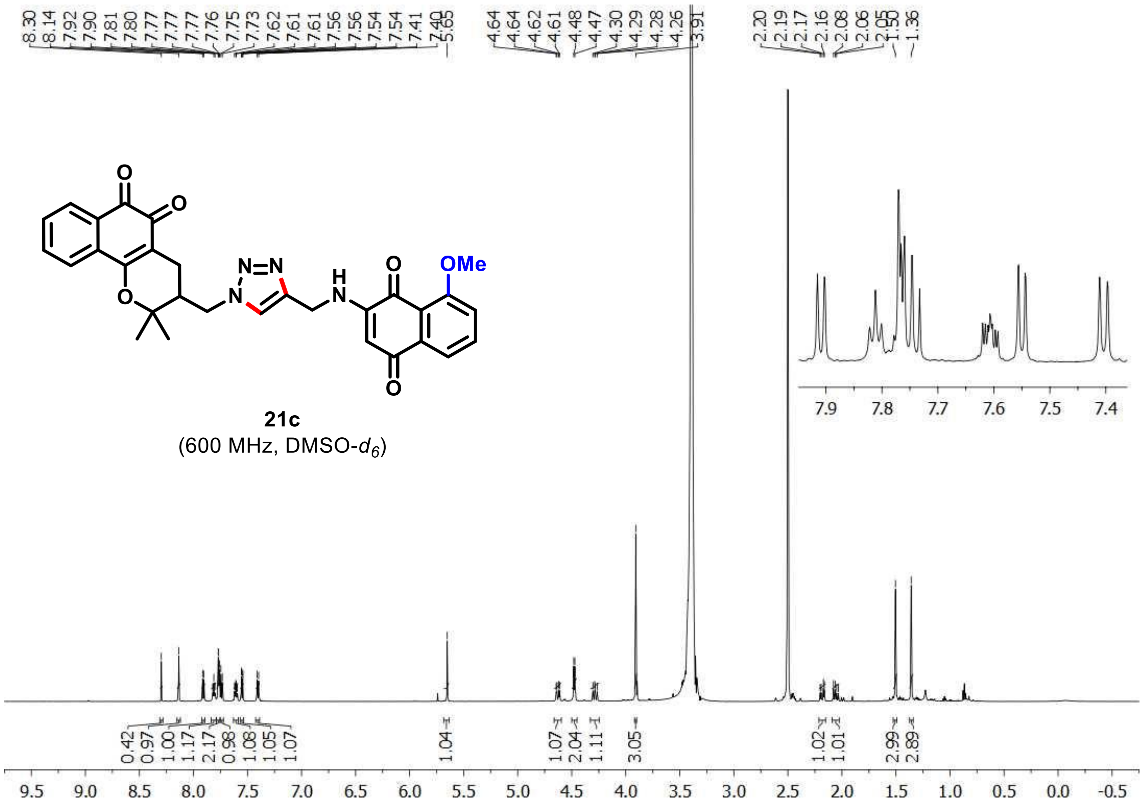
OMe
21c
(600 MHz, DMSO-d_6)

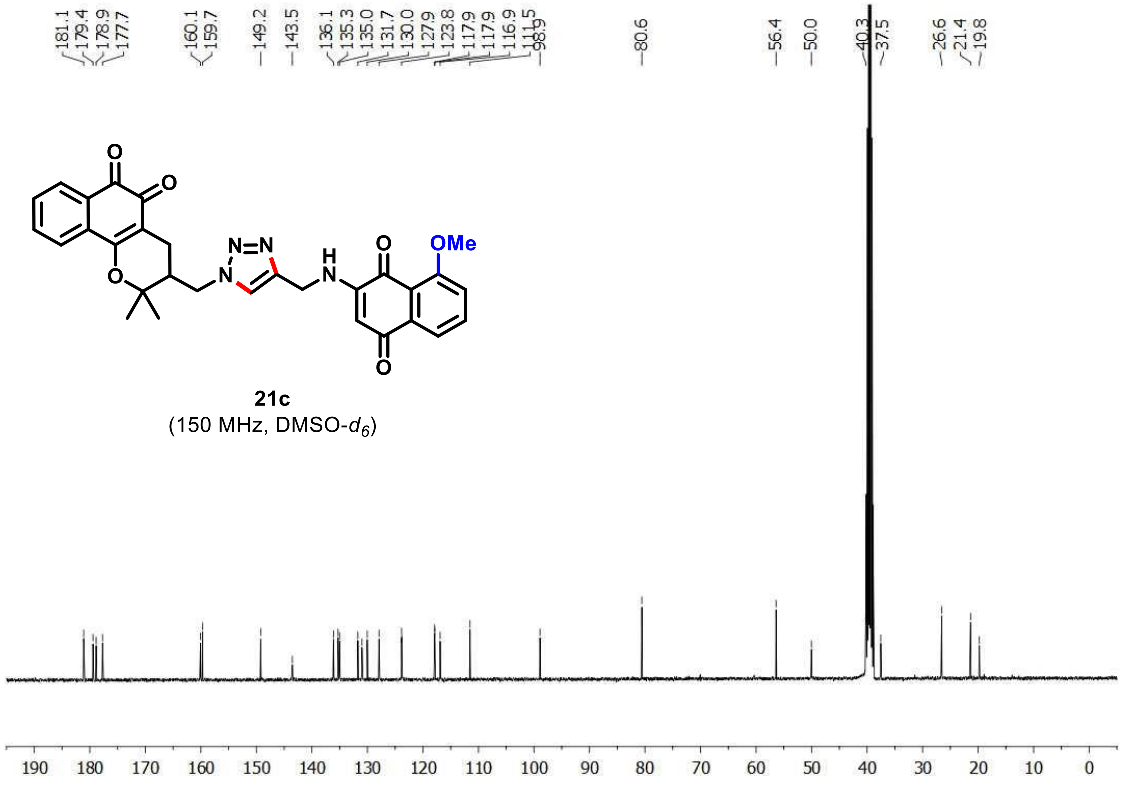
OMe
21c
(150 MHz, DMSO-d_6)

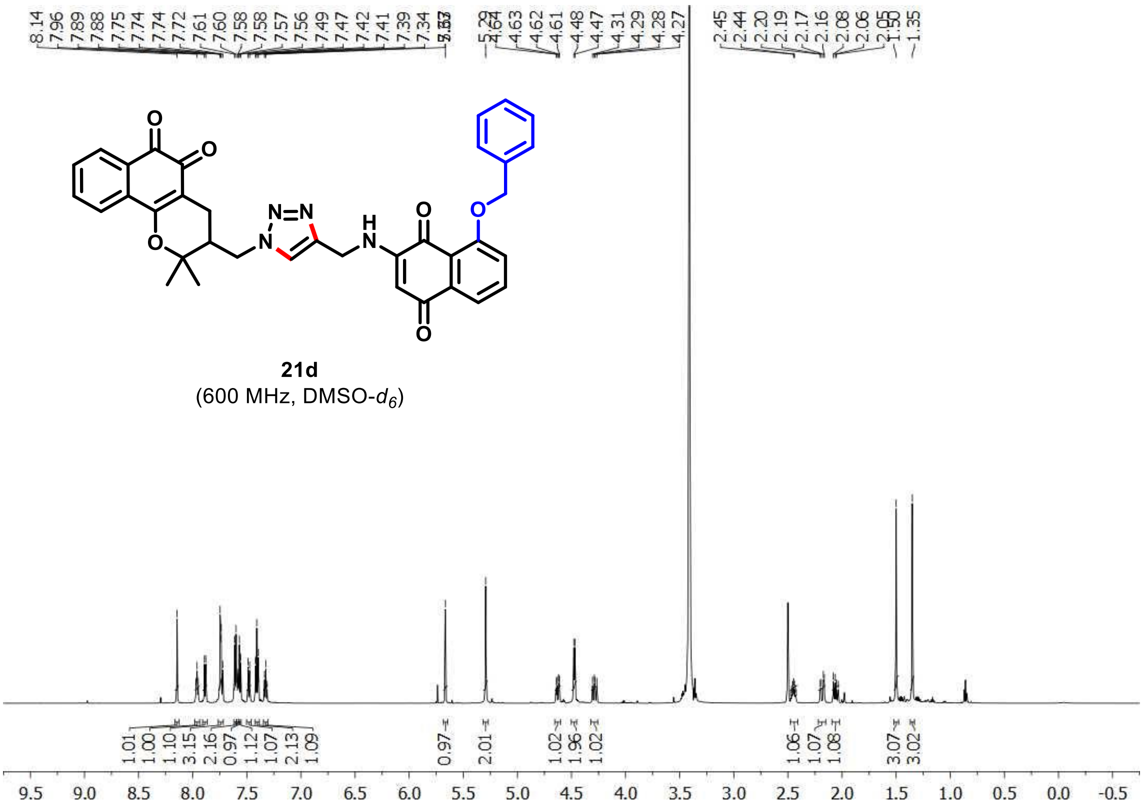
21d
(600 MHz, DMSO-d6)

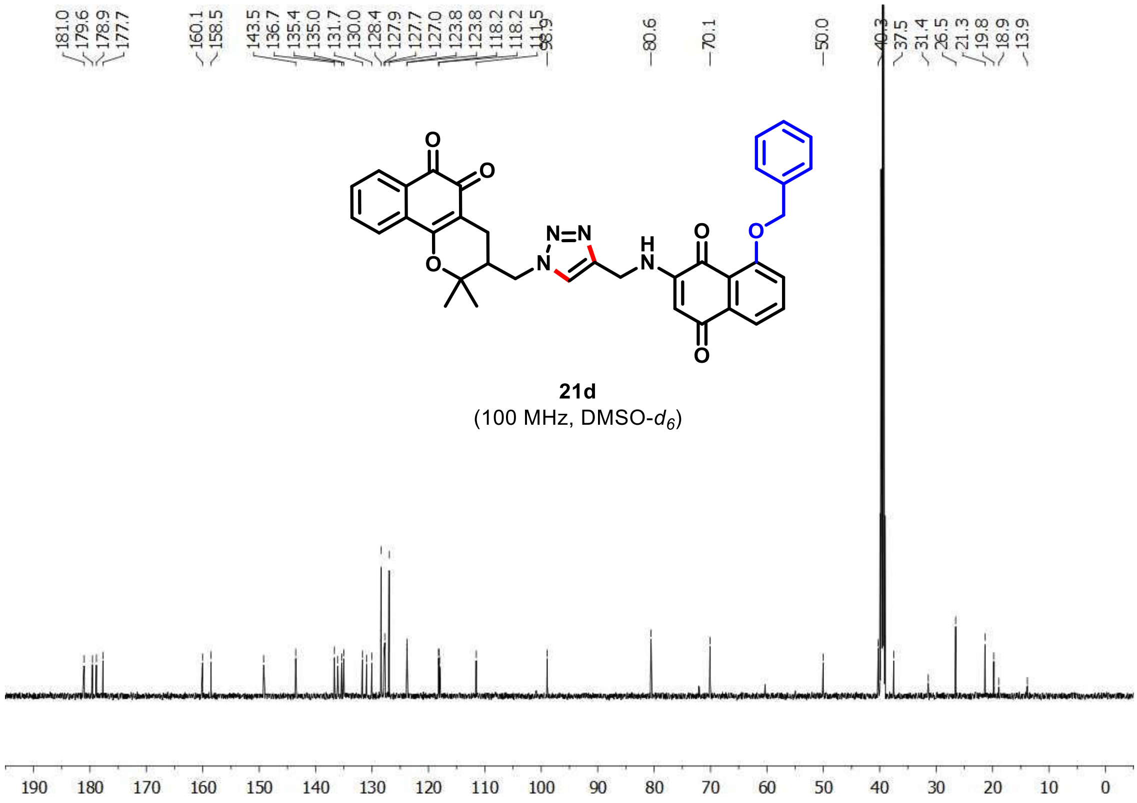
21d
(100 MHz, DMSO-d6)

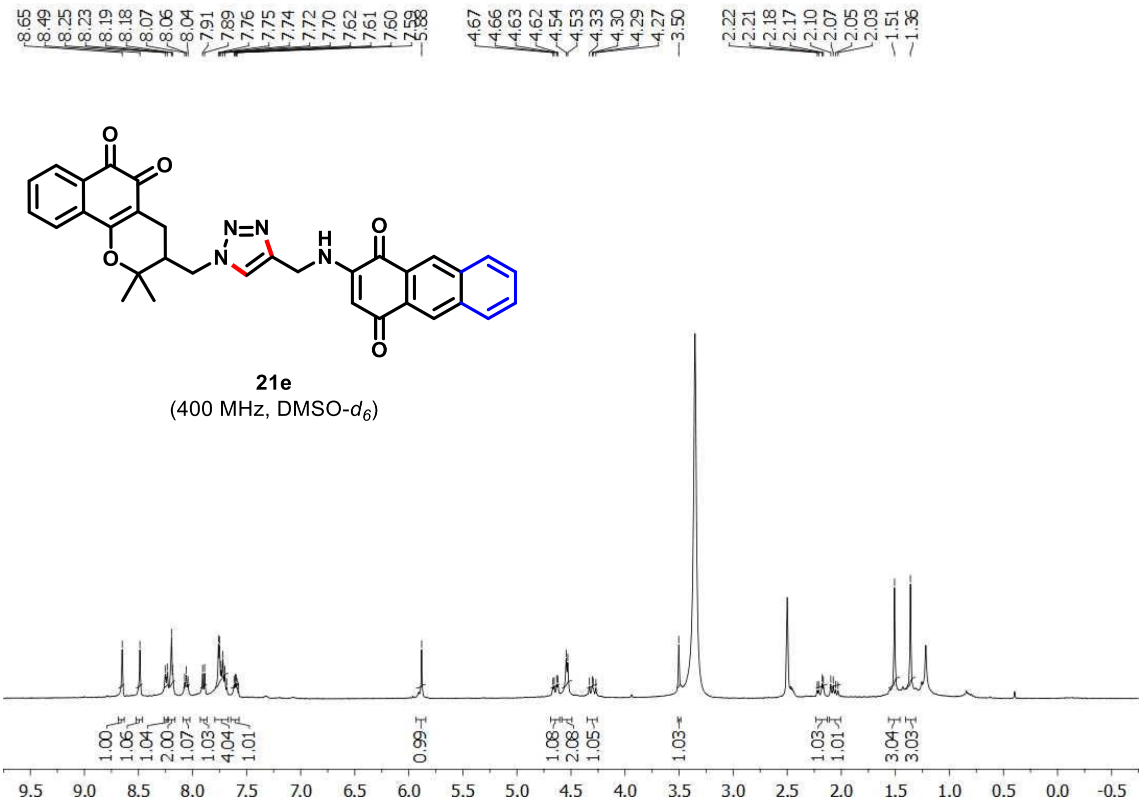
8.65 8.49 8.25 8.23 8.19 8.18 8.07 8.06 8.04 7.91 7.89 7.76 7.75 7.74 7.72 7.70 7.62 7.61 7.60 7.59 5.88
4.67 4.66 4.63 4.62 4.54 4.53 4.33 4.30 4.29 4.27 3.50
2.22 2.21 2.18 2.17 2.10 2.07 2.05 2.03 1.51 1.36
21e
(400 MHz, DMSO-d_6)
1.00 1.06 1.04 2.00 1.07 1.03 4.04 1.01 0.99 1.08 2.08 1.05 1.03 1.03 1.01 3.04 3.03
9.5 9.0 8.5 8.0 7.5 7.0 6.5 6.0 5.5 5.0 4.5 4.0 3.5 3.0 2.5 2.0 1.5 1.0 0.5 0.0 -0.5

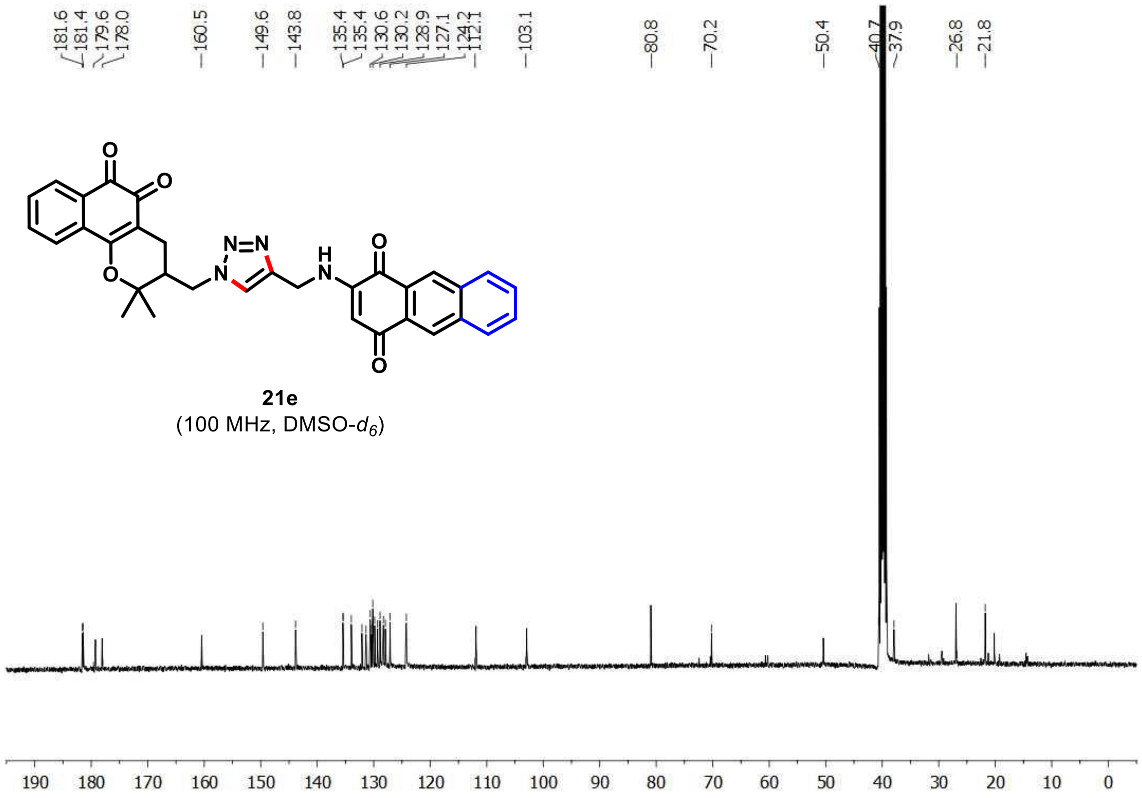
181.6 181.4 179.6 178.0 160.5 149.6 143.8 135.4 135.4 130.6 130.2 128.9 127.1 124.2 112.1 103.1 80.8 70.2 50.4 40.7 37.9 26.8 21.8
21e
(100 MHz, DMSO-d_6)
190 180 170 160 150 140 130 120 110 100 90 80 70 60 50 40 30 20 10 0
